# Supplementary material for: Protein–Protein Interactions Efficiently Modeled by Residue Cluster Classes
Source: Int J Mol Sci. 2020 Jul 6;21(13):4787. doi: 10.3390/ijms21134787 (PMC7370293; doi:10.3390/ijms21134787)
Supplement: Supplementary file 1 [file ijms-21-04787-s001.pdf]

Table 1. Hyper parameters for best models obtained with AutoWeka using full training sets.

RCC	ClassifierName	Arguments	AttributeSearch	Attribute SearchArg	AttributeEval	AttributeEvalArgs
10NoLat_Sum	weka.classifiers.lazy.IBk	[-E, -K, 1, -I]	weka.attributeSelection.GreedyStepwise	[-C, -B, -R]	weka.attributeSelection.CfsSubsetEval	[-M, -L]
10NoLat_Concat	weka.classifiers.meta.RandomSubSpace	[-I, 4, -P, 0.7735350708070846, -S, 1, -W, weka.classifiers.trees.J48, --, -O, -B, -M, 1, -C, 0.364431932692619]	null	[]	null	[]
10YesLat_Sum	weka.classifiers.lazy.IBk	[-E, -K, 2]	weka.attributeSelection.GreedyStepwise	[-C, -R]	weka.attributeSelection.CfsSubsetEval	[]
10YesLat_Concat	weka.classifiers.trees.RandomForest	[-I, 10, -K, 0, -depth, 0]	null	[]	null	[]
11NoLat_Sum	weka.classifiers.lazy.IBk	[-K, 2, -X, -F]	weka.attributeSelection.GreedyStepwise	[-B, -R]	weka.attributeSelection.CfsSubsetEval	[-M]
11NoLat_Concat	weka.classifiers.trees.RandomForest	[-I, 10, -K, 0, -depth, 0]	null	[]	null	[]
11YesLat_Sum	weka.classifiers.trees.RandomForest	[-I, 10, -K, 0, -depth, 0]	null	[]	null	[]
11YesLat_Concat	weka.classifiers.trees.RandomForest	[-I, 10, -K, 0, -depth, 0]	null	[]	null	[]
12NoLat_Sum	weka.classifiers.lazy.IBk	[-E, -K, 4, -I]	null	[]	null	[]
12NoLat_Concat	weka.classifiers.rules.DecisionTable	[-E, acc, -S, weka.attributeSelection.BestFirst, -X, 1]	null	[]	null	[]
12YesLat_Sum	weka.classifiers.trees.RandomForest	[-I, 10, -K, 0, -depth, 0]	null	[]	null	[]
12YesLat_Concat	weka.classifiers.trees.RandomForest	[-I, 10, -K, 0, -depth, 0]	null	[]	null	[]
13NoLat_Sum	weka.classifiers.trees.RandomForest	[-I, 10, -K, 0, -depth, 0]	null	[]	null	[]
13NoLat_Concat	weka.classifiers.trees.RandomForest	[-I, 10, -K, 0, -depth, 0]	null	[]	null	[]
13YesLat_Sum	weka.classifiers.trees.RandomForest	[-I, 10, -K, 0, -depth, 0]	null	[]	null	[]
13YesLat_Concat	weka.classifiers.trees.RandomForest	[-I, 10, -K, 0, -depth, 0]	null	[]	null	[]
14NoLat_Sum	weka.classifiers.trees.RandomForest	[-I, 10, -K, 0, -depth, 0]	null	[]	null	[]
14NoLat_Concat	weka.classifiers.trees.RandomForest	[-I, 10, -K, 0, -depth, 0]	null	[]	null	[]
14YesLat_Sum	weka.classifiers.trees.RandomForest	[-I, 10, -K, 0, -depth, 0]	null	[]	null	[]
14YesLat_Concat	weka.classifiers.trees.RandomForest	[-I, 10, -K, 0, -depth, 0]	null	[]	null	[]
15NoLat_Sum	weka.classifiers.trees.RandomForest	[-I, 10, -K, 0, -depth, 0]	null	[]	null	[]
15NoLat_Concat	weka.classifiers.trees.RandomForest	[-I, 10, -K, 0, -depth, 0]	null	[]	null	[]
15YesLat_Sum	weka.classifiers.trees.RandomForest	[-I, 10, -K, 0, -depth, 0]	null	[]	null	[]
15YesLat_Concat	weka.classifiers.trees.RandomForest	[-I, 10, -K, 0, -depth, 0]	null	[]	null	[]
4NoLat_Sum	weka.classifiers.trees.RandomForest	[-I, 10, -K, 0, -depth, 0]	null	[]	null	[]
4NoLat_Concat	weka.classifiers.rules.PART	[-M, 1, -B]	weka.attributeSelection.Greedy	[-R]	weka.attributeSelection.CfsSub	[]

			Stepwise		setEval	
4YesLat_Sum	weka.classifiers.trees.RandomForest	[-I, 10, -K, 0, -depth, 0]	null	[]	null	[]
4YesLat_Concat	weka.classifiers.trees.RandomForest	[-I, 10, -K, 0, -depth, 0]	null	[]	null	[]
5NoLat_Sum	weka.classifiers.functions.SMO	[-C, 1.4330986261650431, -N, 0, -K, weka.classifiers.functions.supportVector.PolyKernel -E 4.651016179097356 -L]	null	[]	null	[]
5NoLat_Concat	weka.classifiers.rules.PART	[-M, 2, -B]	weka.attributeSelection.GreedyStepwise	[-R]	weka.attributeSelection.CfsSubsetEval	[]
5YesLat_Sum	weka.classifiers.lazy.IBk	[-E, -K, 4, -X, -I]	null	[]	null	[]
5YesLat_Concat	weka.classifiers.lazy.IBk	[-E, -K, 28, -X, -F]	null	[]	null	[]
6NoLat_Sum	weka.classifiers.trees.RandomForest	[-I, 10, -K, 0, -depth, 0]	null	[]	null	[]
6NoLat_Concat	weka.classifiers.rules.PART	[-M, 2, -B]	weka.attributeSelection.GreedyStepwise	[-R]	weka.attributeSelection.CfsSubsetEval	[]
6YesLat_Sum	weka.classifiers.meta.AdaBoostM1	[-P, 100, -I, 83, -Q, -S, 1, -W, weka.classifiers.trees.J48, --, -B, -J, -A, -M, 4, -C, 0.35683272170421754]	weka.attributeSelection.GreedyStepwise	[-R]	weka.attributeSelection.CfsSubsetEval	[-L]
6YesLat_Concat	weka.classifiers.lazy.IBk	[-E, -K, 1, -I]	weka.attributeSelection.GreedyStepwise	[-C, -B, -R]	weka.attributeSelection.CfsSubsetEval	[-M, -L]
7NoLat_Sum	weka.classifiers.lazy.IBk	[-E, -K, 28, -X, -F]	null	[]	null	[]
7NoLat_Concat	weka.classifiers.trees.RandomForest	[-I, 10, -K, 0, -depth, 0]	null	[]	null	[]
7YesLat_Sum	weka.classifiers.lazy.IBk	[-E, -K, 28, -X, -F]	null	[]	null	[]
7YesLat_Concat	weka.classifiers.rules.DecisionTable	[-E, acc, -S, weka.attributeSelection.GreedyStepwise, -X, 1]	weka.attributeSelection.GreedyStepwise	[-C, -R]	weka.attributeSelection.CfsSubsetEval	[]
8NoLat_Sum	weka.classifiers.trees.RandomForest	[-I, 10, -K, 0, -depth, 0]	null	[]	null	[]
8NoLat_Concat	weka.classifiers.trees.RandomForest	[-I, 10, -K, 0, -depth, 0]	null	[]	null	[]
8YesLat_Sum	weka.classifiers.lazy.IBk	[-E, -K, 3, -I]	null	[]	null	[]
8YesLat_Concat	weka.classifiers.lazy.IBk	[-E, -K, 1, -I]	weka.attributeSelection.GreedyStepwise	[-C, -B, -R]	weka.attributeSelection.CfsSubsetEval	[-M, -L]
9NoLat_Sum	weka.classifiers.lazy.IBk	[-E, -K, 1, -X]	weka.attributeSelection.GreedyStepwise	[-C, -B, -R]	weka.attributeSelection.CfsSubsetEval	[-L]
9NoLat_Concat	weka.classifiers.lazy.LWL	[-K, 90, -A, weka.core.neighboursearch.LinearNNSearch, -W, weka.classifiers.trees.REPTree, --, -M, 3, -V, 6.929450822374736E-4, -L, 6, -P]	weka.attributeSelection.BestFirst	[-D, 0, -N, 2]	weka.attributeSelection.CfsSubsetEval	[]

9YesLat_Sum	weka.classifiers.trees.LMT	[-C, -P, -M, 2, -W, 0.4466263463898962]	weka.attributeSelection.GreedyStepwise	[-C, -B, -R]	weka.attributeSelection.CfsSubsetEval	[-L]
9YesLat_Concat	weka.classifiers.lazy.IBk	[-K, 1, -X, -I]	null	[]	null	[]

The RCC column describes the conditions used to build the corresponding RCCs. The first digits specifies the distance in Angstroms used to build the contact maps; YesLat or NoLat specifies if side-chain atomes were used or not; Sum or Concat specifies if addition of each RCC for a protein-protein pair was used of concatenation.

Table S2. Hyper parameters for best models obtained with AutoWeka using samplings of training sets with redundancy.

RCC	ClassifierName	Arguments	AttributeSearch	Attribute SearchArg	AttributeEval	Attribute EvalArgs
norm_7noSC_sum_sz1_it1_os200	weka.classifiers.meta.AdaBoostM1	[-P, 100, -I, 96, -Q, -S, 1, -W, weka.classifiers.trees.REPTree, --, -M, 1, -V, 1.577303729235641E-4, -L, -1, -P]weka.attributeSelection.GreedyStepwise	[-C, -B, -R]weka.attributeSelection.CfsSubsetEval	[-M, -L]		
norm_7noSC_sum_sz1_it2_os200	weka.classifiers.lazy.LWL	[-U, 4, -A, weka.core.neighboursearch.LinearNNSearch, -W, weka.classifiers.trees.RandomForest, --, -I, 96, -K, 5, -depth, 18]weka.attributeSelection.GreedyStepwise	[-C, -B, -R]weka.attributeSelection.CfsSubsetEval	[]		
norm_7noSC_sum_sz1_it3_os200	weka.classifiers.meta.AdaBoostM1	[-P, 100, -I, 19, -S, 1, -W, weka.classifiers.trees.RandomForest, --, -I, 21, -K, 6, -depth, 0]null	[]null	[]		
norm_7noSC_sum_sz1_it1_os200	weka.classifiers.meta.AdaBoostM1	[-P, 100, -I, 38, -S, 1, -W, weka.classifiers.bayes.BayesNet, --, -D, -Q, weka.classifiers.bayes.net.search.local.TAN]weka.attributeSelection.GreedyStepwise	[-C, -R]weka.attributeSelection.CfsSubsetEval	[-M, -L]		
norm_7noSC_sum_sz1_it2_os200	weka.classifiers.meta.AdaBoostM1	[-P, 100, -I, 10, -S, 1, -W, weka.classifiers.trees.J48, --, -B, -S, -M, 1, -C, 0.10125807590986646]weka.attributeSelection.GreedyStepwise	[-B, -R]weka.attributeSelection.CfsSubsetEval	[-L]		
norm_7noSC_sum_sz1_it3_os200	weka.classifiers.lazy.LWL	[-K, 90, -A, weka.core.neighboursearch.LinearNNSearch, -W, weka.classifiers.fun	[]null	[]		

		ctions.MultilayerPerceptron, --, -L, 0.8887372979649986, -M, 0.5949245081853208, -H, t, -R, -S, 1]null		
norm_7yesSC_sum_sz1_it1_os200	weka.classifiers.meta.AdaBoostM1	[-P, 100, -I, 11, -S, 1, -W, weka.classifiers.trees.LMT, --, -B, -C, -M, 1, -W, 0.009659721272996746, -A]null	[]null	[]
norm_7yesSC_sum_sz1_it2_os200	weka.classifiers.lazy.LWL	[-K, 90, -A, weka.core.neighboursearch.LinearNNSearch, -W, weka.classifiers.functions.Logistic, --, -R, 0.2577812797387019]null	[]null	[]
norm_7yesSC_sum_sz1_it3_os200	weka.classifiers.meta.AdaBoostM1	[-P, 100, -I, 38, -S, 1, -W, weka.classifiers.trees.RandomForest, --, -I, 2, -K, 5, -depth, 15]weka.attributeSelection.GreedyStepwise	[-C, -B, -R]weka.attributeSelection.CfsSubsetEval	[-L]
norm_7yesSC_sum_sz1_it1_os200	weka.classifiers.lazy.IBk	[-E, -K, 31, -X, -I]null	[]null	[]
norm_7yesSC_sum_sz1_it2_os200	weka.classifiers.lazy.IBk	[-K, 2]null	[]null	[]
norm_7yesSC_sum_sz1_it3_os200	weka.classifiers.lazy.LWL	[-A, weka.core.neighboursearch.LinearNNSearch, -W, weka.classifiers.lazy.IBk, --, -K, 56, -X, -I]null	[]null	[]
norm_8noSC_sum_sz1_it1_os200	weka.classifiers.lazy.LWL	[-K, 60, -A, weka.core.neighboursearch.LinearNNSearch, -W, weka.classifiers.functions.MultilayerPerceptron, --, -L, 0.7916844298137455, -M, 0.15755567975033988, -B, -H, t, -S, 1]null	[]null	[]
norm_8noSC_sum_sz1_it2_os200	weka.classifiers.meta.RandomCommittee	[-I, 2, -S, 1, -W, weka.classifiers.trees.RandomForest, --, -I, 19, -K, 0, -depth, 0]weka.attributeSelection.GreedyStepwise	[-B, -N, 13]weka.attributeSelection.CfsSubsetEval	[-L]
norm_8noSC_sum_sz1_it3_os200	weka.classifiers.lazy.LWL	[-K, 90, -A, weka.core.neighboursearch.LinearNNSearch, -W, weka.classifiers.functions.SimpleLogist	[]null	[]

		ic, --, -S, -W, 0.14991094330775467]null		
norm_8noSC_sum_sz1_it1_os200	weka.classifiers.lazy.IBk	[-E, -K, 34, -X, -I]null	[]null	[]
norm_8noSC_sum_sz1_it2_os200	weka.classifiers.lazy.IBk	[-K, 1, -X, -I]null	[]null	[]
norm_8noSC_sum_sz1_it3_os200	weka.classifiers.lazy.IBk	[-K, 2, -X, -F]weka.attributeSelection.GreedyStepwise	[-B, -R]weka.attributeSelection.CfsSubsetEval	[-M]
norm_8yesSC_sum_sz1_it1_os200	weka.classifiers.trees.RandomForest	[-I, 10, -K, 0, -depth, 0]null	[]null	[]
norm_8yesSC_sum_sz1_it2_os200	weka.classifiers.meta.RandomSubSpace	[-I, 19, -P, 0.8528725896677177, -S, 1, -W, weka.classifiers.trees.RandomTree, --, -M, 1, -K, 8, -depth, 0, -N, 0]null	[]null	[]
norm_8yesSC_sum_sz1_it3_os200	weka.classifiers.meta.RandomSubSpace	[-I, 19, -P, 0.8528725896677177, -S, 1, -W, weka.classifiers.trees.RandomTree, --, -M, 1, -K, 8, -depth, 0, -N, 0]null	[]null	[]
norm_8yesSC_sum_sz1_it1_os200	weka.classifiers.bayes.BayesNet	[-D, -Q, weka.classifiers.bayes.net.search.local.LAGDHillClimber]null	[]null	[]
norm_8yesSC_sum_sz1_it2_os200	weka.classifiers.bayes.BayesNet	[-D, -Q, weka.classifiers.bayes.net.search.local.LAGDHillClimber]weka.attributeSelection.GreedyStepwise	[-C, -R]weka.attributeSelection.CfsSubsetEval	[]
norm_8yesSC_sum_sz1_it3_os200	weka.classifiers.lazy.IBk	[-K, 3, -X, -I]weka.attributeSelection.GreedyStepwise	[-C, -R]weka.attributeSelection.CfsSubsetEval	[-M, -L]
norm_7noSC_sum_sz1_it1_os300	weka.classifiers.trees.RandomForest	[-I, 159, -K, 26, -depth, 0]null	[]null	[]
norm_7noSC_sum_sz1_it2_os300	weka.classifiers.lazy.LWL	[-K, -1, -A, weka.core.neighboursearch.LinearNNSearch, -W, weka.classifiers.trees.RandomForest, --, -I, 81, -K, 3, -depth, 0]weka.attributeSelection.GreedyStepwise	[-C, -B, -R]weka.attributeSelection.CfsSubsetEval	[]
norm_7noSC_sum_sz1_it3_os300	weka.classifiers.meta.AdaBoostM1	[-P, 100, -I, 28, -S, 1, -W, weka.classifiers.trees.LMT, --, -B, -M, 12, -W, 0, -A]weka.attributeSelection.GreedyStepwise	[-B, -R]weka.attributeSelection.CfsSubsetEval	[-L]
norm_7noSC_sum_sz1_it1_os300	weka.classifiers.lazy.IBk	[-K, 2]null	[]null	[]

norm_7noSC_sum_sz1_it2_os300	weka.classifiers.bayes.BayesNet	[-D, -Q, weka.classifiers.bayes.net.search.local.K2]null	[]null	[]
norm_7noSC_sum_sz1_it3_os300	weka.classifiers.meta.AdaBoostM1	[-P, 100, -I, 83, -Q, -S, 1, -W, weka.classifiers.trees.J48, --, -B, -J, -A, -M, 4, -C, 0.35683272170421754]weka.attributeSelection.GreedyStepwise	[-R]weka.attributeSelection.CfsSubsetEval	[-L]
norm_7yesSC_sum_sz1_it1_os300	weka.classifiers.meta.AdaBoostM1	[-P, 99, -I, 123, -S, 1, -W, weka.classifiers.rules.JRip, --, -N, 3.748316259236546, -E, -O, 3]null	[]null	[]
norm_7yesSC_sum_sz1_it2_os300	weka.classifiers.meta.AdaBoostM1	[-P, 74, -I, 14, -Q, -S, 1, -W, weka.classifiers.trees.RandomForest, --, -I, 70, -K, 0, -depth, 0]null	[]null	[]
norm_7yesSC_sum_sz1_it3_os300	weka.classifiers.lazy.LWL	[-K, 90, -A, weka.core.neighboursearch.LinearNNSearch, -W, weka.classifiers.functions.SMO, --, -C, 1.1903405715171074, -N, 0, -M, -K, weka.classifiers.functions.supportVector.NormalizedPolyKernel -E 2.962042110507982]weka.attributeSelection.GreedyStepwise	[-R]weka.attributeSelection.CfsSubsetEval	[-L]
norm_7yesSC_sum_sz1_it1_os300	weka.classifiers.meta.AdaBoostM1	[-P, 93, -I, 91, -S, 1, -W, weka.classifiers.trees.J48, --, -O, -J, -S, -M, 1, -C, 0.26480960543806775]weka.attributeSelection.GreedyStepwise	[-C, -B, -R]weka.attributeSelection.CfsSubsetEval	[-M, -L]
norm_7yesSC_sum_sz1_it2_os300	weka.classifiers.lazy.IBk	[-E, -K, 4, -X, -I]null	[]null	[]
norm_7yesSC_sum_sz1_it3_os300	weka.classifiers.lazy.IBk	[-K, 2]null	[]null	[]
norm_8noSC_sum_sz1_it1_os300	weka.classifiers.lazy.LWL	[-K, 30, -A, weka.core.neighboursearch.LinearNNSearch, -W, weka.classifiers.functions.MultilayerPerceptron, --, -L, 0.9907373090659772, -M, 0.5480862494169916, -B, -H, i, -R, -S, 1]weka.attributeSel	[-B, -R]weka.attributeSelection.CfsSubsetEval	[-L]

		ection.GreedyStepwise		
norm_8noSC_sum_sz1_it2_os300	weka.classifiers.meta.AdaBoostM1	[-P, 82, -I, 95, -S, 1, -W, weka.classifiers.rules.PART, --, -N, 3, -M, 18, -R, -B]weka.attributeSelection.BestFirst	[-D, 0, -N, 2]weka.attributeSelection.CfsSubsetEval	[-M]
norm_8noSC_sum_sz1_it3_os300	weka.classifiers.meta.RandomCommittee	[-I, 38, -S, 1, -W, weka.classifiers.trees.RandomTree, --, -M, 2, -K, 3, -depth, 20, -N, 0]weka.attributeSelection.GreedyStepwise	[-C, -B, -R]weka.attributeSelection.CfsSubsetEval	[-M, -L]
norm_8noSC_sum_sz1_it1_os300	weka.classifiers.lazy.IBk	[-E, -K, 22, -X, -I]null	[]null	[]
norm_8noSC_sum_sz1_it2_os300	weka.classifiers.lazy.IBk	[-K, 2]null	[]null	[]
norm_8noSC_sum_sz1_it3_os300	weka.classifiers.bayes.BayesNet	[-D, -Q, weka.classifiers.bayes.net.search.local.LAGDHillClimber]null	[]null	[]
norm_8yesSC_sum_sz1_it1_os300	weka.classifiers.trees.RandomForest	[-I, 10, -K, 0, -depth, 0]null	[]null	[]
norm_8yesSC_sum_sz1_it2_os300	weka.classifiers.lazy.LWL	[-U, 4, -A, weka.core.neighboursearch.LinearNNSearch, -W, weka.classifiers.trees.RandomForest, --, -I, 96, -K, 5, -depth, 18]weka.attributeSelection.GreedyStepwise	[-C, -B, -R]weka.attributeSelection.CfsSubsetEval	[]
norm_8yesSC_sum_sz1_it3_os300	weka.classifiers.functions.SMO	[-C, 0.9552219744919479, -N, 1, -K, weka.classifiers.functions.supportVector.RBFKernel -G 0.5229674418898211]null	[]null	[]
norm_8yesSC_sum_sz1_it1_os300	weka.classifiers.lazy.LWL	[-K, 30, -A, weka.core.neighboursearch.LinearNNSearch, -W, weka.classifiers.trees.RandomForest, --, -I, 18, -K, 8, -depth, 6]null	[]null	[]
norm_8yesSC_sum_sz1_it2_os300	weka.classifiers.lazy.IBk	[-K, 2]null	[]null	[]
norm_8yesSC_sum_sz1_it3_os300	weka.classifiers.lazy.IBk	[-K, 2]null	[]null	[]
norm_7noSC_sum_sz1_it1_os300	weka.classifiers.lazy.LWL	[-U, 2, -A, weka.core.neighboursearch.LinearNNSearch, -W, weka.classifiers.trees.RandomForest, --, -I, 42, -K, 0, -depth,	[]null	[]

		0]null		
norm_7noSC_sum_sz1_it2_os300	weka.classifiers.lazy.LWL	[-A, weka.core.neighboursearch.LinearNNSearch, -W, weka.classifiers.trees.RandomForest, --, -I, 129, -K, 1, -depth, 0]null	[]null	[]
norm_7noSC_sum_sz1_it3_os300	weka.classifiers.meta.AdaBoostM1	[-P, 100, -I, 32, -Q, -S, 1, -W, weka.classifiers.trees.J48, --, -B, -S, -M, 1, -C, 0.6579615394211651]null	[]null	[]
norm_7noSC_sum_sz2_it1_os300	weka.classifiers.meta.RandomCommittee	[-I, 6, -S, 1, -W, weka.classifiers.trees.RandomForest, --, -I, 81, -K, 3, -depth, 0]null	[]null	[]
norm_7noSC_sum_sz2_it2_os300	weka.classifiers.meta.AdaBoostM1	[-P, 100, -I, 28, -S, 1, -W, weka.classifiers.trees.RandomForest, --, -I, 5, -K, 5, -depth, 0]null	[]null	[]
norm_7noSC_sum_sz2_it3_os300	weka.classifiers.meta.AdaBoostM1	[-P, 100, -I, 79, -S, 1, -W, weka.classifiers.rules.PART, --, -M, 1, -B]null	[]null	[]
norm_7noSC_sum_sz3_it1_os300	weka.classifiers.lazy.LWL	[-U, 2, -A, weka.core.neighboursearch.LinearNNSearch, -W, weka.classifiers.trees.RandomForest, --, -I, 135, -K, 3, -depth, 0]weka.attributeSelection.GreedyStepwise	[-C, -B, -R]weka.attributeSelection.CfsSubsetEval	[-M]
norm_7noSC_sum_sz3_it2_os300	weka.classifiers.trees.RandomForest	[-I, 222, -K, 3, -depth, 0]weka.attributeSelection.GreedyStepwise	[-C, -R]weka.attributeSelection.CfsSubsetEval	[-M, -L]
norm_7noSC_sum_sz3_it3_os300	weka.classifiers.lazy.LWL	[-K, 60, -A, weka.core.neighboursearch.LinearNNSearch, -W, weka.classifiers.functions.SMO, --, -C, 0.6151164517186112, -N, 2, -M, -K, weka.classifiers.functions.supportVector.PolyKernel -E 0.6262243036004508 -L]weka.attributeSelection.GreedyStepwise	[-R]weka.attributeSelection.CfsSubsetEval	[-M, -L]
norm_7noSC_sum_sz1_it1_os300	weka.classifiers.meta.RandomSub	[-I, 36, -P, 0.2757331204049802	[]null	[]

	Space	, -S, 1, -W, weka.classifiers.lazy.IBk, --, -E, -K, 10, -X]null		
norm_7noSC_sum_sz1_it2_os300	weka.classifiers.lazy.LWL	[-K, 90, -A, weka.core.neighboursearch.LinearNNSearch, -W, weka.classifiers.bayes.BayesNet, --, -Q, weka.classifiers.bayes.net.search.local.TAN]null	[]null	[]
norm_7noSC_sum_sz1_it3_os300	weka.classifiers.meta.Bagging	[-P, 85, -I, 72, -S, 1, -W, weka.classifiers.lazy.KStar, --, -B, 51, -M, d]null	[]null	[]
norm_7noSC_sum_sz2_it1_os300	weka.classifiers.meta.RandomCommittee	[-I, 14, -S, 1, -W, weka.classifiers.trees.RandomForest, --, -I, 59, -K, 1, -depth, 0]null	[]null	[]
norm_7noSC_sum_sz2_it2_os300	weka.classifiers.meta.RandomCommittee	[-I, 37, -S, 1, -W, weka.classifiers.trees.RandomForest, --, -I, 68, -K, 0, -depth, 19]weka.attributeSelection.GreedyStepwise	[-B, -R]weka.attributeSelection.CfsSubsetEval	[-L]
norm_7noSC_sum_sz2_it3_os300	weka.classifiers.trees.RandomForest	[-I, 159, -K, 26, -depth, 0]null	[]null	[]
norm_7noSC_sum_sz3_it1_os300	weka.classifiers.lazy.KStar	[-B, 69, -M, n]null	[]null	[]
norm_7noSC_sum_sz3_it2_os300	weka.classifiers.meta.AdaBoostM1	[-P, 100, -I, 83, -Q, -S, 1, -W, weka.classifiers.trees.J48, --, -B, -J, -A, -M, 4, -C, 0.35683272170421754]weka.attributeSelection.GreedyStepwise	[-R]weka.attributeSelection.CfsSubsetEval	[-L]
norm_7noSC_sum_sz3_it3_os300	weka.classifiers.lazy.LWL	[-K, 120, -A, weka.core.neighboursearch.LinearNNSearch, -W, weka.classifiers.trees.RandomForest, --, -I, 6, -K, 0, -depth, 0]null	[]null	[]
norm_7yesSC_sum_sz1_it1_os300	weka.classifiers.meta.AdaBoostM1	[-P, 100, -I, 78, -Q, -S, 1, -W, weka.classifiers.trees.RandomForest, --, -I, 8, -K, 0, -depth, 0]null	[]null	[]
norm_7yesSC_sum_sz1_it2_os300	weka.classifiers.lazy.LWL	[-K, 120, -A, weka.core.neighboursearch.LinearNNSearch, -W, weka.classifiers.functions.MultilayerPerceptron, --, -L, 0.9393330868351162	[]null	[]

		, -M, 0.6464897520337939, -B, -H, i, -C, -R, -D, -S, 1]null		
norm_7yesSC_sum_sz1_it3_os300	weka.classifiers.meta.AdaBoostM1	[-P, 100, -I, 15, -Q, -S, 1, -W, weka.classifiers.trees.RandomForest, --, -I, 115, -K, 0, -depth, 0]null	[]null	[]
norm_7yesSC_sum_sz2_it1_os300	weka.classifiers.meta.RandomCommittee	[-I, 3, -S, 1, -W, weka.classifiers.trees.RandomForest, --, -I, 30, -K, 1, -depth, 17]weka.attributeSelection.GreedyStepwise	[-C, -B, -R]weka.attributeSelection.CfsSubsetEval	[]
norm_7yesSC_sum_sz2_it2_os300	weka.classifiers.meta.AdaBoostM1	[-P, 100, -I, 64, -S, 1, -W, weka.classifiers.trees.LMT, --, -B, -R, -P, -M, 1, -W, 0]null	[]null	[]
norm_7yesSC_sum_sz2_it3_os300	weka.classifiers.meta.RandomCommittee	[-I, 24, -S, 1, -W, weka.classifiers.trees.RandomForest, --, -I, 44, -K, 0, -depth, 0]null	[]null	[]
norm_7yesSC_sum_sz3_it1_os300	weka.classifiers.lazy.LWL	[-K, 90, -A, weka.core.neighboursearch.LinearNNSearch, -W, weka.classifiers.functions.SMO, --, -C, 0.720534324424765, -N, 0, -M, -K, weka.classifiers.functions.supportVector.NormalizedPolyKernel -E 3.5872148769457537]null	[]null	[]
norm_7yesSC_sum_sz3_it2_os300	weka.classifiers.meta.AdaBoostM1	[-P, 100, -I, 14, -Q, -S, 1, -W, weka.classifiers.trees.RandomForest, --, -I, 225, -K, 2, -depth, 0]weka.attributeSelection.GreedyStepwise	[-B, -R]weka.attributeSelection.CfsSubsetEval	[-M]
norm_7yesSC_sum_sz3_it3_os300	weka.classifiers.meta.RandomCommittee	[-I, 50, -S, 1, -W, weka.classifiers.trees.RandomForest, --, -I, 30, -K, 1, -depth, 0]null	[]null	[]
norm_7yesSC_sum_sz1_it1_os300	weka.classifiers.meta.RandomCommittee	[-I, 27, -S, 1, -W, weka.classifiers.trees.RandomTree, --, -M, 1, -K, 5, -depth, 15, -N, 0]null	[]null	[]
norm_7yesSC_sum_sz1_it2_os300	weka.classifiers.lazy.KStar	[-B, 58, -M, n]null	[]null	[]
norm_7yesSC_sum_sz1_it3_os300	weka.classifiers.meta.RandomCommittee	[-I, 2, -S, 1, -W, weka.classifiers.trees.RandomForest, --, -I, 22, -K, 0, -depth,	[]null	[]

		0]null		
norm_7yesSC_sum_sz2_it1_os300	weka.classifiers.lazy.LWL	[-A, weka.core.neighboursearch.LinearNNSearch, -W, weka.classifiers.trees.RandomForest, --, -I, 27, -K, 0, -depth, 0]null	[]null	[]
norm_7yesSC_sum_sz2_it2_os300	weka.classifiers.meta.Bagging	[-P, 94, -I, 34, -S, 1, -W, weka.classifiers.lazy.KStar, --, -B, 40, -M, a]null	[]null	[]
norm_7yesSC_sum_sz2_it3_os300	weka.classifiers.lazy.LWL	[-U, 3, -A, weka.core.neighboursearch.LinearNNSearch, -W, weka.classifiers.rules.DecisionTable, --, -E, acc, -S, weka.attributeSelection.BestFirst, -X, 1]weka.attributeSelection.GreedyStepwise	[-B, -R]weka.attributeSelection.CfsSubsetEval	[-L]
norm_7yesSC_sum_sz3_it1_os300	weka.classifiers.meta.RandomCommittee	[-I, 12, -S, 1, -W, weka.classifiers.trees.RandomTree, --, -M, 2, -K, 0, -depth, 0, -N, 0, -U]null	[]null	[]
norm_7yesSC_sum_sz3_it2_os300	weka.classifiers.lazy.LWL	[-U, 3, -A, weka.core.neighboursearch.LinearNNSearch, -W, weka.classifiers.rules.DecisionTable, --, -E, acc, -S, weka.attributeSelection.BestFirst, -X, 1]weka.attributeSelection.GreedyStepwise	[-B, -R]weka.attributeSelection.CfsSubsetEval	[-L]
norm_7yesSC_sum_sz3_it3_os300	weka.classifiers.meta.AdaBoostM1	[-P, 100, -I, 51, -S, 1, -W, weka.classifiers.rules.JRip, --, -N, 2.0216181692968713, -P, -O, 5]null	[]null	[]
norm_8noSC_sum_sz1_it1_os300	weka.classifiers.lazy.LWL	[-U, 1, -A, weka.core.neighboursearch.LinearNNSearch, -W, weka.classifiers.functions.SMO, --, -C, 1.376271748154589, -N, 0, -M, -K, weka.classifiers.functions.supportVector.Puk -S 0.3024853148573099 -O 0.6878560663513378]null	[]null	[]
norm_8noSC_sum_sz1_it2_os300	weka.classifiers.lazy.LWL	[-A, weka.core.neighbo	[-R]weka.attribut	[-L]

		ursearch.LinearNNSearch, -W, weka.classifiers.functions.SMO, --, -C, 1.40118909891787, -N, 1, -M, -K, weka.classifiers.functions.supportVector.Puk -S 5.21528294838149 -O 0.9593741033305174]weka.attributeSelection.GreedyStepwise	eSelection.CfsSubsetEval	
norm_8noSC_sum_sz1_it3_os300	weka.classifiers.lazy.LWL	[-K, 90, -A, weka.core.neighboursearch.LinearNNSearch, -W, weka.classifiers.functions.SMO, --, -C, 0.5529075568117299, -N, 0, -K, weka.classifiers.functions.supportVector.PolyKernel -E 4.136290079762315 -L]null	[]null	[]
norm_8noSC_sum_sz2_it1_os300	weka.classifiers.trees.RandomForest	[-I, 13, -K, 1, -depth, 0]null	[]null	[]
norm_8noSC_sum_sz2_it2_os300	weka.classifiers.lazy.LWL	[-K, 120, -A, weka.core.neighboursearch.LinearNNSearch, -W, weka.classifiers.bayes.NaiveBayes, --]null	[]null	[]
norm_8noSC_sum_sz2_it3_os300	weka.classifiers.lazy.LWL	[-A, weka.core.neighboursearch.LinearNNSearch, -W, weka.classifiers.functions.SMO, --, -C, 1.2998489720788773, -N, 1, -M, -K, weka.classifiers.functions.supportVector.Puk -S 7.919934020026973 -O 0.21252260221279118]null	[]null	[]
norm_8noSC_sum_sz3_it1_os300	weka.classifiers.meta.RandomCommittee	[-I, 3, -S, 1, -W, weka.classifiers.trees.RandomForest, --, -I, 30, -K, 1, -depth, 17]weka.attributeSelection.GreedyStepwise	[-C, -B, -R]weka.attributeSelection.CfsSubsetEval	[]
norm_8noSC_sum_sz3_it2_os300	weka.classifiers.lazy.LWL	[-K, 90, -A, weka.core.neighboursearch.LinearNNSearch, -W, weka.classifiers.functions.SimpleLogist	[]null	[]

		ic, --, -S, -W, 0]null		
norm_8noSC_sum_sz3_it3_os300	weka.classifiers.trees.RandomForest	[-I, 222, -K, 3, -depth, 0]weka.attributeSelection.GreedyStepwise	[-C, -R]weka.attributeSelection.CfsSubsetEval	[-M, -L]
norm_8noSC_sum_sz1_it1_os300	weka.classifiers.lazy.LWL	[-K, 90, -A, weka.core.neighboursearch.LinearNNSearch, -W, weka.classifiers.bayes.NaiveBayes, --]null	[]null	[]
norm_8noSC_sum_sz1_it2_os300	weka.classifiers.meta.RandomCommittee	[-I, 17, -S, 1, -W, weka.classifiers.trees.RandomTree, --, -M, 1, -K, 0, -depth, 0, -N, 0, -U]weka.attributeSelection.GreedyStepwise	[-C, -B, -R]weka.attributeSelection.CfsSubsetEval	[]
norm_8noSC_sum_sz1_it3_os300	weka.classifiers.meta.AdaBoostM1	[-P, 100, -I, 33, -S, 1, -W, weka.classifiers.trees.J48, --, -B, -J, -S, -M, 1, -C, 0.03894188441579016]weka.attributeSelection.BestFirst	[-D, 0, -N, 5]weka.attributeSelection.CfsSubsetEval	[-M, -L]
norm_8noSC_sum_sz2_it1_os300	weka.classifiers.meta.AdaBoostM1	[-P, 100, -I, 39, -S, 1, -W, weka.classifiers.trees.LMT, --, -C, -P, -M, 1, -W, 0]weka.attributeSelection.GreedyStepwise	[-B, -N, 31]weka.attributeSelection.CfsSubsetEval	[-M, -L]
norm_8noSC_sum_sz2_it2_os300	weka.classifiers.meta.RandomCommittee	[-I, 14, -S, 1, -W, weka.classifiers.trees.RandomForest, --, -I, 59, -K, 1, -depth, 0]null	[]null	[]
norm_8noSC_sum_sz2_it3_os300	weka.classifiers.lazy.LWL	[-K, 90, -A, weka.core.neighboursearch.LinearNNSearch, -W, weka.classifiers.trees.RandomForest, --, -I, 18, -K, 0, -depth, 0]null	[]null	[]
norm_8noSC_sum_sz3_it1_os300	weka.classifiers.trees.RandomForest	[-I, 159, -K, 26, -depth, 0]null	[]null	[]
norm_8noSC_sum_sz3_it2_os300	weka.classifiers.trees.RandomForest	[-I, 157, -K, 0, -depth, 0]weka.attributeSelection.GreedyStepwise	[-C, -B, -R]weka.attributeSelection.CfsSubsetEval	[-L]
norm_8noSC_sum_sz3_it3_os300	weka.classifiers.meta.RandomSubSpace	[-I, 44, -P, 0.5025850381097647, -S, 1, -W, weka.classifiers.rules.DecisionTable, --, -E, rmse, -S, weka.attributeSelection.GreedyStepwis	[-R]weka.attributeSelection.CfsSubsetEval	[-M]

		e, -X, 4]weka.attributeSelection.GreedyStepwise		
norm_8yesSC_sum_sz1_it1_os300	weka.classifiers.lazy.LWL	[-U, 0, -A, weka.core.neighboursearch.LinearNNSearch, -W, weka.classifiers.functions.SMO, --, -C, 1.0407481792571314, -N, 1, -K, weka.classifiers.functions.supportVector.Puk -S 2.1047871804508707 -O 0.25763929173984856]weka.attributeSelection.GreedyStepwise	[-R]weka.attributeSelection.CfsSubsetEval	[-L]
norm_8yesSC_sum_sz1_it2_os300	weka.classifiers.lazy.LWL	[-U, 1, -A, weka.core.neighboursearch.LinearNNSearch, -W, weka.classifiers.functions.SMO, --, -C, 1.376271748154589, -N, 0, -M, -K, weka.classifiers.functions.supportVector.Puk -S 0.3024853148573099 -O 0.6878560663513378]null	[]null	[]
norm_8yesSC_sum_sz1_it3_os300	weka.classifiers.lazy.LWL	[-U, 4, -A, weka.core.neighboursearch.LinearNNSearch, -W, weka.classifiers.functions.SMO, --, -C, 1.4296431262588447, -N, 1, -K, weka.classifiers.functions.supportVector.Puk -S 3.828431509993488 -O 0.8291772469375045]null	[]null	[]
norm_8yesSC_sum_sz2_it1_os300	weka.classifiers.meta.RandomSubSpace	[-I, 36, -P, 0.2757331204049802, -S, 1, -W, weka.classifiers.lazy.IBk, --, -E, -K, 10, -X]null	[]null	[]
norm_8yesSC_sum_sz2_it2_os300	weka.classifiers.functions.SMO	[-C, 0.8834901573983901, -N, 1, -K, weka.classifiers.functions.supportVector.Puk -S 2.70071737141395 -O 0.7960004287380918	[-B, -R]weka.attributeSelection.CfsSubsetEval	[-M]

		]weka.attributeSelection.GreedyStepwise		
norm_8yesSC_sum_sz2_it3_os300	weka.classifiers.lazy.LWL	[-K, 60, -A, weka.core.neighboursearch.LinearNNSearch, -W, weka.classifiers.functions.SimpleLogistic, --, -W, 0]weka.attributeSelection.GreedyStepwise	[-B, -R]weka.attributeSelection.CfsSubsetEval	[-M]
norm_8yesSC_sum_sz3_it1_os300	weka.classifiers.lazy.LWL	[-K, 60, -A, weka.core.neighboursearch.LinearNNSearch, -W, weka.classifiers.bayes.NaiveBayes, --]null	[]null	[]
norm_8yesSC_sum_sz3_it2_os300	weka.classifiers.meta.RandomSubSpace	[-I, 19, -P, 0.8528725896677177, -S, 1, -W, weka.classifiers.trees.RandomTree, --, -M, 1, -K, 8, -depth, 0, -N, 0]null	[]null	[]
norm_8yesSC_sum_sz3_it3_os300	weka.classifiers.lazy.LWL	[-K, 60, -A, weka.core.neighboursearch.LinearNNSearch, -W, weka.classifiers.functions.SMO, --, -C, 0.6151164517186112, -N, 2, -M, -K, weka.classifiers.functions.supportVector.PolyKernel -E 0.6262243036004508 -L]weka.attributeSelection.GreedyStepwise	[-R]weka.attributeSelection.CfsSubsetEval	[-M, -L]
norm_8yesSC_sum_sz1_it1_os300	weka.classifiers.meta.AdaBoostM1	[-P, 64, -I, 81, -Q, -S, 1, -W, weka.classifiers.lazy.KStar, --, -B, 54, -M, a]weka.attributeSelection.GreedyStepwise	[-R]weka.attributeSelection.CfsSubsetEval	[]
norm_8yesSC_sum_sz1_it2_os300	weka.classifiers.lazy.LWL	[-A, weka.core.neighboursearch.LinearNNSearch, -W, weka.classifiers.bayes.BayesNet, --, -D, -Q, weka.classifiers.bayes.net.search.local.K2]weka.attributeSelection.GreedyStepwise	[-R]weka.attributeSelection.CfsSubsetEval	[-L]
norm_8yesSC_sum_sz1_it3_os300	weka.classifiers.meta.AdaBoostM1	[-P, 67, -I, 82, -S, 1, -W, weka.classifiers.rul	[-B, -R]weka.attributeSelection.CfsSu	[]

		es.DecisionTable, --, -E, rmse, -S, weka.attributeSelection.GreedyStepwise, -X, 1]weka.attributeSelection.GreedyStepwise	bsetEval	
norm_8yesSC_sum_sz2_it1_os300	weka.classifiers.meta.RandomSubSpace	[-I, 33, -P, 0.4492426479154742, -S, 1, -W, weka.classifiers.trees.RandomForest, --, -I, 23, -K, 0, -depth, 20]weka.attributeSelection.GreedyStepwise	[-C, -B, -R]weka.attributeSelection.CfsSubsetEval	[-L]
norm_8yesSC_sum_sz2_it2_os300	weka.classifiers.lazy.LWL	[-A, weka.core.neighboursearch.LinearNNSearch, -W, weka.classifiers.rules.DecisionTable, --, -E, acc, -S, weka.attributeSelection.BestFirst, -X, 3]weka.attributeSelection.GreedyStepwise	[-C, -B, -R]weka.attributeSelection.CfsSubsetEval	[]
norm_8yesSC_sum_sz2_it3_os300	weka.classifiers.lazy.LWL	[-A, weka.core.neighboursearch.LinearNNSearch, -W, weka.classifiers.rules.DecisionTable, --, -E, acc, -S, weka.attributeSelection.BestFirst, -X, 3]weka.attributeSelection.GreedyStepwise	[-C, -B, -R]weka.attributeSelection.CfsSubsetEval	[]
norm_8yesSC_sum_sz3_it1_os300	weka.classifiers.lazy.LWL	[-A, weka.core.neighboursearch.LinearNNSearch, -W, weka.classifiers.rules.DecisionTable, --, -E, acc, -S, weka.attributeSelection.GreedyStepwise, -X, 2]null	[]null	[]
norm_8yesSC_sum_sz3_it2_os300	weka.classifiers.meta.RandomCommittee	[-I, 9, -S, 1, -W, weka.classifiers.trees.RandomTree, --, -M, 1, -K, 4, -depth, 0, -N, 0, -U]weka.attributeSelection.GreedyStepwise	[-C, -R]weka.attributeSelection.CfsSubsetEval	[-M]
norm_8yesSC_sum_sz3_it3_os300	weka.classifiers.lazy.LWL	[-A, weka.core.neighboursearch.LinearNNSearch, -W, weka.classifiers.trees.RandomForest, --, -I, 130, -K, 1, -	[]null	[]

		depth, 16]null		
raw_7noSC_sum_sz1_it1_os200	weka.classifiers.meta.AdaBoostM1	[-P, 100, -I, 83, -Q, -S, 1, -W, weka.classifiers.trees.J48, --, -B, -J, -A, -M, 4, -C, 0.35683272170421754]weka.attributeSelection.GreedyStepwise	[-R]weka.attributeSelection.CfsSubsetEval	[-L]
raw_7noSC_sum_sz1_it2_os200	weka.classifiers.meta.RandomCommittee	[-I, 4, -S, 1, -W, weka.classifiers.trees.RandomForest, --, -I, 40, -K, 0, -depth, 0]null	[]null	[]
raw_7noSC_sum_sz1_it3_os200	weka.classifiers.meta.RandomCommittee	[-I, 17, -S, 1, -W, weka.classifiers.trees.RandomForest, --, -I, 5, -K, 0, -depth, 0]weka.attributeSelection.GreedyStepwise	[-C, -R]weka.attributeSelection.CfsSubsetEval	[-M, -L]
raw_7noSC_sum_sz1_it1_os200	weka.classifiers.meta.RandomSubSpace	[-I, 3, -P, 0.8488221429233894, -S, 1, -W, weka.classifiers.lazy.IBk, --, -K, 5, -X, -F]weka.attributeSelection.GreedyStepwise	[-R]weka.attributeSelection.CfsSubsetEval	[-M]
raw_7noSC_sum_sz1_it2_os200	weka.classifiers.lazy.IBk	[-K, 46, -X, -F]null	[]null	[]
raw_7noSC_sum_sz1_it3_os200	weka.classifiers.meta.AdaBoostM1	[-P, 100, -I, 83, -Q, -S, 1, -W, weka.classifiers.trees.J48, --, -B, -J, -A, -M, 4, -C, 0.35683272170421754]weka.attributeSelection.GreedyStepwise	[-R]weka.attributeSelection.CfsSubsetEval	[-L]
raw_7yesSC_sum_sz1_it1_os200	weka.classifiers.lazy.LWL	[-K, 90, -A, weka.core.neighboursearch.LinearNNSearch, -W, weka.classifiers.functions.SimpleLogistic, --, -S, -W, 0.14991094330775467]null	[]null	[]
raw_7yesSC_sum_sz1_it2_os200	weka.classifiers.meta.AdaBoostM1	[-P, 97, -I, 27, -S, 1, -W, weka.classifiers.trees.J48, --, -A, -S, -M, 1]null	[]null	[]
raw_7yesSC_sum_sz1_it3_os200	weka.classifiers.meta.RandomCommittee	[-I, 35, -S, 1, -W, weka.classifiers.trees.RandomForest, --, -I, 37, -K, 2, -depth, 0]null	[]null	[]
raw_7yesSC_sum_sz1_it1_os200	weka.classifiers.lazy.LWL	[-K, 30, -A, weka.core.neighboursearch.LinearNNSearch, -W, weka.classifiers.bayes.NaiveBayesMulti	[-B, -R]weka.attributeSelection.CfsSubsetEval	[]

		nomial, --]weka.attributeSelection.GreedyStepwise		
raw_7yesSC_sum_sz1_it2_os200	weka.classifiers.lazy.LWL	[-U, 4, -A, weka.core.neighboursearch.LinearNNSearch, -W, weka.classifiers.lazy.IBk, --, -K, 1, -F]null	[]null	[]
raw_7yesSC_sum_sz1_it3_os200	weka.classifiers.meta.RandomSubSpace	[-I, 19, -P, 0.45447567182111925, -S, 1, -W, weka.classifiers.rules.JRip, --, -N, 2.8975580210549317, -E, -P, -O, 5]null	[]null	[]
raw_8noSC_sum_sz1_it1_os200	weka.classifiers.meta.RandomCommittee	[-I, 37, -S, 1, -W, weka.classifiers.trees.RandomForest, --, -I, 68, -K, 0, -depth, 19]weka.attributeSelection.GreedyStepwise	[-B, -R]weka.attributeSelection.CfsSubsetEval	[-L]
raw_8noSC_sum_sz1_it2_os200	weka.classifiers.meta.AdaBoostM1	[-P, 100, -I, 109, -Q, -S, 1, -W, weka.classifiers.trees.LMT, --, -B, -M, 1, -W, 0.34497374145084814]weka.attributeSelection.BestFirst	[-D, 0, -N, 5]weka.attributeSelection.CfsSubsetEval	[-L]
raw_8noSC_sum_sz1_it3_os200	weka.classifiers.lazy.LWL	[-K, 30, -A, weka.core.neighboursearch.LinearNNSearch, -W, weka.classifiers.functions.MultilayerPerceptron, --, -L, 0.9907373090659772, -M, 0.5480862494169916, -B, -H, i, -R, -S, 1]null	[]null	[]
raw_8noSC_sum_sz1_it1_os200	weka.classifiers.lazy.LWL	[-K, 90, -A, weka.core.neighboursearch.LinearNNSearch, -W, weka.classifiers.functions.SMO, --, -C, 0.6670881603326894, -N, 1, -M, -K, weka.classifiers.functions.supportVector.RBFKernel -G 6.695013075815808E-4]null	[]null	[]
raw_8noSC_sum_sz1_it2_os200	weka.classifiers.bayes.BayesNet	[-D, -Q, weka.classifiers.bayes.net.search.local.K2]null	[]null	[]
raw_8noSC_sum_sz1_it3_os200	weka.classifiers.meta.AdaBoostM1	[-P, 72, -I, 4, -S, 1, -W, weka.classifiers.bayes.BayesNet, --, -D,	[-C, -R]weka.attributeSelection.CfsSubsetEval	[]

		-Q, weka.classifiers.bayes.net.search.local.HillClimber]weka.attributeSelection.GreedyStepwise		
raw_8yesSC_sum_sz1_it1_os200	weka.classifiers.meta.RandomCommittee	[-I, 3, -S, 1, -W, weka.classifiers.trees.RandomForest, --, -I, 42, -K, 0, -depth, 0]weka.attributeSelection.GreedyStepwise	[-C, -R]weka.attributeSelection.CfsSubsetEval	[]
raw_8yesSC_sum_sz1_it2_os200	weka.classifiers.lazy.LWL	[-K, 60, -A, weka.core.neighboursearch.LinearNNSearch, -W, weka.classifiers.functions.SMO, --, -C, 1.4774738706315516, -N, 0, -M, -K, weka.classifiers.functions.supportVector.PolyKernel -E 1.3735691089570568]null	[]null	[]
raw_8yesSC_sum_sz1_it3_os200	weka.classifiers.meta.RandomSubSpace	[-I, 19, -P, 0.8528725896677177, -S, 1, -W, weka.classifiers.trees.RandomTree, --, -M, 1, -K, 8, -depth, 0, -N, 0]null	[]null	[]
raw_8yesSC_sum_sz1_it1_os200	weka.classifiers.lazy.LWL	[-K, 30, -A, weka.core.neighboursearch.LinearNNSearch, -W, weka.classifiers.bayes.NaiveBayesMultinomial, --]null	[]null	[]
raw_8yesSC_sum_sz1_it2_os200	weka.classifiers.lazy.LWL	[-K, 90, -A, weka.core.neighboursearch.LinearNNSearch, -W, weka.classifiers.bayes.BayesNet, --, -D, -Q, weka.classifiers.bayes.net.search.local.K2]null	[]null	[]
raw_8yesSC_sum_sz1_it3_os200	weka.classifiers.lazy.IBk	[-K, 2, -X, -F]weka.attributeSelection.GreedyStepwise	[-B, -R]weka.attributeSelection.CfsSubsetEval	[-M]
raw_7noSC_sum_sz1_it1_os300	weka.classifiers.meta.AdaBoostM1	[-P, 100, -I, 68, -S, 1, -W, weka.classifiers.rules.JRip, --, -N, 1.9943204346948638, -E, -P, -O, 2]null	[]null	[]
raw_7noSC_sum_sz1_it2_os300	weka.classifiers.lazy.LWL	[-U, 4, -A, weka.core.neighboursearch.LinearNNSearch, -W, weka.classifiers.functions.SMO, --, -C,	[]null	[]

		1.4296431262588447, -N, 1, -K, weka.classifiers.functions.supportVector.Puk -S 3.828431509993488 -O 0.8291772469375045]null		
raw_7noSC_sum_sz1_it3_os300	weka.classifiers.meta.AdaBoostM1	[-P, 100, -I, 21, -S, 1, -W, weka.classifiers.trees.J48, --, -O, -B, -J, -A, -S, -M, 5, -C, 0.28126165966481054]null	[]null	[]
raw_7noSC_sum_sz1_it1_os300	weka.classifiers.lazy.IBk	[-K, 2]null	[]null	[]
raw_7noSC_sum_sz1_it2_os300	weka.classifiers.bayes.BayesNet	[-Q, weka.classifiers.bayes.net.search.local.HillClimber]null	[]null	[]
raw_7noSC_sum_sz1_it3_os300	weka.classifiers.lazy.IBk	[-K, 2, -X, -F]weka.attributeSelection.GreedyStepwise	[-B, -R]weka.attributeSelection.CfsSubsetEval	[-M]
raw_7yesSC_sum_sz1_it1_os300	weka.classifiers.meta.AdaBoostM1	[-P, 99, -I, 42, -Q, -S, 1, -W, weka.classifiers.trees.RandomForest, --, -I, 208, -K, 3, -depth, 9]weka.attributeSelection.GreedyStepwise	[-B, -R]weka.attributeSelection.CfsSubsetEval	[-M, -L]
raw_7yesSC_sum_sz1_it2_os300	weka.classifiers.trees.RandomForest	[-I, 159, -K, 26, -depth, 0]null	[]null	[]
raw_7yesSC_sum_sz1_it3_os300	weka.classifiers.meta.AdaBoostM1	[-P, 100, -I, 56, -S, 1, -W, weka.classifiers.rules.JRip, --, -N, 3.827920376224205, -P, -O, 5]null	[]null	[]
raw_7yesSC_sum_sz1_it1_os300	weka.classifiers.lazy.IBk	[-K, 2]null	[]null	[]
raw_7yesSC_sum_sz1_it2_os300	weka.classifiers.meta.AdaBoostM1	[-P, 70, -I, 100, -S, 1, -W, weka.classifiers.trees.REPTree, --, -M, 33, -V, 4.0031898370275335E-4, -L, -1, -P]null	[]null	[]
raw_7yesSC_sum_sz1_it3_os300	weka.classifiers.lazy.KStar	[-B, 48, -M, n]null	[]null	[]
raw_8noSC_sum_sz1_it1_os300	weka.classifiers.lazy.LWL	[-U, 4, -A, weka.core.neighboursearch.LinearNNSearch, -W, weka.classifiers.functions.SMO, --, -C, 1.4296431262588447, -N, 1, -K, weka.classifiers.functions.supportVector.Puk -S	[]null	[]

		3.828431509993488 -O 0.8291772469375045]null		
raw_8noSC_sum_sz1_it2_os300	weka.classifiers.meta.RandomSubSpace	[-I, 33, -P, 0.4492426479154742, -S, 1, -W, weka.classifiers.trees.RandomForest, --, -I, 23, -K, 0, -depth, 20]weka.attributeSelection.GreedyStepwise	[-C, -B, -R]weka.attributeSelection.CfsSubsetEval	[-L]
raw_8noSC_sum_sz1_it3_os300	weka.classifiers.functions.SMO	[-C, 0.8834901573983901, -N, 1, -K, weka.classifiers.functions.supportVector.Puk -S 2.70071737141395 -O 0.7960004287380918]weka.attributeSelection.GreedyStepwise	[-B, -R]weka.attributeSelection.CfsSubsetEval	[-M]
raw_8noSC_sum_sz1_it1_os300	weka.classifiers.meta.AdaBoostM1	[-P, 100, -I, 83, -Q, -S, 1, -W, weka.classifiers.trees.RandomForest, --, -I, 185, -K, 1, -depth, 0]null	[]null	[]
raw_8noSC_sum_sz1_it2_os300	weka.classifiers.bayes.BayesNet	[-D, -Q, weka.classifiers.bayes.net.search.local.LAGDHillClimber]null	[]null	[]
raw_8noSC_sum_sz1_it3_os300	weka.classifiers.bayes.BayesNet	[-D, -Q, weka.classifiers.bayes.net.search.local.LAGDHillClimber]null	[]null	[]
raw_8yesSC_sum_sz1_it1_os300	weka.classifiers.meta.AdaBoostM1	[-P, 100, -I, 66, -Q, -S, 1, -W, weka.classifiers.trees.REPTree, --, -M, 2, -V, 0.09149994372759244, -L, -1, -P]null	[]null	[]
raw_8yesSC_sum_sz1_it2_os300	weka.classifiers.functions.SMO	[-C, 0.8834901573983901, -N, 1, -K, weka.classifiers.functions.supportVector.Puk -S 2.70071737141395 -O 0.7960004287380918]weka.attributeSelection.GreedyStepwise	[-B, -R]weka.attributeSelection.CfsSubsetEval	[-M]
raw_8yesSC_sum_sz1_it3_os300	weka.classifiers.meta.AdaBoostM1	[-P, 100, -I, 83, -S, 1, -W, weka.classifiers.trees.RandomForest, --, -I, 5, -K, 0, -depth, 15]null	[]null	[]

raw_8yesSC_sum_sz1_it1_os300	weka.classifiers.meta.AdaBoostM1	[-P, 82, -I, 95, -S, 1, -W, weka.classifiers.rules.PART, --, -N, 3, -M, 18, -R, -B]weka.attributeSelection.BestFirst	[-D, 0, -N, 2]weka.attributeSelection.CfsSubsetEval	[-M]
raw_8yesSC_sum_sz1_it2_os300	weka.classifiers.lazy.LWL	[-K, 60, -A, weka.core.neighboursearch.LinearNNSearch, -W, weka.classifiers.functions.Logistic, --, -R, 0.16765060417244187]weka.attributeSelection.GreedyStepwise	[-C, -B, -R]weka.attributeSelection.CfsSubsetEval	[-L]
raw_8yesSC_sum_sz1_it3_os300	weka.classifiers.lazy.IBk	[-K, 2]null	[]null	[]
raw_7noSC_sum_sz1_it1_os300	weka.classifiers.meta.AdaBoostM1	[-P, 100, -I, 64, -S, 1, -W, weka.classifiers.trees.LMT, --, -B, -R, -P, -M, 1, -W, 0]null	[]null	[]
raw_7noSC_sum_sz1_it2_os300	weka.classifiers.functions.SMO	[-C, 1.4706914365886425, -N, 1, -K, weka.classifiers.functions.supportVector.Puk -S 1.839051789593409 -O 0.8462457871776845]null	[]null	[]
raw_7noSC_sum_sz1_it3_os300	weka.classifiers.lazy.LWL	[-A, weka.core.neighboursearch.LinearNNSearch, -W, weka.classifiers.trees.RandomForest, --, -I, 50, -K, 6, -depth, 0]null	[]null	[]
raw_7noSC_sum_sz2_it1_os300	weka.classifiers.lazy.LWL	[-K, 30, -A, weka.core.neighboursearch.LinearNNSearch, -W, weka.classifiers.functions.Logistic, --, -R, 0.20363846548861847]null	[]null	[]
raw_7noSC_sum_sz2_it2_os300	weka.classifiers.meta.RandomCommittee	[-I, 14, -S, 1, -W, weka.classifiers.trees.RandomForest, --, -I, 59, -K, 1, -depth, 0]null	[]null	[]
raw_7noSC_sum_sz2_it3_os300	weka.classifiers.meta.RandomCommittee	[-I, 14, -S, 1, -W, weka.classifiers.trees.RandomForest, --, -I, 59, -K, 1, -depth, 0]null	[]null	[]
raw_7noSC_sum_sz3_it1_os300	weka.classifiers.meta.AdaBoostM1	[-P, 100, -I, 3, -Q, -S, 1, -W, weka.classifiers.trees.RandomForest, --,	[-C, -B, -R]weka.attributeSelection.CfsSubsetEval	[-M, -L]

		-I, 25, -K, 0, -depth, 0]weka.attributeSelection.GreedyStepwise		
raw_7noSC_sum_sz3_it2_os300	weka.classifiers.meta.RandomCommittee	[-I, 14, -S, 1, -W, weka.classifiers.trees.RandomForest, --, -I, 59, -K, 1, -depth, 0]null	[]null	[]
raw_7noSC_sum_sz3_it3_os300	weka.classifiers.lazy.LWL	[-K, 120, -A, weka.core.neighboursearch.LinearNNSearch, -W, weka.classifiers.functions.SimpleLogistic, --, -S, -W, 0.010617941783735652]null	[]null	[]
raw_7noSC_sum_sz1_it1_os300	weka.classifiers.lazy.LWL	[-K, 10, -A, weka.core.neighboursearch.LinearNNSearch, -W, weka.classifiers.bayes.NaiveBayes, --, -D]null	[]null	[]
raw_7noSC_sum_sz1_it2_os300	weka.classifiers.lazy.KStar	[-B, 16, -M, n]weka.attributeSelection.GreedyStepwise	[-C, -R]weka.attributeSelection.CfsSubsetEval	[]
raw_7noSC_sum_sz1_it3_os300	weka.classifiers.lazy.LWL	[-A, weka.core.neighboursearch.LinearNNSearch, -W, weka.classifiers.trees.RandomForest, --, -I, 73, -K, 0, -depth, 20]weka.attributeSelection.GreedyStepwise	[-R]weka.attributeSelection.CfsSubsetEval	[-M, -L]
raw_7noSC_sum_sz2_it1_os300	weka.classifiers.meta.AdaBoostM1	[-P, 100, -I, 15, -S, 1, -W, weka.classifiers.rules.DecisionTable, --, -E, auc, -I, -S, weka.attributeSelection.BestFirst, -X, 3]null	[]null	[]
raw_7noSC_sum_sz2_it2_os300	weka.classifiers.trees.RandomForest	[-I, 10, -K, 0, -depth, 0]null	[]null	[]
raw_7noSC_sum_sz2_it3_os300	weka.classifiers.meta.AdaBoostM1	[-P, 54, -I, 7, -S, 1, -W, weka.classifiers.trees.RandomForest, --, -I, 113, -K, 2, -depth, 0]weka.attributeSelection.GreedyStepwise	[-C, -B, -R]weka.attributeSelection.CfsSubsetEval	[-L]
raw_7noSC_sum_sz3_it1_os300	weka.classifiers.trees.RandomForest	[-I, 10, -K, 0, -depth, 0]null	[]null	[]
raw_7noSC_sum_sz3_it2_os300	weka.classifiers.meta.AdaBoostM1	[-P, 100, -I, 56, -S, 1, -W, weka.classifiers.rules.JRip, --, -N,	[]null	[]

		3.827920376224205, -P, -O, 5]null		
raw_7noSC_sum_sz3_it3_os300	weka.classifiers.lazy.KStar	[-B, 55, -M, a]null	[]null	[]
raw_7yesSC_sum_sz1_it1_os300	weka.classifiers.meta.AdaBoostM1	[-P, 100, -I, 110, -Q, -S, 1, -W, weka.classifiers.trees.RandomTree, --, -M, 13, -K, 0, -depth, 0, -N, 0, -U]null	[]null	[]
raw_7yesSC_sum_sz1_it2_os300	weka.classifiers.lazy.LWL	[-A, weka.core.neighboursearch.LinearNNSearch, -W, weka.classifiers.functions.SMO, --, -C, 0.868993574893975, -N, 1, -K, weka.classifiers.functions.supportVector.Puk -S 2.492701962846353 -O 0.35351749562266155]null	[]null	[]
raw_7yesSC_sum_sz1_it3_os300	weka.classifiers.trees.RandomForest	[-I, 26, -K, 1, -depth, 0]null	[]null	[]
raw_7yesSC_sum_sz2_it1_os300	weka.classifiers.meta.AdaBoostM1	[-P, 100, -I, 25, -Q, -S, 1, -W, weka.classifiers.trees.RandomForest, --, -I, 19, -K, 0, -depth, 0]weka.attributeSelection.GreedyStepwise	[-C, -R]weka.attributeSelection.CfsSubsetEval	[-M, -L]
raw_7yesSC_sum_sz2_it2_os300	weka.classifiers.meta.RandomCommittee	[-I, 14, -S, 1, -W, weka.classifiers.trees.RandomForest, --, -I, 59, -K, 1, -depth, 0]null	[]null	[]
raw_7yesSC_sum_sz2_it3_os300	weka.classifiers.trees.RandomForest	[-I, 222, -K, 3, -depth, 0]weka.attributeSelection.GreedyStepwise	[-C, -R]weka.attributeSelection.CfsSubsetEval	[-M, -L]
raw_7yesSC_sum_sz3_it1_os300	weka.classifiers.lazy.LWL	[-K, 60, -A, weka.core.neighboursearch.LinearNNSearch, -W, weka.classifiers.bayes.NaiveBayes, --]null	[]null	[]
raw_7yesSC_sum_sz3_it2_os300	weka.classifiers.trees.RandomForest	[-I, 222, -K, 3, -depth, 0]weka.attributeSelection.GreedyStepwise	[-C, -R]weka.attributeSelection.CfsSubsetEval	[-M, -L]
raw_7yesSC_sum_sz3_it3_os300	weka.classifiers.trees.RandomForest	[-I, 10, -K, 0, -depth, 0]null	[]null	[]
raw_7yesSC_sum_sz1_it1_os300	weka.classifiers.meta.RandomCommittee	[-I, 39, -S, 1, -W, weka.classifiers.trees.RandomTree, --, -M, 1, -K, 0, -depth, 0, -N, 0, -U]null	[]null	[]

raw_7yesSC_sum_sz1_it2_os300	weka.classifiers.lazy.LWL	[-K, 30, -A, weka.core.neighboursearch.LinearNNSearch, -W, weka.classifiers.bayes.NaiveBayesMultinomial, --]null	[]null	[]
raw_7yesSC_sum_sz1_it3_os300	weka.classifiers.meta.AdaBoostM1	[-P, 100, -I, 23, -S, 1, -W, weka.classifiers.trees.RandomForest, --, -I, 5, -K, 0, -depth, 0]null	[]null	[]
raw_7yesSC_sum_sz2_it1_os300	weka.classifiers.lazy.LWL	[-A, weka.core.neighboursearch.LinearNNSearch, -W, weka.classifiers.rules.DecisionTable, --, -E, acc, -S, weka.attributeSelection.BestFirst, -X, 3]weka.attributeSelection.GreedyStepwise	[-C, -B, -R]weka.attributeSelection.CfsSubsetEval	[]
raw_7yesSC_sum_sz2_it2_os300	weka.classifiers.lazy.LWL	[-U, 3, -A, weka.core.neighboursearch.LinearNNSearch, -W, weka.classifiers.rules.DecisionTable, --, -E, acc, -S, weka.attributeSelection.BestFirst, -X, 1]weka.attributeSelection.GreedyStepwise	[-B, -R]weka.attributeSelection.CfsSubsetEval	[-L]
raw_7yesSC_sum_sz2_it3_os300	weka.classifiers.bayes.BayesNet	[-D, -Q, weka.classifiers.bayes.net.search.local.LAGDHillClimber]null	[]null	[]
raw_7yesSC_sum_sz3_it1_os300	weka.classifiers.meta.AdaBoostM1	[-P, 100, -I, 47, -S, 1, -W, weka.classifiers.rules.JRip, --, -N, 1.4473120283879464, -E, -P, -O, 5]weka.attributeSelection.GreedyStepwise	[-C, -B, -R]weka.attributeSelection.CfsSubsetEval	[]
raw_7yesSC_sum_sz3_it2_os300	weka.classifiers.lazy.LWL	[-U, 3, -A, weka.core.neighboursearch.LinearNNSearch, -W, weka.classifiers.rules.DecisionTable, --, -E, acc, -S, weka.attributeSelection.BestFirst, -X, 1]weka.attributeSelection.GreedyStepwise	[-B, -R]weka.attributeSelection.CfsSubsetEval	[-L]
raw_7yesSC_sum_sz3_it3_os300	weka.classifiers.meta.AdaBoostM1	[-P, 54, -I, 7, -S, 1, -W, weka.classifiers.tree	[-C, -B, -R]weka.attributeSelection.CfsSu	[-L]

		s.RandomForest, --, -I, 113, -K, 2, -depth, 0]weka.attributeSelection.GreedyStepwise	bsetEval	
raw_8noSC_sum_sz1_it1_os300	weka.classifiers.meta.RandomSubSpace	[-I, 7, -P, 0.6170521374848286, -S, 1, -W, weka.classifiers.trees.RandomTree, --, -M, 1, -K, 5, -depth, 0, -N, 3]null	[]null	[]
raw_8noSC_sum_sz1_it2_os300	weka.classifiers.meta.AdaBoostM1	[-P, 100, -I, 31, -S, 1, -W, weka.classifiers.trees.J48, --, -O, -A, -M, 2, -C, 0.6673473705136279]null	[]null	[]
raw_8noSC_sum_sz1_it3_os300	weka.classifiers.meta.AdaBoostM1	[-P, 100, -I, 38, -Q, -S, 1, -W, weka.classifiers.trees.RandomForest, --, -I, 2, -K, 9, -depth, 11]null	[]null	[]
raw_8noSC_sum_sz2_it1_os300	weka.classifiers.trees.DecisionStump	[]null	[]null	[]
raw_8noSC_sum_sz2_it2_os300	weka.classifiers.meta.AdaBoostM1	[-P, 100, -I, 74, -S, 1, -W, weka.classifiers.rules.JRip, --, -N, 1.371245273706747, -P, -O, 5]null	[]null	[]
raw_8noSC_sum_sz2_it3_os300	weka.classifiers.lazy.LWL	[-K, 90, -A, weka.core.neighboursearch.LinearNNSearch, -W, weka.classifiers.functions.SimpleLogistic, --, -S, -W, 0]null	[]null	[]
raw_8noSC_sum_sz3_it1_os300	weka.classifiers.lazy.LWL	[-U, 4, -A, weka.core.neighboursearch.LinearNNSearch, -W, weka.classifiers.trees.RandomForest, --, -I, 96, -K, 5, -depth, 18]weka.attributeSelection.GreedyStepwise	[-C, -B, -R]weka.attributeSelection.CfsSubsetEval	[]
raw_8noSC_sum_sz3_it2_os300	weka.classifiers.meta.RandomCommittee	[-I, 27, -S, 1, -W, weka.classifiers.trees.RandomForest, --, -I, 2, -K, 1, -depth, 0]weka.attributeSelection.GreedyStepwise	[-B, -R]weka.attributeSelection.CfsSubsetEval	[]
raw_8noSC_sum_sz3_it3_os300	weka.classifiers.meta.RandomCommittee	[-I, 11, -S, 1, -W, weka.classifiers.trees.RandomForest, --, -I, 89, -K, 2, -depth, 0]null	[]null	[]
raw_8noSC_sum_sz1_it1_os300	weka.classifiers.bayes.BayesNet	[-Q, weka.classifiers.bay	[]null	[]

		es.net.search.local.HillClimber]null		
raw_8noSC_sum_sz1_it2_os300	weka.classifiers.meta.AdaBoostM1	[-P, 92, -I, 20, -S, 1, -W, weka.classifiers.trees.RandomForest, --, -I, 14, -K, 4, -depth, 19]weka.attributeSelection.GreedyStepwise	[-C, -B, -R]weka.attributeSelection.CfsSubsetEval	[-M]
raw_8noSC_sum_sz1_it3_os300	weka.classifiers.lazy.LWL	[-K, 30, -A, weka.core.neighboursearch.LinearNNSearch, -W, weka.classifiers.bayes.NaiveBayes, --, -K]null	[]null	[]
raw_8noSC_sum_sz2_it1_os300	weka.classifiers.meta.AdaBoostM1	[-P, 100, -I, 121, -Q, -S, 1, -W, weka.classifiers.trees.RandomTree, --, -M, 1, -K, 10, -depth, 18, -N, 0]null	[]null	[]
raw_8noSC_sum_sz2_it2_os300	weka.classifiers.meta.RandomCommittee	[-I, 14, -S, 1, -W, weka.classifiers.trees.RandomForest, --, -I, 59, -K, 1, -depth, 0]null	[]null	[]
raw_8noSC_sum_sz2_it3_os300	weka.classifiers.meta.RandomCommittee	[-I, 14, -S, 1, -W, weka.classifiers.trees.RandomForest, --, -I, 59, -K, 1, -depth, 0]null	[]null	[]
raw_8noSC_sum_sz3_it1_os300	weka.classifiers.meta.RandomSubSpace	[-I, 19, -P, 0.8528725896677177, -S, 1, -W, weka.classifiers.trees.RandomTree, --, -M, 1, -K, 8, -depth, 0, -N, 0]null	[]null	[]
raw_8noSC_sum_sz3_it2_os300	weka.classifiers.trees.RandomForest	[-I, 159, -K, 26, -depth, 0]null	[]null	[]
raw_8noSC_sum_sz3_it3_os300	weka.classifiers.meta.AdaBoostM1	[-P, 100, -I, 97, -Q, -S, 1, -W, weka.classifiers.trees.J48, --, -B, -J, -A, -M, 1, -C, 0.8989845224386325]weka.attributeSelection.GreedyStepwise	[-C, -R]weka.attributeSelection.CfsSubsetEval	[-M, -L]
raw_8yesSC_sum_sz1_it1_os300	weka.classifiers.meta.AdaBoostM1	[-P, 100, -I, 107, -Q, -S, 1, -W, weka.classifiers.rules.JRip, --, -N, 2.1528307668676723, -E, -P, -O, 5]weka.attributeSelection.GreedyStepwise	[-R]weka.attributeSelection.CfsSubsetEval	[-L]
raw_8yesSC_sum_sz1_it2_os300	weka.classifiers.lazy.LWL	[-A, weka.core.neighboursearch.LinearNNSearch, -W, weka.classifiers.tree	[-C, -R]weka.attributeSelection.CfsSubsetEval	[-L]

		s.RandomForest, --, -I, 206, -K, 1, -depth, 0]weka.attributeSelection.GreedyStepwise		
raw_8yesSC_sum_sz1_it3_os300	weka.classifiers.meta.AdaBoostM1	[-P, 91, -I, 124, -Q, -S, 1, -W, weka.classifiers.trees.RandomTree, --, -M, 1, -K, 5, -depth, 0, -N, 0, -U]null	[]null	[]
raw_8yesSC_sum_sz2_it1_os300	weka.classifiers.trees.RandomForest	[-I, 222, -K, 3, -depth, 0]weka.attributeSelection.GreedyStepwise	[-C, -R]weka.attributeSelection.CfsSubsetEval	[-M, -L]
raw_8yesSC_sum_sz2_it2_os300	weka.classifiers.meta.AdaBoostM1	[-P, 78, -I, 76, -S, 1, -W, weka.classifiers.trees.J48, --, -O, -B, -J, -A, -S, -M, 3, -C, 0.8586519149476207]null	[]null	[]
raw_8yesSC_sum_sz2_it3_os300	weka.classifiers.lazy.LWL	[-A, weka.core.neighboursearch.LinearNNSearch, -W, weka.classifiers.trees.RandomForest, --, -I, 94, -K, 0, -depth, 0]null	[]null	[]
raw_8yesSC_sum_sz3_it1_os300	weka.classifiers.lazy.LWL	[-A, weka.core.neighboursearch.LinearNNSearch, -W, weka.classifiers.trees.RandomForest, --, -I, 62, -K, 0, -depth, 0]null	[]null	[]
raw_8yesSC_sum_sz3_it2_os300	weka.classifiers.meta.RandomCommittee	[-I, 4, -S, 1, -W, weka.classifiers.trees.RandomForest, --, -I, 27, -K, 0, -depth, 16]weka.attributeSelection.GreedyStepwise	[-C, -B, -R]weka.attributeSelection.CfsSubsetEval	[]
raw_8yesSC_sum_sz3_it3_os300	weka.classifiers.trees.RandomForest	[-I, 10, -K, 0, -depth, 0]null	[]null	[]
raw_8yesSC_sum_sz1_it1_os300	weka.classifiers.trees.RandomForest	[-I, 164, -K, 0, -depth, 19]null	[]null	[]
raw_8yesSC_sum_sz1_it2_os300	weka.classifiers.bayes.BayesNet	[-D, -Q, weka.classifiers.bayes.net.search.local.HillClimber]null	[]null	[]
raw_8yesSC_sum_sz1_it3_os300	weka.classifiers.lazy.KStar	[-B, 71, -M, n]null	[]null	[]
raw_8yesSC_sum_sz2_it1_os300	weka.classifiers.trees.RandomForest	[-I, 10, -K, 0, -depth, 0]null	[]null	[]
raw_8yesSC_sum_sz2_it2_os300	weka.classifiers.meta.RandomSubSpace	[-I, 48, -P, 0.7040799629891291, -S, 1, -W, weka.classifiers.bay	[]null	[]

		es.BayesNet, --, -D, -Q, weka.classifiers.bayes.net.search.local.K2]null		
raw_8yesSC_sum_sz2_it3_os300	weka.classifiers.lazy.LWL	[-U, 2, -A, weka.core.neighboursearch.LinearNNSearch, -W, weka.classifiers.rules.DecisionTable, --, -E, acc, -S, weka.attributeSelection.GreedyStepwise, -X, 4]null	[]null	[]
raw_8yesSC_sum_sz3_it1_os300	weka.classifiers.meta.RandomCommittee	[-I, 5, -S, 1, -W, weka.classifiers.trees.RandomForest, --, -I, 107, -K, 0, -depth, 0]null	[]null	[]
raw_8yesSC_sum_sz3_it2_os300	weka.classifiers.lazy.LWL	[-U, 4, -A, weka.core.neighboursearch.LinearNNSearch, -W, weka.classifiers.trees.RandomForest, --, -I, 21, -K, 15, -depth, 0]weka.attributeSelection.GreedyStepwise	[-C, -R]weka.attributeSelection.CfsSubsetEval	[-M]
raw_8yesSC_sum_sz3_it3_os300	weka.classifiers.meta.AdaBoostM1	[-P, 100, -I, 34, -S, 1, -W, weka.classifiers.rules.JRip, --, -N, 2.383565848120746, -E, -P, -O, 3]null	[]null	[]
stand_7noSC_sum_sz1_it1_os200	weka.classifiers.lazy.LWL	[-U, 4, -A, weka.core.neighboursearch.LinearNNSearch, -W, weka.classifiers.trees.RandomForest, --, -I, 96, -K, 5, -depth, 18]weka.attributeSelection.GreedyStepwise	[-C, -B, -R]weka.attributeSelection.CfsSubsetEval	[]
stand_7noSC_sum_sz1_it2_os200	weka.classifiers.lazy.LWL	[-K, 90, -A, weka.core.neighboursearch.LinearNNSearch, -W, weka.classifiers.functions.SimpleLogistic, --, -S, -W, 0]null	[]null	[]
stand_7noSC_sum_sz1_it3_os200	weka.classifiers.meta.AdaBoostM1	[-P, 100, -I, 83, -Q, -S, 1, -W, weka.classifiers.trees.J48, --, -B, -J, -A, -M, 4, -C, 0.35683272170421754]weka.attributeSelection.GreedyStepwise	[-R]weka.attributeSelection.CfsSubsetEval	[-L]
stand_7noSC_sum_sz1_it1_os200	weka.classifiers.lazy.LWL	[-K, 60, -A, weka.core.neighboursearch.LinearNN	[]null	[]

		Search, -W, weka.classifiers.functions.SMO, --, -C, 1.4117245004356938, -N, 0, -M, -K, weka.classifiers.functions.supportVector.RBFKernel -G 4.7027808370998683E-4]null		
stand_7noSC_sum_sz1_it2_os200	weka.classifiers.meta.AdaBoostM1	[-P, 89, -I, 58, -S, 1, -W, weka.classifiers.rules.DecisionTable, --, -E, auc, -I, -S, weka.attributeSelection.GreedyStepwise, -X, 1]null	[]null	[]
stand_7noSC_sum_sz1_it3_os200	weka.classifiers.lazy.LWL	[-A, weka.core.neighboursearch.LinearNNSearch, -W, weka.classifiers.lazy.IBk, --, -E, -K, 1, -X, -I]null	[]null	[]
stand_7yesSC_sum_sz1_it1_os200	weka.classifiers.lazy.LWL	[-U, 4, -A, weka.core.neighboursearch.LinearNNSearch, -W, weka.classifiers.trees.RandomForest, --, -I, 96, -K, 5, -depth, 18]weka.attributeSelection.GreedyStepwise	[-C, -B, -R]weka.attributeSelection.CfsSubsetEval	[]
stand_7yesSC_sum_sz1_it2_os200	weka.classifiers.trees.RandomForest	[-I, 73, -K, 13, -depth, 0]weka.attributeSelection.GreedyStepwise	[-C, -B, -R]weka.attributeSelection.CfsSubsetEval	[-M, -L]
stand_7yesSC_sum_sz1_it3_os200	weka.classifiers.meta.AdaBoostM1	[-P, 100, -I, 102, -S, 1, -W, weka.classifiers.trees.LMT, --, -B, -P, -M, 10, -W, 0.634535780489995]null	[]null	[]
stand_7yesSC_sum_sz1_it1_os200	weka.classifiers.meta.AdaBoostM1	[-P, 99, -I, 81, -S, 1, -W, weka.classifiers.trees.RandomForest, --, -I, 8, -K, 0, -depth, 13]null	[]null	[]
stand_7yesSC_sum_sz1_it2_os200	weka.classifiers.lazy.LWL	[-K, 120, -A, weka.core.neighboursearch.LinearNNSearch, -W, weka.classifiers.bayes.BayesNet, --, -D, -Q, weka.classifiers.bayes.net.search.local.TabuSearch]null	[]null	[]
stand_7yesSC_sum_sz1_it3_os200	weka.classifiers.lazy.KStar	[-B, 48, -M, d]null	[]null	[]
stand_8noSC_sum_sz1_it1	weka.classifiers.	[-P, 100, -I, 69, -Q, -	[-C, -	[-L]

_os200	meta.AdaBoostM1	S, 1, -W, weka.classifiers.rules.PART, --, -N, 5, -M, 1, -R]weka.attributeSelection.GreedyStepwise	R]weka.attributeSelection.CfsSubsetEval	
stand_8noSC_sum_sz1_it2_os200	weka.classifiers.meta.RandomSubSpace	[-I, 55, -P, 0.6011996656344638, -S, 1, -W, weka.classifiers.rules.JRip, --, -N, 2.3451689246763268, -P, -O, 5]null	[]null	[]
stand_8noSC_sum_sz1_it3_os200	weka.classifiers.lazy.LWL	[-K, 90, -A, weka.core.neighboursearch.LinearNNSearch, -W, weka.classifiers.functions.SimpleLogistic, --, -W, 0]null	[]null	[]
stand_8noSC_sum_sz1_it1_os200	weka.classifiers.lazy.LWL	[-K, 30, -A, weka.core.neighboursearch.LinearNNSearch, -W, weka.classifiers.functions.Logistic, --, -R, 0.0847257114324799]weka.attributeSelection.GreedyStepwise	[-B, -R]weka.attributeSelection.CfsSubsetEval	[-M]
stand_8noSC_sum_sz1_it2_os200	weka.classifiers.lazy.LWL	[-K, 120, -A, weka.core.neighboursearch.LinearNNSearch, -W, weka.classifiers.bayes.NaiveBayes, --, -D]null	[]null	[]
stand_8noSC_sum_sz1_it3_os200	weka.classifiers.lazy.LWL	[-K, 90, -A, weka.core.neighboursearch.LinearNNSearch, -W, weka.classifiers.bayes.NaiveBayes, --]null	[]null	[]
stand_8yesSC_sum_sz1_it1_os200	weka.classifiers.functions.SMO	[-C, 0.9141776780213309, -N, 2, -K, weka.classifiers.functions.supportVector.RBFKernel -G 0.32871887110328457]null	[]null	[]
stand_8yesSC_sum_sz1_it2_os200	weka.classifiers.meta.AdaBoostM1	[-P, 100, -I, 97, -S, 1, -W, weka.classifiers.bayes.BayesNet, --, -Q, weka.classifiers.bayes.net.search.local.HillClimber]null	[]null	[]
stand_8yesSC_sum_sz1_it3_os200	weka.classifiers.meta.AdaBoostM1	[-P, 100, -I, 58, -Q, -S, 1, -W, weka.classifiers.functions.MultilayerPerceptron, --, -L,	[]null	[]

		0.30618692691985017, -M, 0.40509040940243113, -B, -H, a, -C, -S, 1]null		
stand_8yesSC_sum_sz1_it1_os200	weka.classifiers.meta.AdaBoostM1	[-P, 100, -I, 89, -Q, -S, 1, -W, weka.classifiers.rules.JRip, --, -N, 2.9442594394518893, -E, -O, 3]null	[]null	[]
stand_8yesSC_sum_sz1_it2_os200	weka.classifiers.meta.AdaBoostM1	[-P, 100, -I, 27, -S, 1, -W, weka.classifiers.bayes.BayesNet, --, -D, -Q, weka.classifiers.bayes.net.search.local.TAN]weka.attributeSelection.GreedyStepwise	[-C, -B, -R]weka.attributeSelection.CfsSubsetEval	[-M, -L]
stand_8yesSC_sum_sz1_it3_os200	weka.classifiers.meta.AdaBoostM1	[-P, 100, -I, 56, -S, 1, -W, weka.classifiers.rules.JRip, --, -N, 3.827920376224205, -P, -O, 5]null	[]null	[]
stand_7noSC_sum_sz1_it1_os300	weka.classifiers.meta.RandomSubSpace	[-I, 19, -P, 0.8528725896677177, -S, 1, -W, weka.classifiers.trees.RandomTree, --, -M, 1, -K, 8, -depth, 0, -N, 0]null	[]null	[]
stand_7noSC_sum_sz1_it2_os300	weka.classifiers.trees.RandomForest	[-I, 10, -K, 0, -depth, 0]null	[]null	[]
stand_7noSC_sum_sz1_it3_os300	weka.classifiers.trees.RandomForest	[-I, 10, -K, 0, -depth, 0]null	[]null	[]
stand_7noSC_sum_sz1_it1_os300	weka.classifiers.lazy.IBk	[-K, 2]null	[]null	[]
stand_7noSC_sum_sz1_it2_os300	weka.classifiers.meta.AdaBoostM1	[-P, 100, -I, 34, -S, 1, -W, weka.classifiers.rules.JRip, --, -N, 2.383565848120746, -E, -P, -O, 3]null	[]null	[]
stand_7noSC_sum_sz1_it3_os300	weka.classifiers.lazy.IBk	[-E, -K, 31, -X, -I]null	[]null	[]
stand_7yesSC_sum_sz1_it1_os300	weka.classifiers.lazy.LWL	[-U, 4, -A, weka.core.neighboursearch.LinearNNSearch, -W, weka.classifiers.trees.RandomForest, --, -I, 96, -K, 5, -depth, 18]weka.attributeSelection.GreedyStepwise	[-C, -B, -R]weka.attributeSelection.CfsSubsetEval	[]
stand_7yesSC_sum_sz1_it2_os300	weka.classifiers.meta.RandomCommittee	[-I, 23, -S, 1, -W, weka.classifiers.trees.RandomForest, --, -I, 33, -K, 5, -depth, 16]null	[]null	[]
stand_7yesSC_sum_sz1_it	weka.classifiers.	[-P, 100, -I, 113, -S,	[-C, -	[-M, -L]

3_os300	meta.AdaBoostM1	1, -W, weka.classifiers.rules.JRip, --, -N, 4.026382550819031, -E, -O, 5]weka.attributeSelection.GreedyStepwise	R]weka.attributeSelection.CfsSubsetEval	
stand_7yesSC_sum_sz1_it1_os300	weka.classifiers.meta.AdaBoostM1	[-P, 100, -I, 127, -Q, -S, 1, -W, weka.classifiers.trees.LMT, --, -C, -M, 10, -W, 0, -A]null	[]null	[]
stand_7yesSC_sum_sz1_it2_os300	weka.classifiers.lazy.LWL	[-K, 60, -A, weka.core.neighboursearch.LinearNNSearch, -W, weka.classifiers.bayes.BayesNet, --, -D, -Q, weka.classifiers.bayes.net.search.local.TabuSearch]null	[]null	[]
stand_7yesSC_sum_sz1_it3_os300	weka.classifiers.lazy.IBk	[-K, 2]null	[]null	[]
stand_8noSC_sum_sz1_it1_os300	weka.classifiers.meta.AdaBoostM1	[-P, 100, -I, 43, -Q, -S, 1, -W, weka.classifiers.trees.RandomTree, --, -M, 1, -K, 0, -depth, 0, -N, 4, -U]weka.attributeSelection.GreedyStepwise	[-R]weka.attributeSelection.CfsSubsetEval	[-M]
stand_8noSC_sum_sz1_it2_os300	weka.classifiers.meta.AdaBoostM1	[-P, 66, -I, 109, -Q, -S, 1, -W, weka.classifiers.rules.PART, --, -N, 4, -M, 4, -R]weka.attributeSelection.GreedyStepwise	[-C, -R]weka.attributeSelection.CfsSubsetEval	[-M]
stand_8noSC_sum_sz1_it3_os300	weka.classifiers.lazy.LWL	[-K, -1, -A, weka.core.neighboursearch.LinearNNSearch, -W, weka.classifiers.functions.SMO, --, -C, 1.0872214757796854, -N, 2, -K, weka.classifiers.functions.supportVector.Puk -S 4.924370015191547 -O 0.4251591742579399]weka.attributeSelection.GreedyStepwise	[-R]weka.attributeSelection.CfsSubsetEval	[-L]
stand_8noSC_sum_sz1_it1_os300	weka.classifiers.lazy.IBk	[-K, 2]null	[]null	[]
stand_8noSC_sum_sz1_it2_os300	weka.classifiers.bayes.BayesNet	[-D, -Q, weka.classifiers.bayes.net.search.local.LAGDHillClimber]null	[]null	[]

stand_8noSC_sum_sz1_it3_os300	weka.classifiers.bayes.BayesNet	[-D, -Q, weka.classifiers.bayes.net.search.local.HillClimber]null	[]null	[]
stand_8yesSC_sum_sz1_it1_os300	weka.classifiers.meta.AdaBoostM1	[-P, 100, -I, 28, -S, 1, -W, weka.classifiers.trees.LMT, --, -B, -M, 12, -W, 0, -A]weka.attributeSelection.GreedyStepwise	[-B, -R]weka.attributeSelection.CfsSubsetEval	[-L]
stand_8yesSC_sum_sz1_it2_os300	weka.classifiers.lazy.LWL	[-A, weka.core.neighboursearch.LinearNNSearch, -W, weka.classifiers.trees.RandomForest, --, -I, 24, -K, 0, -depth, 0]weka.attributeSelection.GreedyStepwise	[-R]weka.attributeSelection.CfsSubsetEval	[-M]
stand_8yesSC_sum_sz1_it3_os300	weka.classifiers.functions.SMO	[-C, 0.9141776780213309, -N, 2, -K, weka.classifiers.functions.supportVector.RBFKernel -G 0.32871887110328457]null	[]null	[]
stand_8yesSC_sum_sz1_it1_os300	weka.classifiers.meta.AdaBoostM1	[-P, 100, -I, 55, -Q, -S, 1, -W, weka.classifiers.rules.PART, --, -N, 4, -M, 1, -R, -B]weka.attributeSelection.GreedyStepwise	[-C, -R]weka.attributeSelection.CfsSubsetEval	[-M, -L]
stand_8yesSC_sum_sz1_it2_os300	weka.classifiers.lazy.LWL	[-K, 120, -A, weka.core.neighboursearch.LinearNNSearch, -W, weka.classifiers.bayes.BayesNet, --, -D, -Q, weka.classifiers.bayes.net.search.local.TAN]null	[]null	[]
stand_8yesSC_sum_sz1_it3_os300	weka.classifiers.lazy.IBk	[-K, 2]null	[]null	[]
stand_7noSC_sum_sz1_it1_os300	weka.classifiers.trees.RandomForest	[-I, 15, -K, 0, -depth, 0]null	[]null	[]
stand_7noSC_sum_sz1_it2_os300	weka.classifiers.meta.RandomCommittee	[-I, 10, -S, 1, -W, weka.classifiers.trees.RandomForest, --, -I, 13, -K, 0, -depth, 0]null	[]null	[]
stand_7noSC_sum_sz1_it3_os300	weka.classifiers.meta.AdaBoostM1	[-P, 86, -I, 109, -S, 1, -W, weka.classifiers.trees.RandomForest, --, -I, 6, -K, 2, -depth, 6]weka.attributeSelection.GreedyStepwise	[-C, -B, -R]weka.attributeSelection.CfsSubsetEval	[-L]

stand_7noSC_sum_sz2_it1_os300	weka.classifiers.lazy.LWL	[-K, 120, -A, weka.core.neighboursearch.LinearNNSearch, -W, weka.classifiers.functions.SimpleLogistic, --, -S, -W, 0]weka.attributeSelection.GreedyStepwise	[-C, -B, -R]weka.attributeSelection.CfsSubsetEval	[]
stand_7noSC_sum_sz2_it2_os300	weka.classifiers.meta.RandomCommittee	[-I, 3, -S, 1, -W, weka.classifiers.trees.RandomForest, --, -I, 30, -K, 1, -depth, 17]weka.attributeSelection.GreedyStepwise	[-C, -B, -R]weka.attributeSelection.CfsSubsetEval	[]
stand_7noSC_sum_sz2_it3_os300	weka.classifiers.lazy.LWL	[-K, 120, -A, weka.core.neighboursearch.LinearNNSearch, -W, weka.classifiers.functions.SimpleLogistic, --, -W, 0]weka.attributeSelection.GreedyStepwise	[-R]weka.attributeSelection.CfsSubsetEval	[]
stand_7noSC_sum_sz3_it1_os300	weka.classifiers.meta.AdaBoostM1	[-P, 92, -I, 17, -Q, -S, 1, -W, weka.classifiers.trees.RandomForest, --, -I, 7, -K, 6, -depth, 18]null	[]null	[]
stand_7noSC_sum_sz3_it2_os300	weka.classifiers.meta.AdaBoostM1	[-P, 100, -I, 58, -S, 1, -W, weka.classifiers.trees.J48, --, -O, -U, -J, -A, -M, 5]weka.attributeSelection.GreedyStepwise	[-C, -B, -R]weka.attributeSelection.CfsSubsetEval	[-M, -L]
stand_7noSC_sum_sz3_it3_os300	weka.classifiers.meta.AdaBoostM1	[-P, 100, -I, 32, -S, 1, -W, weka.classifiers.trees.RandomTree, --, -M, 2, -K, 14, -depth, 15, -N, 0, -U]weka.attributeSelection.GreedyStepwise	[-C, -B, -R]weka.attributeSelection.CfsSubsetEval	[-L]
stand_7noSC_sum_sz1_it1_os300	weka.classifiers.meta.AdaBoostM1	[-P, 100, -I, 45, -Q, -S, 1, -W, weka.classifiers.trees.RandomForest, --, -I, 18, -K, 7, -depth, 8]weka.attributeSelection.GreedyStepwise	[-C, -R]weka.attributeSelection.CfsSubsetEval	[-M, -L]
stand_7noSC_sum_sz1_it2_os300	weka.classifiers.meta.AdaBoostM1	[-P, 92, -I, 14, -Q, -S, 1, -W, weka.classifiers.rules.JRip, --, -N, 4.06143648931253, -E, -P, -O, 2]null	[]null	[]
stand_7noSC_sum_sz1_it3_os300	weka.classifiers.meta.AdaBoostM	[-P, 100, -I, 14, -Q, -S, 1, -W,	[-B, -R]weka.attribut	[]

	1	weka.classifiers.trees.RandomForest, --, -I, 87, -K, 1, -depth, 0]weka.attributeSelection.GreedyStepwise	eSelection.CfsSubsetEval	
stand_7noSC_sum_sz2_it1_os300	weka.classifiers.meta.AdaBoostM1	[-P, 100, -I, 56, -S, 1, -W, weka.classifiers.trees.RandomForest, --, -I, 2, -K, 4, -depth, 15]weka.attributeSelection.GreedyStepwise	[-R]weka.attributeSelection.CfsSubsetEval	[-M, -L]
stand_7noSC_sum_sz2_it2_os300	weka.classifiers.lazy.LWL	[-A, weka.core.neighboursearch.LinearNNSearch, -W, weka.classifiers.trees.RandomForest, --, -I, 36, -K, 1, -depth, 0]null	[]null	[]
stand_7noSC_sum_sz2_it3_os300	weka.classifiers.meta.AdaBoostM1	[-P, 78, -I, 76, -S, 1, -W, weka.classifiers.trees.J48, --, -O, -B, -J, -A, -S, -M, 3, -C, 0.8586519149476207]null	[]null	[]
stand_7noSC_sum_sz3_it1_os300	weka.classifiers.meta.AdaBoostM1	[-P, 100, -I, 90, -S, 1, -W, weka.classifiers.rules.DecisionTable, --, -E, auc, -I, -S, weka.attributeSelection.BestFirst, -X, 1]null	[]null	[]
stand_7noSC_sum_sz3_it2_os300	weka.classifiers.trees.RandomForest	[-I, 159, -K, 26, -depth, 0]null	[]null	[]
stand_7noSC_sum_sz3_it3_os300	weka.classifiers.lazy.KStar	[-B, 8, -M, d]weka.attributeSelection.GreedyStepwise	[-C, -B, -R]weka.attributeSelection.CfsSubsetEval	[-L]
stand_7yesSC_sum_sz1_it1_os300	weka.classifiers.trees.RandomForest	[-I, 174, -K, 0, -depth, 0]null	[]null	[]
stand_7yesSC_sum_sz1_it2_os300	weka.classifiers.lazy.LWL	[-U, 4, -A, weka.core.neighboursearch.LinearNNSearch, -W, weka.classifiers.functions.SMO, --, -C, 0.9699718404837689, -N, 1, -M, -K, weka.classifiers.functions.supportVector.RBFKernel -G 0.44586540581522544]null	[]null	[]
stand_7yesSC_sum_sz1_it3_os300	weka.classifiers.lazy.LWL	[-K, -1, -A, weka.core.neighboursearch.LinearNNSearch, -W, weka.classifiers.functions.SMO, --, -C,	[]null	[]

		1.4581913116339082, -N, 1, -M, -K, weka.classifiers.functions.supportVector.Puk -S 4.274287666007148 -O 0.8405262369366149]null		
stand_7yesSC_sum_sz2_it1_os300	weka.classifiers.meta.AdaBoostM1	[-P, 100, -I, 16, -Q, -S, 1, -W, weka.classifiers.trees.RandomForest, --, -I, 29, -K, 2, -depth, 19]null	[]null	[]
stand_7yesSC_sum_sz2_it2_os300	weka.classifiers.meta.AdaBoostM1	[-P, 70, -I, 60, -Q, -S, 1, -W, weka.classifiers.trees.RandomForest, --, -I, 8, -K, 0, -depth, 0]weka.attributeSelection.GreedyStepwise	[-C, -B, -R]weka.attributeSelection.CfsSubsetEval	[-M]
stand_7yesSC_sum_sz2_it3_os300	weka.classifiers.trees.RandomForest	[-I, 37, -K, 0, -depth, 17]weka.attributeSelection.GreedyStepwise	[-C, -B, -R]weka.attributeSelection.CfsSubsetEval	[-M, -L]
stand_7yesSC_sum_sz3_it1_os300	weka.classifiers.trees.RandomForest	[-I, 10, -K, 0, -depth, 0]null	[]null	[]
stand_7yesSC_sum_sz3_it2_os300	weka.classifiers.meta.RandomCommittee	[-I, 11, -S, 1, -W, weka.classifiers.trees.RandomForest, --, -I, 130, -K, 1, -depth, 0]null	[]null	[]
stand_7yesSC_sum_sz3_it3_os300	weka.classifiers.lazy.LWL	[-A, weka.core.neighboursearch.LinearNNSearch, -W, weka.classifiers.trees.RandomForest, --, -I, 110, -K, 0, -depth, 0]null	[]null	[]
stand_7yesSC_sum_sz1_it1_os300	weka.classifiers.lazy.IBk	[-K, 1, -I]null	[]null	[]
stand_7yesSC_sum_sz1_it2_os300	weka.classifiers.lazy.LWL	[-K, 120, -A, weka.core.neighboursearch.LinearNNSearch, -W, weka.classifiers.bayes.BayesNet, --, -D, -Q, weka.classifiers.bayes.net.search.local.TabuSearch]null	[]null	[]
stand_7yesSC_sum_sz1_it3_os300	weka.classifiers.meta.AdaBoostM1	[-P, 93, -I, 91, -S, 1, -W, weka.classifiers.trees.J48, --, -O, -J, -S, -M, 1, -C, 0.26480960543806775]weka.attributeSelection.GreedyStepwise	[-C, -B, -R]weka.attributeSelection.CfsSubsetEval	[-M, -L]
stand_7yesSC_sum_sz2_it1_os300	weka.classifiers.trees.RandomFores	[-I, 10, -K, 0, -depth, 0]null	[]null	[]

	t			
stand_7yesSC_sum_sz2_it2_os300	weka.classifiers.lazy.KStar	[-B, 47, -M, d]null	[]null	[]
stand_7yesSC_sum_sz2_it3_os300	weka.classifiers.lazy.LWL	[-U, 1, -A, weka.core.neighboursearch.LinearNNSearch, -W, weka.classifiers.rules.DecisionTable, --, -E, acc, -S, weka.attributeSelection.BestFirst, -X, 4]null	[]null	[]
stand_7yesSC_sum_sz3_it1_os300	weka.classifiers.meta.AdaBoostM1	[-P, 84, -I, 33, -Q, -S, 1, -W, weka.classifiers.rules.JRip, --, -N, 2.892019663927048, -P, -O, 1]null	[]null	[]
stand_7yesSC_sum_sz3_it2_os300	weka.classifiers.meta.Vote	[-R, PROD, -S, 1, -B, weka.classifiers.lazy.KStar -B 43 -M m, -B, weka.classifiers.functions.SimpleLogistic -S -W 0.9238405636892598, -B, weka.classifiers.rules.DecisionTable -E rmse -S weka.attributeSelection.BestFirst -X 4, -B, weka.classifiers.rules.DecisionTable -E acc -I -S weka.attributeSelection.BestFirst -X 1]null	[]null	[]
stand_7yesSC_sum_sz3_it3_os300	weka.classifiers.meta.AdaBoostM1	[-P, 65, -I, 102, -Q, -S, 1, -W, weka.classifiers.lazy.KStar, --, -B, 37, -E, -M, a]null	[]null	[]
stand_8noSC_sum_sz1_it1_os300	weka.classifiers.meta.AdaBoostM1	[-P, 100, -I, 83, -Q, -S, 1, -W, weka.classifiers.trees.J48, --, -B, -J, -A, -M, 4, -C, 0.35683272170421754]weka.attributeSelection.GreedyStepwise	[-R]weka.attributeSelection.CfsSubsetEval	[-L]
stand_8noSC_sum_sz1_it2_os300	weka.classifiers.meta.RandomCommittee	[-I, 31, -S, 1, -W, weka.classifiers.trees.RandomForest, --, -I, 2, -K, 0, -depth, 20]weka.attributeSelection.GreedyStepwise	[-B, -R]weka.attributeSelection.CfsSubsetEval	[]
stand_8noSC_sum_sz1_it3_os300	weka.classifiers.meta.AdaBoostM1	[-P, 100, -I, 64, -S, 1, -W, weka.classifiers.trees.RandomForest, --, -I, 4, -K, 0, -depth,	[]null	[]

		15]null		
stand_8noSC_sum_sz2_it1_os300	weka.classifiers.meta.AdaBoostM1	[-P, 100, -I, 83, -Q, -S, 1, -W, weka.classifiers.trees.J48, --, -B, -J, -A, -M, 4, -C, 0.35683272170421754]weka.attributeSelection.GreedyStepwise	[-R]weka.attributeSelection.CfsSubsetEval	[-L]
stand_8noSC_sum_sz2_it2_os300	weka.classifiers.functions.SMO	[-C, 0.8834901573983901, -N, 1, -K, weka.classifiers.functions.supportVector.Puk -S 2.70071737141395 -O 0.7960004287380918]weka.attributeSelection.GreedyStepwise	[-B, -R]weka.attributeSelection.CfsSubsetEval	[-M]
stand_8noSC_sum_sz2_it3_os300	weka.classifiers.lazy.LWL	[-K, 120, -A, weka.core.neighboursearch.LinearNNSearch, -W, weka.classifiers.functions.MultilayerPerceptron, --, -L, 0.6089914865349235, -M, 0.2820170175598906, -H, o, -C, -S, 1]weka.attributeSelection.GreedyStepwise	[-R]weka.attributeSelection.CfsSubsetEval	[-M]
stand_8noSC_sum_sz3_it1_os300	weka.classifiers.trees.RandomForest	[-I, 10, -K, 0, -depth, 0]null	[]null	[]
stand_8noSC_sum_sz3_it2_os300	weka.classifiers.lazy.LWL	[-U, 4, -A, weka.core.neighboursearch.LinearNNSearch, -W, weka.classifiers.functions.SMO, --, -C, 1.4296431262588447, -N, 1, -K, weka.classifiers.functions.supportVector.Puk -S 3.828431509993488 -O 0.8291772469375045]null	[]null	[]
stand_8noSC_sum_sz3_it3_os300	weka.classifiers.meta.AdaBoostM1	[-P, 100, -I, 58, -Q, -S, 1, -W, weka.classifiers.trees.LMT, --, -C, -P, -M, 3, -W, 0]null	[]null	[]
stand_8noSC_sum_sz1_it1_os300	weka.classifiers.lazy.LWL	[-K, 30, -A, weka.core.neighboursearch.LinearNNSearch, -W, weka.classifiers.functions.SMO, --, -C, 1.308877018768083,	[]null	[]

		-N, 0, -M, -K, weka.classifiers.functions.supportVector.RBFKernel -G 2.753477577494676E-4]null		
stand_8noSC_sum_sz1_it2_os300	weka.classifiers.bayes.BayesNet	[-Q, weka.classifiers.bayes.net.search.local.HillClimber]null	[]null	[]
stand_8noSC_sum_sz1_it3_os300	weka.classifiers.lazy.IBk	[-K, 2, -X, -I]null	[]null	[]
stand_8noSC_sum_sz2_it1_os300	weka.classifiers.lazy.LWL	[-A, weka.core.neighboursearch.LinearNNSearch, -W, weka.classifiers.rules.DecisionTable, --, -E, acc, -S, weka.attributeSelection.BestFirst, -X, 3]weka.attributeSelection.GreedyStepwise	[-C, -B, -R]weka.attributeSelection.CfsSubsetEval	[]
stand_8noSC_sum_sz2_it2_os300	weka.classifiers.meta.AdaBoostM1	[-P, 100, -I, 69, -Q, -S, 1, -W, weka.classifiers.rules.PART, --, -N, 5, -M, 1, -R]weka.attributeSelection.GreedyStepwise	[-C, -R]weka.attributeSelection.CfsSubsetEval	[-L]
stand_8noSC_sum_sz2_it3_os300	weka.classifiers.meta.AdaBoostM1	[-P, 100, -I, 59, -Q, -S, 1, -W, weka.classifiers.trees.J48, --, -O, -S, -M, 1, -C, 0.1758643273465581]null	[]null	[]
stand_8noSC_sum_sz3_it1_os300	weka.classifiers.meta.RandomSubSpace	[-I, 64, -P, 0.16454835529912074, -S, 1, -W, weka.classifiers.trees.RandomForest, --, -I, 7, -K, 28, -depth, 19]null	[]null	[]
stand_8noSC_sum_sz3_it2_os300	weka.classifiers.lazy.KStar	[-B, 55, -M, a]null	[]null	[]
stand_8noSC_sum_sz3_it3_os300	weka.classifiers.meta.AdaBoostM1	[-P, 100, -I, 51, -S, 1, -W, weka.classifiers.bayes.BayesNet, --, -D, -Q, weka.classifiers.bayes.net.search.local.TAN]null	[]null	[]
stand_8yesSC_sum_sz1_it1_os300	weka.classifiers.trees.RandomForest	[-I, 10, -K, 0, -depth, 0]null	[]null	[]
stand_8yesSC_sum_sz1_it2_os300	weka.classifiers.meta.AdaBoostM1	[-P, 100, -I, 18, -Q, -S, 1, -W, weka.classifiers.trees.RandomForest, --, -I, 25, -K, 2, -depth, 0]null	[]null	[]
stand_8yesSC_sum_sz1_it	weka.classifiers.f	[-C,	[]null	[]

3_os300	unctions.SMO	0.9141776780213309, -N, 2, -K, weka.classifiers.functions.supportVector.RBFKernel -G 0.32871887110328457]null		
stand_8yesSC_sum_sz2_it1_os300	weka.classifiers.meta.RandomCommittee	[-I, 4, -S, 1, -W, weka.classifiers.trees.RandomForest, --, -I, 10, -K, 2, -depth, 16]null	[]null	[]
stand_8yesSC_sum_sz2_it2_os300	weka.classifiers.lazy.LWL	[-A, weka.core.neighboursearch.LinearNNSearch, -W, weka.classifiers.trees.RandomForest, --, -I, 36, -K, 1, -depth, 0]null	[]null	[]
stand_8yesSC_sum_sz2_it3_os300	weka.classifiers.meta.AdaBoostM1	[-P, 100, -I, 85, -Q, -S, 1, -W, weka.classifiers.functions.SMO, --, -C, 0.9373109712491259, -N, 2, -K, weka.classifiers.functions.supportVector.Puk -S 2.5890586683260794 -O 0.7339471840994586]weka.attributeSelection.GreedyStepwise	[-B, -R]weka.attributeSelection.CfsSubsetEval	[-M, -L]
stand_8yesSC_sum_sz3_it1_os300	weka.classifiers.trees.RandomForest	[-I, 10, -K, 0, -depth, 0]null	[]null	[]
stand_8yesSC_sum_sz3_it2_os300	weka.classifiers.meta.Bagging	[-P, 100, -I, 73, -S, 1, -W, weka.classifiers.trees.RandomTree, --, -M, 1, -K, 0, -depth, 0, -N, 0, -U]null	[]null	[]
stand_8yesSC_sum_sz3_it3_os300	weka.classifiers.meta.AdaBoostM1	[-P, 100, -I, 7, -S, 1, -W, weka.classifiers.functions.SMO, --, -C, 0.618708993274995, -N, 1, -K, weka.classifiers.functions.supportVector.Puk -S 3.0758368231338262 -O 0.6334685907800855]weka.attributeSelection.GreedyStepwise	[-R]weka.attributeSelection.CfsSubsetEval	[-M, -L]
stand_8yesSC_sum_sz1_it1_os300	weka.classifiers.trees.RandomForest	[-I, 10, -K, 0, -depth, 0]null	[]null	[]
stand_8yesSC_sum_sz1_it2_os300	weka.classifiers.meta.RandomSubSpace	[-I, 48, -P, 0.7040799629891291, -S, 1, -W, weka.classifiers.bay	[]null	[]

		es.BayesNet, --, -D, -Q, weka.classifiers.bayes.net.search.local.K2]null		
stand_8yesSC_sum_sz1_it3_os300	weka.classifiers.lazy.LWL	[-K, 60, -A, weka.core.neighboursearch.LinearNNSearch, -W, weka.classifiers.bayes.NaiveBayes, --, -D]weka.attributeSelection.GreedyStepwise	[-C, -R]weka.attributeSelection.CfsSubsetEval	[-M, -L]
stand_8yesSC_sum_sz2_it1_os300	weka.classifiers.meta.RandomCommittee	[-I, 23, -S, 1, -W, weka.classifiers.trees.RandomTree, --, -M, 2, -K, 4, -depth, 19, -N, 0]null	[]null	[]
stand_8yesSC_sum_sz2_it2_os300	weka.classifiers.lazy.LWL	[-K, -1, -A, weka.core.neighboursearch.LinearNNSearch, -W, weka.classifiers.trees.RandomForest, --, -I, 92, -K, 0, -depth, 0]null	[]null	[]
stand_8yesSC_sum_sz2_it3_os300	weka.classifiers.lazy.KStar	[-B, 79, -M, n]null	[]null	[]
stand_8yesSC_sum_sz3_it1_os300	weka.classifiers.trees.RandomForest	[-I, 10, -K, 0, -depth, 0]null	[]null	[]
stand_8yesSC_sum_sz3_it2_os300	weka.classifiers.meta.AdaBoostM1	[-P, 100, -I, 17, -S, 1, -W, weka.classifiers.trees.RandomForest, --, -I, 5, -K, 10, -depth, 13]null	[]null	[]
stand_8yesSC_sum_sz3_it3_os300	weka.classifiers.lazy.KStar	[-B, 91, -M, a]null	[]null	[]

The RCC column describes the conditions used to build the corresponding RCCs. Below is a summary scheme of the names in the RCC column:.

{norm/stand/raw}_{7/8}{no/yes}SC_{con/sum}_sz{1/2/3}_it{1/2/3}(_over{200/300}).

The fields are separated by an underscore. The first field {stand/norm/raw} specifies if the RCC were standardize, normalized or taken without further modification; The digits of the second field {7/8} indicates the distance in Angstroms used to build the contact map; {no/yes}SC inform if side-chain atoms were used or not in the construction of the contact map. The third field {con/sum} describe if concatenation or addition of each RCC for a protein-protein pair was implemented. From the fourth to the sixth field is the information of the undersampling procedure used, the sz{1/2/3} refers to the proportion of the undersampling of the majority class (positive) with reference to the length of the minority class (negative), as follows: sz1 = undersampling 1:1, sz2 = undersampling 2:1 and sz3 = undersampling 3:1 of positives (P) vs negatives (N) respectively; the fifth field it{1/2/3} refers to the random iteration seed used for the undersampling. Last, the sixth field (_over{200/300}) may or not be present, it is the indicative of the oversampling sets, which all part from an undersampling 1:1 set. In this way, the negative class was synthetically oversampled to generate the same quantity of negative instances 1:2 (P:N) (over200), or twice the quantity of negative instances 1:3 (P:N) (over300).

Table 3. Hyper parameters for best models obtained with AutoWeka using samplings of training sets without redundancy.

RCC	ClassifierName	Arguments	AttributeSearch	Attribute SearchArg	AttributeEval	Attribute EvalArgs
norm_7noSC_con_tra_sz1_it1.outTestStats	weka.classifiers.lazy.LWL	[-K, 30, -A, weka.core.neighboursearch.LinearNNSearch, -W, weka.classifiers.functions.Logistic, --, -R, 5.364192804065933]	null	[]	null	[]
norm_7noSC_con_tra_sz1_it1_over200.outTestStats	weka.classifiers.lazy.KStar	[-B, 23, -M, a]	null	[]	null	[]
norm_7noSC_con_tra_sz1_it1_over300.outTestStats	weka.classifiers.lazy.IBk	[-E, -K, 28, -X, -F]	null	[]	null	[]
norm_7noSC_con_tra_sz1_it2.outTestStats	weka.classifiers.lazy.IBk	[-K, 2, -X, -F]	weka.attributeSelection.GreedyStepwise	[-B, -R]	weka.attributeSelection.CfsSubsetEval	[-M]
norm_7noSC_con_tra_sz1_it2_over200.outTestStats	weka.classifiers.meta.AdaBoostM1	[-P, 100, -I, 119, -S, 1, -W, weka.classifiers.trees.J48, --, -O, -B, -J, -S, -M, 48, -C, 0.6814364759118827]	null	[]	null	[]
norm_7noSC_con_tra_sz1_it2_over300.outTestStats	weka.classifiers.lazy.LWL	[-K, 60, -A, weka.core.neighboursearch.LinearNNSearch, -W, weka.classifiers.functions.MultilayerPerceptron, --, -L, 0.1785572545829176, -M, 0.49930333273052374, -H, t, -C, -S, 1]	weka.attributeSelection.GreedyStepwise	[-C, -R]	weka.attributeSelection.CfsSubsetEval	[]
norm_7noSC_con_tra_sz1_it3.outTestStats	weka.classifiers.lazy.KStar	[-B, 71, -M, n]	null	[]	null	[]
norm_7noSC_con_tra_sz1_it3_over200.outTestStats	weka.classifiers.lazy.IBk	[-E, -K, 8, -X]	null	[]	null	[]
norm_7noSC_con_tra_sz1_it3_over300.outTestStats	weka.classifiers.lazy.IBk	[-K, 2]	null	[]	null	[]
norm_7noSC_con_tra_sz2_it1.outTestStats	weka.classifiers.meta.RandomSubSpace	[-I, 9, -P, 0.5825368043205884, -S, 1, -W, weka.classifiers.lazy.KStar, --, -B, 11, -M, m]	null	[]	null	[]
norm_7noSC_con_tra_sz2_it2.outTestStats	weka.classifiers.meta.AdaBoostM1	[-P, 100, -I, 33, -S, 1, -W, weka.classifiers.trees.RandomForest, --, -I, 13, -K, 0, -depth, 7]	null	[]	null	[]
norm_7noSC_con_tra_sz2_it3.outTestStats	weka.classifiers.meta.AdaBoostM1	[-P, 100, -I, 55, -S, 1, -W, weka.classifiers.bayes.BayesNet, --, -D, -Q, weka.classifiers.bayes.net.search.local.LAGDHillClimber]	null	[]	null	[]
norm_7noSC_con_tra_sz3_it1.outTestStats	weka.classifiers.lazy.KStar	[-B, 26, -M, d]	null	[]	null	[]

norm_7noSC_con_tra_sz3_it2.outTestStats	weka.classifiers.meta.AdaBoostM1	[-P, 100, -I, 36, -S, 1, -W, weka.classifiers.bayes.BayesNet, --, -Q, weka.classifiers.bayes.net.search.local.K2]	null	[]	null	[]
norm_7noSC_con_tra_sz3_it3.outTestStats	weka.classifiers.meta.RandomSubSpace	[-I, 34, -P, 0.21648763830536832, -S, 1, -W, weka.classifiers.trees.J48, --, -U, -B, -J, -A, -M, 1]	null	[]	null	[]
norm_7noSC_sum_tra_sz1_it1.outTestStats	weka.classifiers.meta.AdaBoostM1	[-P, 100, -I, 64, -S, 1, -W, weka.classifiers.trees.LMT, --, -B, -R, -P, -M, 1, -W, 0]	null	[]	null	[]
norm_7noSC_sum_tra_sz1_it1_over200.outTestStats	weka.classifiers.meta.AdaBoostM1	[-P, 100, -I, 19, -S, 1, -W, weka.classifiers.trees.J48, --, -O, -B, -M, 1]	null	[]	null	[]
norm_7noSC_sum_tra_sz1_it1_over300.outTestStats	weka.classifiers.meta.RandomCommittee	[-I, 36, -S, 1, -W, weka.classifiers.trees.RandomForest, --, -I, 5, -K, 0, -depth, 0]	weka.attributeSelection.GreedyStepwise	[-B, -R]	weka.attributeSelection.CfsSubsetEval	[]
norm_7noSC_sum_tra_sz1_it2.outTestStats	weka.classifiers.lazy.LWL	[-U, 1, -A, weka.core.neighboursearch.LinearNNSearch, -W, weka.classifiers.functions.SMO, --, -C, 1.376271748154589, -N, 0, -M, -K, weka.classifiers.functions.supportVector.Puk -S 0.3024853148573099 -O 0.6878560663513378]	null	[]	null	[]
norm_7noSC_sum_tra_sz1_it2_over200.outTestStats	weka.classifiers.meta.AdaBoostM1	[-P, 100, -I, 54, -S, 1, -W, weka.classifiers.trees.J48, --, -O, -B, -A, -M, 30, -C, 0.9365510044019448]	null	[]	null	[]
norm_7noSC_sum_tra_sz1_it2_over300.outTestStats	weka.classifiers.meta.AdaBoostM1	[-P, 100, -I, 17, -Q, -S, 1, -W, weka.classifiers.trees.RandomForest, --, -I, 21, -K, 3, -depth, 8]	null	[]	null	[]
norm_7noSC_sum_tra_sz1_it3.outTestStats	weka.classifiers.meta.AdaBoostM1	[-P, 100, -I, 64, -S, 1, -W, weka.classifiers.trees.LMT, --, -B, -R, -P, -M, 1, -W, 0]	null	[]	null	[]
norm_7noSC_sum_tra_sz1_it3_over200.outTestStats	weka.classifiers.meta.AdaBoostM1	[-P, 100, -I, 109, -Q, -S, 1, -W, weka.classifiers.trees.LMT, --, -B, -M, 1, -W, 0.34497374145084814]	weka.attributeSelection.BestFirst	[-D, 0, -N, 5]	weka.attributeSelection.CfsSubsetEval	[-L]
norm_7noSC_sum_tra_sz1_it3_over300.outTestStats	weka.classifiers.meta.RandomCommittee	[-I, 49, -S, 1, -W, weka.classifiers.trees.RandomForest, --, -I, 253, -K, 0, -depth, 0]	null	[]	null	[]
norm_7noSC_sum_tra_sz2_it1.outTestStats	weka.classifiers.lazy.LWL	[-K, 90, -A, weka.core.neighbours	null	[]	null	[]

		earch.LinearNNSearch, -W, weka.classifiers.functions.SimpleLogistic, --, -S, -W, 0.14991094330775467]				
norm_7noSC_sum_tra_sz2_it2.outTestStats	weka.classifiers.meta.AdaBoostM1	[-P, 100, -I, 64, -S, 1, -W, weka.classifiers.trees.LMT, --, -B, -R, -P, -M, 1, -W, 0]	null	[]	null	[]
norm_7noSC_sum_tra_sz2_it3.outTestStats	weka.classifiers.meta.AdaBoostM1	[-P, 100, -I, 103, -Q, -S, 1, -W, weka.classifiers.trees.RandomTree, --, -M, 13, -K, 12, -depth, 0, -N, 4]	weka.attributeSelection.GreedyStepwise	[-B, -R]	weka.attributeSelection.CfsSubsetEval	[]
norm_7noSC_sum_tra_sz3_it1.outTestStats	weka.classifiers.lazy.LWL	[-K, 60, -A, weka.core.neighboursearch.LinearNNSearch, -W, weka.classifiers.bayes.NaiveBayes, --]	null	[]	null	[]
norm_7noSC_sum_tra_sz3_it2.outTestStats	weka.classifiers.meta.Bagging	[-P, 79, -I, 79, -S, 1, -W, weka.classifiers.trees.LMT, --, -B, -R, -M, 23, -W, 0, -A]	null	[]	null	[]
norm_7noSC_sum_tra_sz3_it3.outTestStats	weka.classifiers.meta.AdaBoostM1	[-P, 100, -I, 74, -Q, -S, 1, -W, weka.classifiers.trees.RandomForest, --, -I, 25, -K, 26, -depth, 12]	weka.attributeSelection.GreedyStepwise	[-B, -R]	weka.attributeSelection.CfsSubsetEval	[-M]
norm_7yesSC_con_tra_sz1_it1.outTestStats	weka.classifiers.lazy.LWL	[-K, 90, -A, weka.core.neighboursearch.LinearNNSearch, -W, weka.classifiers.bayes.BayesNet, --, -D, -Q, weka.classifiers.bayes.net.search.local.K2]	weka.attributeSelection.GreedyStepwise	[-R]	weka.attributeSelection.CfsSubsetEval	[]
norm_7yesSC_con_tra_sz1_it1_over200.outTestStats	weka.classifiers.lazy.LWL	[-A, weka.core.neighboursearch.LinearNNSearch, -W, weka.classifiers.lazy.IBk, --, -K, 3, -X, -I]	null	[]	null	[]
norm_7yesSC_con_tra_sz1_it1_over300.outTestStats	weka.classifiers.lazy.LWL	[-K, 30, -A, weka.core.neighboursearch.LinearNNSearch, -W, weka.classifiers.functions.MultilayerPerceptron, --, -L, 0.39657670633870534, -M, 0.4187285609661533, -H, a, -S, 1]	weka.attributeSelection.GreedyStepwise	[-C, -B, -R]	weka.attributeSelection.CfsSubsetEval	[-L]
norm_7yesSC_con_tra_sz1_it2.outTestStats	weka.classifiers.meta.RandomCommittee	[-I, 38, -S, 1, -W, weka.classifiers.trees.RandomTree, --, -M, 5, -K, 0, -depth, 0, -N, 0, -U]	null	[]	null	[]
norm_7yesSC_con_tra_sz1_it2_over200.outTestStats	weka.classifiers.lazy.LWL	[-K, 90, -A, weka.core.neighboursearch.LinearNNSearc	null	[]	null	[]

		h, -W, weka.classifiers.bayes.BayesNet, --, -D, -Q, weka.classifiers.bayes.net.search.local.K2]				
norm_7yesSC_con_tra_sz1_it2_over300.outTestStats	weka.classifiers.bayes.BayesNet	[-D, -Q, weka.classifiers.bayes.net.search.local.K2]	null	[]	null	[]
norm_7yesSC_con_tra_sz1_it3.outTestStats	weka.classifiers.lazy.KStar	[-B, 76, -M, d]	weka.attributeSelection.GreedyStepwise	[-C, -B, -R]	weka.attributeSelection.CfsSubsetEval	[-M]
norm_7yesSC_con_tra_sz1_it3_over200.outTestStats	weka.classifiers.meta.Vote	[-R, MAX, -S, 1, -B, weka.classifiers.lazy.KStar -B 49 -M m, -B, weka.classifiers.trees.LMT -B -R -C -M 1 -W 0.2316048408449548, -B, weka.classifiers.rules.JRip -N 4.97832459259406 -E -O 1, -B, weka.classifiers.rules.DecisionTable -E rmse -I -S weka.attributeSelection.BestFirst -X 3]	weka.attributeSelection.GreedyStepwise	[-B, -R]	weka.attributeSelection.CfsSubsetEval	[-M, -L]
norm_7yesSC_con_tra_sz1_it3_over300.outTestStats	weka.classifiers.lazy.IBk	[-E, -K, 1, -X, -I]	null	[]	null	[]
norm_7yesSC_con_tra_sz2_it1.outTestStats	weka.classifiers.lazy.LWL	[-A, weka.core.neighboursearch.LinearNNSearch, -W, weka.classifiers.bayes.BayesNet, --, -D, -Q, weka.classifiers.bayes.net.search.local.HillClimber]	null	[]	null	[]
norm_7yesSC_con_tra_sz2_it2.outTestStats	weka.classifiers.lazy.LWL	[-U, 3, -A, weka.core.neighboursearch.LinearNNSearch, -W, weka.classifiers.rules.DecisionTable, --, -E, acc, -S, weka.attributeSelection.BestFirst, -X, 1]	weka.attributeSelection.GreedyStepwise	[-B, -R]	weka.attributeSelection.CfsSubsetEval	[-L]
norm_7yesSC_con_tra_sz2_it3.outTestStats	weka.classifiers.meta.Vote	[-R, PROD, -S, 1, -B, weka.classifiers.lazy.KStar -B 43 -M m, -B, weka.classifiers.functions.SimpleLogistic -S -W 0.9238405636892598, -B, weka.classifiers.rules.DecisionTable -E rmse -S weka.attributeSelection.BestFirst -X 4, -B, weka.classifiers.rules.DecisionTable -E acc -I -S weka.attributeSelecti	null	[]	null	[]

		on.BestFirst -X 1]				
norm_7yesSC_con_tra_sz3_it1.outTestStats	weka.classifiers.meta.Vote	[-R, PROD, -S, 1, -B, weka.classifiers.lazy.KStar -B 43 -M m, -B, weka.classifiers.functions.SimpleLogistic -S -W 0.9238405636892598, -B, weka.classifiers.rules.DecisionTable -E rmse -S weka.attributeSelection.BestFirst -X 4, -B, weka.classifiers.rules.DecisionTable -E acc -I -S weka.attributeSelection.BestFirst -X 1]	null	[]	null	[]
norm_7yesSC_con_tra_sz3_it2.outTestStats	weka.classifiers.meta.RandomCommittee	[-I, 33, -S, 1, -W, weka.classifiers.trees.RandomForest, --, -I, 25, -K, 0, -depth, 0]	null	[]	null	[]
norm_7yesSC_con_tra_sz3_it3.outTestStats	weka.classifiers.meta.RandomCommittee	[-I, 19, -S, 1, -W, weka.classifiers.trees.RandomForest, --, -I, 93, -K, 10, -depth, 0]	null	[]	null	[]
norm_7yesSC_sum_tra_sz1_it1.outTestStats	weka.classifiers.trees.RandomForest	[-I, 62, -K, 16, -depth, 15]	null	[]	null	[]
norm_7yesSC_sum_tra_sz1_it1_over200.outTestStats	weka.classifiers.lazy.LWL	[-U, 2, -A, weka.core.neighboursearch.LinearNNSearch, -W, weka.classifiers.trees.RandomForest, --, -I, 247, -K, 0, -depth, 0]	null	[]	null	[]
norm_7yesSC_sum_tra_sz1_it1_over300.outTestStats	weka.classifiers.meta.RandomCommittee	[-I, 10, -S, 1, -W, weka.classifiers.trees.RandomTree, --, -M, 3, -K, 0, -depth, 12, -N, 0, -U]	null	[]	null	[]
norm_7yesSC_sum_tra_sz1_it2.outTestStats	weka.classifiers.meta.RandomCommittee	[-I, 3, -S, 1, -W, weka.classifiers.trees.RandomForest, --, -I, 71, -K, 1, -depth, 0]	null	[]	null	[]
norm_7yesSC_sum_tra_sz1_it2_over200.outTestStats	weka.classifiers.meta.AdaBoostM1	[-P, 100, -I, 24, -Q, -S, 1, -W, weka.classifiers.trees.J48, --, -J, -A, -S, -M, 1, -C, 0.9561584733463078]	null	[]	null	[]
norm_7yesSC_sum_tra_sz1_it2_over300.outTestStats	weka.classifiers.trees.RandomForest	[-I, 45, -K, 0, -depth, 0]	weka.attributeSelection.GreedyStepwise	[-B, -R]	weka.attributeSelection.CfsSubsetEval	[]
norm_7yesSC_sum_tra_sz1_it3.outTestStats	weka.classifiers.meta.RandomSubSpace	[-I, 34, -P, 0.6409896496155877, -S, 1, -W, weka.classifiers.rules.PART, --, -M, 3, -B]	null	[]	null	[]
norm_7yesSC_sum_tra_sz1_it3_over200.outTestStats	weka.classifiers.lazy.LWL	[-A, weka.core.neighboursearch.LinearNNSearch, -W, weka.classifiers.trees.RandomForest, --, -I, 108, -K, 0, -depth, 12]	null	[]	null	[]

norm_7yesSC_sum_tra_sz1_it3_over300.outTestStats	weka.classifiers.meta.RandomSubSpace	[-I, 19, -P, 0.8528725896677177, -S, 1, -W, weka.classifiers.trees.RandomTree, --, -M, 1, -K, 8, -depth, 0, -N, 0]	null	[]	null	[]
norm_7yesSC_sum_tra_sz2_it1.outTestStats	weka.classifiers.meta.AdaBoostM1	[-P, 100, -I, 83, -Q, -S, 1, -W, weka.classifiers.trees.J48, --, -B, -J, -A, -M, 4, -C, 0.35683272170421754]	weka.attributeSelection.GreedyStepwise	[-R]	weka.attributeSelection.CfsSubsetEval	[-L]
norm_7yesSC_sum_tra_sz2_it2.outTestStats	weka.classifiers.meta.AdaBoostM1	[-P, 81, -I, 117, -S, 1, -W, weka.classifiers.trees.J48, --, -O, -J, -S, -M, 1, -C, 0.25299437343177156]	weka.attributeSelection.GreedyStepwise	[-C, -B, -R]	weka.attributeSelection.CfsSubsetEval	[]
norm_7yesSC_sum_tra_sz2_it3.outTestStats	weka.classifiers.meta.AdaBoostM1	[-P, 100, -I, 76, -Q, -S, 1, -W, weka.classifiers.rules.JRip, --, -N, 2.851960831017474, -E, -P, -O, 4]	null	[]	null	[]
norm_7yesSC_sum_tra_sz3_it1.outTestStats	weka.classifiers.trees.RandomForest	[-I, 10, -K, 0, -depth, 0]	null	[]	null	[]
norm_7yesSC_sum_tra_sz3_it2.outTestStats	weka.classifiers.meta.RandomCommittee	[-I, 42, -S, 1, -W, weka.classifiers.trees.RandomTree, --, -M, 1, -K, 2, -depth, 0, -N, 0, -U]	null	[]	null	[]
norm_7yesSC_sum_tra_sz3_it3.outTestStats	weka.classifiers.meta.AdaBoostM1	[-P, 100, -I, 64, -S, 1, -W, weka.classifiers.trees.LMT, --, -B, -R, -P, -M, 1, -W, 0]	null	[]	null	[]
norm_8noSC_con_tra_sz1_it1.outTestStats	weka.classifiers.lazy.KStar	[-B, 48, -M, d]	null	[]	null	[]
norm_8noSC_con_tra_sz1_it1_over200.outTestStats	weka.classifiers.lazy.LWL	[-K, 90, -A, weka.core.neighboursearch.LinearNNSearch, -W, weka.classifiers.functions.MultilayerPerceptron, --, -L, 0.8887372979649986, -M, 0.5949245081853208, -H, t, -R, -S, 1]	null	[]	null	[]
norm_8noSC_con_tra_sz1_it1_over300.outTestStats	weka.classifiers.meta.RandomSubSpace	[-I, 48, -P, 0.7040799629891291, -S, 1, -W, weka.classifiers.bayes.BayesNet, --, -D, -Q, weka.classifiers.bayes.net.search.local.K2]	null	[]	null	[]
norm_8noSC_con_tra_sz1_it2.outTestStats	weka.classifiers.lazy.LWL	[-K, 30, -A, weka.core.neighboursearch.LinearNNSearch, -W, weka.classifiers.bayes.NaiveBayes, --, -D]	null	[]	null	[]
norm_8noSC_con_tra_sz1_it2_over200.outTestStats	weka.classifiers.lazy.LWL	[-A, weka.core.neighboursearch.LinearNNSearc	null	[]	null	[]

		h, -W, weka.classifiers.bayes.BayesNet, --, -D, -Q, weka.classifiers.bayes.net.search.local.K2]			
norm_8noSC_con_tra_sz1_it2_over300.outTestStats	weka.classifiers.lazy.LWL	[-K, 120, -A, weka.core.neighboursearch.LinearNNSearch, -W, weka.classifiers.bayes.NaiveBayes, --, -D]	null	[]	null	[]
norm_8noSC_con_tra_sz1_it3.outTestStats	weka.classifiers.meta.AdaBoostM1	[-P, 100, -I, 25, -Q, -S, 1, -W, weka.classifiers.trees.RandomForest, --, -I, 10, -K, 0, -depth, 0]	weka.attributeSelection.BestFirst	[-D, 2, -N, 6]	weka.attributeSelection.CfsSubsetEval	[-M, -L]
norm_8noSC_con_tra_sz1_it3_over200.outTestStats	weka.classifiers.lazy.KStar	[-B, 55, -M, a]	null	[]	null	[]
norm_8noSC_con_tra_sz1_it3_over300.outTestStats	weka.classifiers.meta.AdaBoostM1	[-P, 100, -I, 109, -Q, -S, 1, -W, weka.classifiers.trees.LMT, --, -B, -M, 1, -W, 0.34497374145084814]	weka.attributeSelection.BestFirst	[-D, 0, -N, 5]	weka.attributeSelection.CfsSubsetEval	[-L]
norm_8noSC_con_tra_sz2_it1.outTestStats	weka.classifiers.meta.RandomCommittee	[-I, 23, -S, 1, -W, weka.classifiers.trees.RandomForest, --, -I, 17, -K, 0, -depth, 0]	null	[]	null	[]
norm_8noSC_con_tra_sz2_it2.outTestStats	weka.classifiers.meta.Vote	[-R, PROD, -S, 1, -B, weka.classifiers.lazy.KStar -B 43 -M m, -B, weka.classifiers.functions.SimpleLogistic -S -W 0.9238405636892598, -B, weka.classifiers.rules.DecisionTable -E rmse -S weka.attributeSelection.BestFirst -X 4, -B, weka.classifiers.rules.DecisionTable -E acc -I -S weka.attributeSelection.BestFirst -X 1]	null	[]	null	[]
norm_8noSC_con_tra_sz2_it3.outTestStats	weka.classifiers.meta.RandomSubSpace	[-I, 52, -P, 0.13143350213818272, -S, 1, -W, weka.classifiers.trees.RandomForest, --, -I, 81, -K, 3, -depth, 0]	null	[]	null	[]
norm_8noSC_con_tra_sz3_it1.outTestStats	weka.classifiers.trees.RandomForest	[-I, 154, -K, 0, -depth, 16]	null	[]	null	[]
norm_8noSC_con_tra_sz3_it2.outTestStats	weka.classifiers.meta.RandomSubSpace	[-I, 36, -P, 0.2685536367337107, -S, 1, -W, weka.classifiers.trees.RandomForest, --, -I, 47, -K, 9, -depth, 17]	weka.attributeSelection.BestFirst	[-D, 0, -N, 7]	weka.attributeSelection.CfsSubsetEval	[-L]
norm_8noSC_con_tra_sz3_it3.outTestStats	weka.classifiers.meta.RandomSubSpace	[-I, 36, -P, 0.2757331204049802, -S, 1, -W, weka.classifiers.lazy.IBk, --, -E, -K, 10, -X]	null	[]	null	[]
norm_8noSC_sum_tra_sz	weka.classifiers.laz	[-A,	null	[]	null	[]

1_it1.outTestStats	y.LWL	weka.core.neighboursearch.LinearNNSearch, -W, weka.classifiers.trees.RandomForest, --, -I, 129, -K, 1, -depth, 0]				
norm_8noSC_sum_tra_sz1_it1_over200.outTestStats	weka.classifiers.meta.AdaBoostM1	[-P, 93, -I, 16, -Q, -S, 1, -W, weka.classifiers.trees.RandomForest, --, -I, 36, -K, 0, -depth, 0]	weka.attributeSelection.GreedyStepwise	[-C, -B, -R]	weka.attributeSelection.CfsSubsetEval	[]
norm_8noSC_sum_tra_sz1_it1_over300.outTestStats	weka.classifiers.meta.AdaBoostM1	[-P, 100, -I, 38, -Q, -S, 1, -W, weka.classifiers.rules.JRip, --, -N, 2.795973676167696, -O, 1]	weka.attributeSelection.GreedyStepwise	[-C, -R]	weka.attributeSelection.CfsSubsetEval	[]
norm_8noSC_sum_tra_sz1_it2.outTestStats	weka.classifiers.lazy.LWL	[-K, 90, -A, weka.core.neighboursearch.LinearNNSearch, -W, weka.classifiers.functions.SimpleLogistic, --, -W, 0]	null	[]	null	[]
norm_8noSC_sum_tra_sz1_it2_over200.outTestStats	weka.classifiers.meta.AdaBoostM1	[-P, 100, -I, 64, -S, 1, -W, weka.classifiers.trees.LMT, --, -B, -R, -P, -M, 1, -W, 0]	null	[]	null	[]
norm_8noSC_sum_tra_sz1_it2_over300.outTestStats	weka.classifiers.lazy.LWL	[-U, 4, -A, weka.core.neighboursearch.LinearNNSearch, -W, weka.classifiers.functions.SMO, --, -C, 1.4296431262588447, -N, 1, -K, weka.classifiers.functions.supportVector.Puk -S 3.828431509993488 -O 0.8291772469375045]	null	[]	null	[]
norm_8noSC_sum_tra_sz1_it3.outTestStats	weka.classifiers.trees.RandomForest	[-I, 58, -K, 5, -depth, 0]	weka.attributeSelection.GreedyStepwise	[-R]	weka.attributeSelection.CfsSubsetEval	[]
norm_8noSC_sum_tra_sz1_it3_over200.outTestStats	weka.classifiers.lazy.LWL	[-K, 90, -A, weka.core.neighboursearch.LinearNNSearch, -W, weka.classifiers.functions.SMO, --, -C, 0.5264944664536044, -N, 2, -M, -K, weka.classifiers.functions.supportVector.PolyKernel -E 3.234474776081758 -L]	weka.attributeSelection.GreedyStepwise	[-R]	weka.attributeSelection.CfsSubsetEval	[-M]
norm_8noSC_sum_tra_sz1_it3_over300.outTestStats	weka.classifiers.meta.AdaBoostM1	[-P, 100, -I, 56, -S, 1, -W, weka.classifiers.rules.JRip, --, -N, 3.827920376224205, -P, -O, 5]	null	[]	null	[]
norm_8noSC_sum_tra_sz2_it1.outTestStats	weka.classifiers.meta.RandomCommit	[-I, 14, -S, 1, -W, weka.classifiers.trees.	null	[]	null	[]

	tee	RandomForest, --, -I, 59, -K, 1, -depth, 0]				
norm_8noSC_sum_tra_sz2_it2.outTestStats	weka.classifiers.lazy.LWL	[-K, 60, -A, weka.core.neighboursearch.LinearNNSearch, -W, weka.classifiers.functions.MultilayerPerceptron, --, -L, 0.7503328129570422, -M, 0.3081129205826309, -B, -H, o, -C, -R, -D, -S, 1]	null	[]	null	[]
norm_8noSC_sum_tra_sz2_it3.outTestStats	weka.classifiers.meta.AdaBoostM1	[-P, 74, -I, 14, -Q, -S, 1, -W, weka.classifiers.trees.RandomForest, --, -I, 70, -K, 0, -depth, 0]	null	[]	null	[]
norm_8noSC_sum_tra_sz3_it1.outTestStats	weka.classifiers.meta.AdaBoostM1	[-P, 100, -I, 110, -S, 1, -W, weka.classifiers.trees.REPTree, --, -M, 12, -V, 0.03182161484820228, -L, 19, -P]	weka.attributeSelection.GreedyStepwise	[-C, -R]	weka.attributeSelection.CfsSubsetEval	[-M]
norm_8noSC_sum_tra_sz3_it2.outTestStats	weka.classifiers.lazy.LWL	[-K, 120, -A, weka.core.neighboursearch.LinearNNSearch, -W, weka.classifiers.functions.SMO, --, -C, 0.9454034718656065, -N, 2, -K, weka.classifiers.functions.supportVector.NormalizedPolyKernel -E 4.352831895347352]	null	[]	null	[]
norm_8noSC_sum_tra_sz3_it3.outTestStats	weka.classifiers.meta.AdaBoostM1	[-P, 100, -I, 83, -Q, -S, 1, -W, weka.classifiers.trees.J48, --, -B, -J, -A, -M, 4, -C, 0.35683272170421754]	weka.attributeSelection.GreedyStepwise	[-R]	weka.attributeSelection.CfsSubsetEval	[-L]
norm_8yesSC_con_tra_sz1_it1.outTestStats	weka.classifiers.meta.AdaBoostM1	[-P, 100, -I, 118, -Q, -S, 1, -W, weka.classifiers.rules.JRip, --, -N, 3.949389960549097, -P, -O, 4]	weka.attributeSelection.GreedyStepwise	[-R]	weka.attributeSelection.CfsSubsetEval	[-M, -L]
norm_8yesSC_con_tra_sz1_it1_over200.outTestStats	weka.classifiers.lazy.IBk	[-E, -K, 1, -X]	null	[]	null	[]
norm_8yesSC_con_tra_sz1_it1_over300.outTestStats	weka.classifiers.lazy.IBk	[-E, -K, 15, -X, -F]	null	[]	null	[]
norm_8yesSC_con_tra_sz1_it2.outTestStats	weka.classifiers.lazy.KStar	[-B, 81, -M, n]	null	[]	null	[]
norm_8yesSC_con_tra_sz1_it2_over200.outTestStats	weka.classifiers.lazy.IBk	[-K, 1, -X, -I]	null	[]	null	[]
norm_8yesSC_con_tra_sz1_it2_over300.outTestStats	weka.classifiers.bayes.BayesNet	[-D, -Q, weka.classifiers.bayes.net.search.local.HillClimber]	null	[]	null	[]

norm_8yesSC_con_tra_sz1_it3.outTestStats	weka.classifiers.meta.RandomCommittee	[-I, 26, -S, 1, -W, weka.classifiers.trees.RandomTree, --, -M, 1, -K, 2, -depth, 17, -N, 0]	null	[]	null	[]
norm_8yesSC_con_tra_sz1_it3_over200.outTestStats	weka.classifiers.lazy.LWL	[-K, 120, -A, weka.core.neighboursearch.LinearNNSearch, -W, weka.classifiers.functions.SimpleLogistic, --, -S, -W, 0]	weka.attributeSelection.GreedyStepwise	[-C, -B, -R]	weka.attributeSelection.CfsSubsetEval	[]
norm_8yesSC_con_tra_sz1_it3_over300.outTestStats	weka.classifiers.lazy.LWL	[-K, 120, -A, weka.core.neighboursearch.LinearNNSearch, -W, weka.classifiers.trees.RandomForest, --, -I, 72, -K, 3, -depth, 19]	weka.attributeSelection.GreedyStepwise	[-B, -R]	weka.attributeSelection.CfsSubsetEval	[-L]
norm_8yesSC_con_tra_sz2_it1.outTestStats	weka.classifiers.lazy.LWL	[-A, weka.core.neighboursearch.LinearNNSearch, -W, weka.classifiers.bayes.BayesNet, --, -Q, weka.classifiers.bayes.net.search.local.K2]	weka.attributeSelection.GreedyStepwise	[-B, -R]	weka.attributeSelection.CfsSubsetEval	[-L]
norm_8yesSC_con_tra_sz2_it2.outTestStats	weka.classifiers.lazy.KStar	[-B, 88, -M, n]	null	[]	null	[]
norm_8yesSC_con_tra_sz2_it3.outTestStats	weka.classifiers.meta.Vote	[-R, PROD, -S, 1, -B, weka.classifiers.lazy.KStar -B 43 -M m, -B, weka.classifiers.functions.SimpleLogistic -S -W 0.9238405636892598, -B, weka.classifiers.rules.DecisionTable -E rmse -S weka.attributeSelection.BestFirst -X 4, -B, weka.classifiers.rules.DecisionTable -E acc -I -S weka.attributeSelection.BestFirst -X 1]	null	[]	null	[]
norm_8yesSC_con_tra_sz3_it1.outTestStats	weka.classifiers.trees.RandomForest	[-I, 159, -K, 26, -depth, 0]	null	[]	null	[]
norm_8yesSC_con_tra_sz3_it2.outTestStats	weka.classifiers.meta.Vote	[-R, PROD, -S, 1, -B, weka.classifiers.lazy.KStar -B 43 -M m, -B, weka.classifiers.functions.SimpleLogistic -S -W 0.9238405636892598, -B, weka.classifiers.rules.DecisionTable -E rmse -S weka.attributeSelection.BestFirst -X 4, -B, weka.classifiers.rules.DecisionTable -E acc -I -S weka.attributeSelection	null	[]	null	[]

		on.BestFirst -X 1]				
norm_8yesSC_con_tra_sz3_it3.outTestStats	weka.classifiers.meta.RandomCommittee	[-I, 8, -S, 1, -W, weka.classifiers.trees.RandomForest, --, -I, 52, -K, 0, -depth, 0]	null	[]	null	[]
norm_8yesSC_sum_tra_sz1_it1.outTestStats	weka.classifiers.meta.AdaBoostM1	[-P, 99, -I, 32, -Q, -S, 1, -W, weka.classifiers.trees.RandomForest, --, -I, 102, -K, 3, -depth, 0]	null	[]	null	[]
norm_8yesSC_sum_tra_sz1_it1_over200.outTestStats	weka.classifiers.meta.RandomCommittee	[-I, 50, -S, 1, -W, weka.classifiers.trees.RandomTree, --, -M, 1, -K, 2, -depth, 0, -N, 0]	null	[]	null	[]
norm_8yesSC_sum_tra_sz1_it1_over300.outTestStats	weka.classifiers.meta.AdaBoostM1	[-P, 100, -I, 83, -Q, -S, 1, -W, weka.classifiers.trees.J48, --, -B, -J, -A, -M, 4, -C, 0.35683272170421754]	weka.attributeSelection.GreedyStepwise	[-R]	weka.attributeSelection.CfsSubsetEval	[-L]
norm_8yesSC_sum_tra_sz1_it2.outTestStats	weka.classifiers.meta.AdaBoostM1	[-P, 100, -I, 28, -S, 1, -W, weka.classifiers.trees.RandomForest, --, -I, 20, -K, 0, -depth, 0]	null	[]	null	[]
norm_8yesSC_sum_tra_sz1_it2_over200.outTestStats	weka.classifiers.meta.RandomCommittee	[-I, 56, -S, 1, -W, weka.classifiers.trees.RandomForest, --, -I, 2, -K, 2, -depth, 0]	null	[]	null	[]
norm_8yesSC_sum_tra_sz1_it2_over300.outTestStats	weka.classifiers.meta.RandomCommittee	[-I, 51, -S, 1, -W, weka.classifiers.trees.RandomTree, --, -M, 4, -K, 0, -depth, 0, -N, 0, -U]	null	[]	null	[]
norm_8yesSC_sum_tra_sz1_it3.outTestStats	weka.classifiers.meta.AdaBoostM1	[-P, 100, -I, 21, -Q, -S, 1, -W, weka.classifiers.trees.RandomForest, --, -I, 2, -K, 0, -depth, 15]	null	[]	null	[]
norm_8yesSC_sum_tra_sz1_it3_over200.outTestStats	weka.classifiers.meta.AdaBoostM1	[-P, 100, -I, 11, -S, 1, -W, weka.classifiers.trees.LMT, --, -B, -C, -M, 1, -W, 0.009659721272996746, -A]	null	[]	null	[]
norm_8yesSC_sum_tra_sz1_it3_over300.outTestStats	weka.classifiers.meta.RandomCommittee	[-I, 59, -S, 1, -W, weka.classifiers.trees.RandomForest, --, -I, 5, -K, 8, -depth, 0]	null	[]	null	[]
norm_8yesSC_sum_tra_sz2_it1.outTestStats	weka.classifiers.meta.AdaBoostM1	[-P, 100, -I, 32, -Q, -S, 1, -W, weka.classifiers.trees.J48, --, -B, -S, -M, 1, -C, 0.6579615394211651]	null	[]	null	[]
norm_8yesSC_sum_tra_sz2_it2.outTestStats	weka.classifiers.meta.AdaBoostM1	[-P, 100, -I, 32, -Q, -S, 1, -W, weka.classifiers.trees.J48, --, -B, -S, -M, 1, -C, 0.6579615394211651]	null	[]	null	[]
norm_8yesSC_sum_tra_sz2_it3.outTestStats	weka.classifiers.meta.AdaBoostM1	[-P, 100, -I, 31, -Q, -S, 1, -W, weka.classifiers.trees.	weka.attributeSelection.GreedyStepwise	[-C, -B, -R]	weka.attributeSelection.CfsSubsetEval	[]

		RandomForest, --, -I, 28, -K, 0, -depth, 0]				
norm_8yesSC_sum_tra_sz3_it1.outTestStats	weka.classifiers.lazy.LWL	[-U, 4, -A, weka.core.neighboursearch.LinearNNSearch, -W, weka.classifiers.trees.RandomForest, --, -I, 96, -K, 5, -depth, 18]	weka.attributeSelection.GreedyStepwise	[-C, -B, -R]	weka.attributeSelection.CfsSubsetEval	[]
norm_8yesSC_sum_tra_sz3_it2.outTestStats	weka.classifiers.meta.RandomCommittee	[-I, 33, -S, 1, -W, weka.classifiers.trees.RandomTree, --, -M, 3, -K, 0, -depth, 0, -N, 0, -U]	null	[]	null	[]
norm_8yesSC_sum_tra_sz3_it3.outTestStats	weka.classifiers.meta.RandomCommittee	[-I, 8, -S, 1, -W, weka.classifiers.trees.RandomForest, --, -I, 104, -K, 2, -depth, 0]	null	[]	null	[]
raw_7noSC_con_tra_sz1_it1.outTestStats	weka.classifiers.meta.AdaBoostM1	[-P, 100, -I, 92, -S, 1, -W, weka.classifiers.rules.JRip, --, -N, 3.7058803931051343, -E, -O, 2]	weka.attributeSelection.GreedyStepwise	[-C, -B, -R]	weka.attributeSelection.CfsSubsetEval	[-M, -L]
raw_7noSC_con_tra_sz1_it1_over200.outTestStats	weka.classifiers.meta.Vote	[-R, MAX, -S, 1, -B, weka.classifiers.lazy.KStar -B 49 -M m, -B, weka.classifiers.trees.LMT -B -R -C -M 1 -W 0.2316048408449548, -B, weka.classifiers.rules.JRip -N 4.97832459259406 -E -O 1, -B, weka.classifiers.rules.DecisionTable -E rmse -I -S weka.attributeSelection.BestFirst -X 3]	weka.attributeSelection.GreedyStepwise	[-B, -R]	weka.attributeSelection.CfsSubsetEval	[-M, -L]
raw_7noSC_con_tra_sz1_it1_over300.outTestStats	weka.classifiers.lazy.IBk	[-K, 2, -X, -F]	weka.attributeSelection.GreedyStepwise	[-B, -R]	weka.attributeSelection.CfsSubsetEval	[-M]
raw_7noSC_con_tra_sz1_it2.outTestStats	weka.classifiers.meta.AdaBoostM1	[-P, 100, -I, 95, -S, 1, -W, weka.classifiers.bayes.BayesNet, --, -D, -Q, weka.classifiers.bayes.net.search.local.HillClimber]	weka.attributeSelection.GreedyStepwise	[-C, -B, -R]	weka.attributeSelection.CfsSubsetEval	[]
raw_7noSC_con_tra_sz1_it2_over200.outTestStats	weka.classifiers.meta.Bagging	[-P, 78, -I, 94, -S, 1, -W, weka.classifiers.lazy.IBk, --, -E, -K, 20, -X, -I]	null	[]	null	[]
raw_7noSC_con_tra_sz1_it2_over300.outTestStats	weka.classifiers.meta.AdaBoostM1	[-P, 100, -I, 50, -S, 1, -W, weka.classifiers.trees.J48, --, -B, -J, -M, 8, -C, 0.8524523812990805]	null	[]	null	[]
raw_7noSC_con_tra_sz1_it3.outTestStats	weka.classifiers.lazy.LWL	[-K, 10, -A, weka.core.neighboursearch.LinearNNSearch, -W, weka.classifiers.functi	weka.attributeSelection.GreedyStepwise	[-C, -R]	weka.attributeSelection.CfsSubsetEval	[-M, -L]

		ons.MultilayerPerceptron, --, -L, 0.38206504040298417, -M, 0.7437153940403738, -B, -H, i, -R, -D, -S, 1]				
raw_7noSC_con_tra_sz1_it3_over200.outTestStats	weka.classifiers.lazy.IBk	[-E, -K, 2, -X]	null	[]	null	[]
raw_7noSC_con_tra_sz1_it3_over300.outTestStats	weka.classifiers.lazy.IBk	[-K, 2]	null	[]	null	[]
raw_7noSC_con_tra_sz2_it1.outTestStats	weka.classifiers.lazy.LWL	[-U, 4, -A, weka.core.neighboursearch.LinearNNSearch, -W, weka.classifiers.trees.RandomForest, --, -I, 125, -K, 0, -depth, 0]	null	[]	null	[]
raw_7noSC_con_tra_sz2_it2.outTestStats	weka.classifiers.meta.AdaBoostM1	[-P, 100, -I, 47, -S, 1, -W, weka.classifiers.trees.J48, --, -O, -U, -B, -A, -M, 6]	null	[]	null	[]
raw_7noSC_con_tra_sz2_it3.outTestStats	weka.classifiers.meta.Bagging	[-P, 85, -I, 72, -S, 1, -W, weka.classifiers.lazy.KStar, --, -B, 51, -M, d]	null	[]	null	[]
raw_7noSC_con_tra_sz3_it1.outTestStats	weka.classifiers.meta.Bagging	[-P, 85, -I, 72, -S, 1, -W, weka.classifiers.lazy.KStar, --, -B, 51, -M, d]	null	[]	null	[]
raw_7noSC_con_tra_sz3_it2.outTestStats	weka.classifiers.trees.RandomForest	[-I, 159, -K, 26, -depth, 0]	null	[]	null	[]
raw_7noSC_con_tra_sz3_it3.outTestStats	weka.classifiers.meta.AdaBoostM1	[-P, 100, -I, 76, -S, 1, -W, weka.classifiers.rules.JRip, --, -N, 2.1302836979847495, -P, -O, 2]	weka.attributeSelection.BestFirst	[-D, 2, -N, 6]	weka.attributeSelection.CfsSubsetEval	[-M, -L]
raw_7noSC_sum_tra_sz1_it1.outTestStats	weka.classifiers.meta.AdaBoostM1	[-P, 85, -I, 78, -Q, -S, 1, -W, weka.classifiers.trees.RandomForest, --, -I, 8, -K, 0, -depth, 0]	null	[]	null	[]
raw_7noSC_sum_tra_sz1_it1_over200.outTestStats	weka.classifiers.meta.RandomCommittee	[-I, 38, -S, 1, -W, weka.classifiers.trees.RandomForest, --, -I, 81, -K, 0, -depth, 0]	null	[]	null	[]
raw_7noSC_sum_tra_sz1_it1_over300.outTestStats	weka.classifiers.trees.RandomForest	[-I, 159, -K, 26, -depth, 0]	null	[]	null	[]
raw_7noSC_sum_tra_sz1_it2.outTestStats	weka.classifiers.meta.AdaBoostM1	[-P, 100, -I, 114, -S, 1, -W, weka.classifiers.rules.JRip, --, -N, 4.178618461495404, -E, -P, -O, 5]	weka.attributeSelection.GreedyStepwise	[-C, -R]	weka.attributeSelection.CfsSubsetEval	[]
raw_7noSC_sum_tra_sz1_it2_over200.outTestStats	weka.classifiers.meta.RandomCommittee	[-I, 37, -S, 1, -W, weka.classifiers.trees.RandomForest, --, -I, 68, -K, 0, -depth, 19]	weka.attributeSelection.GreedyStepwise	[-B, -R]	weka.attributeSelection.CfsSubsetEval	[-L]
raw_7noSC_sum_tra_sz1_it2_over300.outTestStats	weka.classifiers.lazy.LWL	[-K, -1, -A, weka.core.neighboursearch.LinearNNSearch, -W, weka.classifiers.trees.	weka.attributeSelection.GreedyStepwise	[-B, -R]	weka.attributeSelection.CfsSubsetEval	[-L]

		RandomForest, --, -I, 151, -K, 0, -depth, 16]				
raw_7noSC_sum_tra_sz1_it3.outTestStats	weka.classifiers.meta.AdaBoostM1	[-P, 100, -I, 59, -Q, -S, 1, -W, weka.classifiers.rules.PART, --, -N, 3, -M, 3, -R, -B]	weka.attributeSelection.GreedyStepwise	[-B, -R]	weka.attributeSelection.CfsSubsetEval	[-M]
raw_7noSC_sum_tra_sz1_it3_over200.outTestStats	weka.classifiers.meta.AdaBoostM1	[-P, 100, -I, 50, -Q, -S, 1, -W, weka.classifiers.trees.RandomTree, --, -M, 5, -K, 8, -depth, 0, -N, 0]	weka.attributeSelection.GreedyStepwise	[-C, -B, -R]	weka.attributeSelection.CfsSubsetEval	[-M, -L]
raw_7noSC_sum_tra_sz1_it3_over300.outTestStats	weka.classifiers.meta.AdaBoostM1	[-P, 100, -I, 109, -Q, -S, 1, -W, weka.classifiers.trees.LMT, --, -B, -M, 1, -W, 0.34497374145084814]	weka.attributeSelection.BestFirst	[-D, 0, -N, 5]	weka.attributeSelection.CfsSubsetEval	[-L]
raw_7noSC_sum_tra_sz2_it1.outTestStats	weka.classifiers.meta.AdaBoostM1	[-P, 100, -I, 64, -S, 1, -W, weka.classifiers.trees.LMT, --, -B, -R, -P, -M, 1, -W, 0]	null	[]	null	[]
raw_7noSC_sum_tra_sz2_it2.outTestStats	weka.classifiers.meta.AdaBoostM1	[-P, 100, -I, 36, -S, 1, -W, weka.classifiers.trees.RandomForest, --, -I, 4, -K, 1, -depth, 0]	null	[]	null	[]
raw_7noSC_sum_tra_sz2_it3.outTestStats	weka.classifiers.meta.AdaBoostM1	[-P, 100, -I, 35, -Q, -S, 1, -W, weka.classifiers.trees.LMT, --, -B, -P, -M, 20, -W, 0.2496482763476563]	null	[]	null	[]
raw_7noSC_sum_tra_sz3_it1.outTestStats	weka.classifiers.meta.RandomCommittee	[-I, 2, -S, 1, -W, weka.classifiers.trees.RandomForest, --, -I, 206, -K, 2, -depth, 0]	null	[]	null	[]
raw_7noSC_sum_tra_sz3_it2.outTestStats	weka.classifiers.meta.AdaBoostM1	[-P, 100, -I, 76, -Q, -S, 1, -W, weka.classifiers.rules.JRip, --, -N, 2.851960831017474, -E, -P, -O, 4]	null	[]	null	[]
raw_7noSC_sum_tra_sz3_it3.outTestStats	weka.classifiers.meta.AdaBoostM1	[-P, 100, -I, 32, -Q, -S, 1, -W, weka.classifiers.trees.J48, --, -B, -S, -M, 1, -C, 0.6579615394211651]	null	[]	null	[]
raw_7yesSC_con_tra_sz1_it1.outTestStats	weka.classifiers.meta.AdaBoostM1	[-P, 100, -I, 48, -S, 1, -W, weka.classifiers.trees.RandomForest, --, -I, 123, -K, 4, -depth, 8]	weka.attributeSelection.GreedyStepwise	[-C, -B, -R]	weka.attributeSelection.CfsSubsetEval	[]
raw_7yesSC_con_tra_sz1_it1_over200.outTestStats	weka.classifiers.lazy.LWL	[-K, 10, -A, weka.core.neighboursearch.LinearNNSearch, -W, weka.classifiers.bayes.NaiveBayesMultinomial, --]	weka.attributeSelection.GreedyStepwise	[-B, -R]	weka.attributeSelection.CfsSubsetEval	[-L]
raw_7yesSC_con_tra_sz1_it1_over300.outTestStats	weka.classifiers.lazy.LWL	[-K, 30, -A, weka.core.neighboursearch.LinearNNSearch, -W,	weka.attributeSelection.GreedyStepwise	[-C, -R]	weka.attributeSelection.CfsSubsetEval	[-L]

		weka.classifiers.functions.MultilayerPerceptron, --, -L, 0.23192937802342725, -M, 0.7097954183313643, -H, t, -C, -S, 1]				
raw_7yesSC_con_tra_sz1_it2.outTestStats	weka.classifiers.lazy.LWL	[-K, 60, -A, weka.core.neighboursearch.LinearNNSearch, -W, weka.classifiers.bayes.BayesNet, --, -D, -Q, weka.classifiers.bayes.net.search.local.TAN]	null	[]	null	[]
raw_7yesSC_con_tra_sz1_it2_over200.outTestStats	weka.classifiers.lazy.LWL	[-K, 90, -A, weka.core.neighboursearch.LinearNNSearch, -W, weka.classifiers.bayes.NaiveBayesMultinomial, --]	null	[]	null	[]
raw_7yesSC_con_tra_sz1_it2_over300.outTestStats	weka.classifiers.meta.AdaBoostM1	[-P, 100, -I, 32, -Q, -S, 1, -W, weka.classifiers.trees.J48, --, -B, -S, -M, 1, -C, 0.6579615394211651]	null	[]	null	[]
raw_7yesSC_con_tra_sz1_it3.outTestStats	weka.classifiers.lazy.KStar	[-B, 10, -M, a]	null	[]	null	[]
raw_7yesSC_con_tra_sz1_it3_over200.outTestStats	weka.classifiers.lazy.LWL	[-K, 30, -A, weka.core.neighboursearch.LinearNNSearch, -W, weka.classifiers.bayes.NaiveBayesMultinomial, --]	null	[]	null	[]
raw_7yesSC_con_tra_sz1_it3_over300.outTestStats	weka.classifiers.lazy.IBk	[-K, 2, -X, -F]	weka.attributeSelection.GreedyStepwise	[-B, -R]	weka.attributeSelection.CfsSubsetEval	[-M]
raw_7yesSC_con_tra_sz2_it1.outTestStats	weka.classifiers.lazy.LWL	[-U, 3, -A, weka.core.neighboursearch.LinearNNSearch, -W, weka.classifiers.rules.DecisionTable, --, -E, acc, -S, weka.attributeSelection.BestFirst, -X, 1]	weka.attributeSelection.GreedyStepwise	[-B, -R]	weka.attributeSelection.CfsSubsetEval	[-L]
raw_7yesSC_con_tra_sz2_it2.outTestStats	weka.classifiers.meta.RandomSubSpace	[-I, 19, -P, 0.8528725896677177, -S, 1, -W, weka.classifiers.trees.RandomTree, --, -M, 1, -K, 8, -depth, 0, -N, 0]	null	[]	null	[]
raw_7yesSC_con_tra_sz2_it3.outTestStats	weka.classifiers.meta.AdaBoostM1	[-P, 100, -I, 109, -Q, -S, 1, -W, weka.classifiers.trees.LMT, --, -B, -M, 1, -W, 0.34497374145084814]	weka.attributeSelection.BestFirst	[-D, 0, -N, 5]	weka.attributeSelection.CfsSubsetEval	[-L]
raw_7yesSC_con_tra_sz3_it1.outTestStats	weka.classifiers.lazy.KStar	[-B, 48, -M, n]	null	[]	null	[]
raw_7yesSC_con_tra_sz3_it2.outTestStats	weka.classifiers.meta.AdaBoostM1	[-P, 100, -I, 36, -Q, -S, 1, -W,	null	[]	null	[]

		weka.classifiers.trees.RandomForest, --, -I, 8, -K, 0, -depth, 0]			
raw_7yesSC_con_tra_sz3_it3.outTestStats	weka.classifiers.meta.RandomSubSpace	[-I, 36, -P, 0.2757331204049802, -S, 1, -W, weka.classifiers.lazy.IBk, --, -E, -K, 10, -X]	null	[]	null	[]
raw_7yesSC_sum_tra_sz1_it1.outTestStats	weka.classifiers.functions.SMO	[-C, 0.9729812123384092, -N, 1, -M, -K, weka.classifiers.functions.supportVector.Puk -S 1.7985869051211523 -O 0.32566573811921185]	weka.attributeSelection.GreedyStepwise	[-C, -R]	weka.attributeSelection.CfsSubsetEval	[-M, -L]
raw_7yesSC_sum_tra_sz1_it1_over200.outTestStats	weka.classifiers.meta.RandomCommittee	[-I, 7, -S, 1, -W, weka.classifiers.trees.RandomForest, --, -I, 11, -K, 2, -depth, 12]	null	[]	null	[]
raw_7yesSC_sum_tra_sz1_it1_over300.outTestStats	weka.classifiers.meta.RandomCommittee	[-I, 21, -S, 1, -W, weka.classifiers.trees.RandomForest, --, -I, 18, -K, 0, -depth, 18]	null	[]	null	[]
raw_7yesSC_sum_tra_sz1_it2.outTestStats	weka.classifiers.meta.AdaBoostM1	[-P, 87, -I, 3, -S, 1, -W, weka.classifiers.trees.RandomForest, --, -I, 41, -K, 0, -depth, 0]	null	[]	null	[]
raw_7yesSC_sum_tra_sz1_it2_over200.outTestStats	weka.classifiers.lazy.LWL	[-K, 90, -A, weka.core.neighboursearch.LinearNNSearch, -W, weka.classifiers.functions.SMO, --, -C, 1.1158066309948338, -N, 0, -M, -K, weka.classifiers.functions.supportVector.Puk -S 7.988677990770362 -O 0.8938385737937203]	weka.attributeSelection.GreedyStepwise	[-C, -B, -R]	weka.attributeSelection.CfsSubsetEval	[-L]
raw_7yesSC_sum_tra_sz1_it2_over300.outTestStats	weka.classifiers.meta.RandomCommittee	[-I, 7, -S, 1, -W, weka.classifiers.trees.RandomForest, --, -I, 97, -K, 0, -depth, 0]	weka.attributeSelection.GreedyStepwise	[-B, -R]	weka.attributeSelection.CfsSubsetEval	[-M, -L]
raw_7yesSC_sum_tra_sz1_it3.outTestStats	weka.classifiers.meta.RandomCommittee	[-I, 37, -S, 1, -W, weka.classifiers.trees.RandomForest, --, -I, 68, -K, 0, -depth, 19]	weka.attributeSelection.GreedyStepwise	[-B, -R]	weka.attributeSelection.CfsSubsetEval	[-L]
raw_7yesSC_sum_tra_sz1_it3_over200.outTestStats	weka.classifiers.meta.AdaBoostM1	[-P, 100, -I, 30, -S, 1, -W, weka.classifiers.rules.JRip, --, -N, 3.7088387250145973, -P, -O, 1]	null	[]	null	[]
raw_7yesSC_sum_tra_sz1_it3_over300.outTestStats	weka.classifiers.lazy.LWL	[-A, weka.core.neighboursearch.LinearNNSearch, -W, weka.classifiers.trees.RandomForest, --, -I, 28, -K, 0, -depth, 0]	null	[]	null	[]
raw_7yesSC_sum_tra_sz2_it1.outTestStats	weka.classifiers.lazy.LWL	[-K, 60, -A, weka.core.neighboursearch.LinearNNSearc	null	[]	null	[]

		h, -W, weka.classifiers.functions.SMO, --, -C, 1.4117245004356938, -N, 0, -M, -K, weka.classifiers.functions.supportVector.RBFKernel -G 4.7027808370998683E-4]				
raw_7yesSC_sum_tra_sz2_it2.outTestStats	weka.classifiers.meta.AdaBoostM1	[-P, 100, -I, 33, -S, 1, -W, weka.classifiers.trees.RandomForest, --, -I, 13, -K, 0, -depth, 7]	null	[]	null	[]
raw_7yesSC_sum_tra_sz2_it3.outTestStats	weka.classifiers.meta.AdaBoostM1	[-P, 100, -I, 126, -S, 1, -W, weka.classifiers.trees.RandomForest, --, -I, 4, -K, 0, -depth, 0]	null	[]	null	[]
raw_7yesSC_sum_tra_sz3_it1.outTestStats	weka.classifiers.trees.RandomForest	[-I, 222, -K, 3, -depth, 0]	weka.attributeSelection.GreedyStepwise	[-C, -R]	weka.attributeSelection.CfsSubsetEval	[-M, -L]
raw_7yesSC_sum_tra_sz3_it2.outTestStats	weka.classifiers.meta.AdaBoostM1	[-P, 53, -I, 118, -S, 1, -W, weka.classifiers.trees.J48, --, -O, -B, -S, -M, 2]	null	[]	null	[]
raw_7yesSC_sum_tra_sz3_it3.outTestStats	weka.classifiers.meta.RandomSubSpace	[-I, 36, -P, 0.2757331204049802, -S, 1, -W, weka.classifiers.lazy.IBk, --, -E, -K, 10, -X]	null	[]	null	[]
raw_8noSC_con_tra_sz1_it1.outTestStats	weka.classifiers.meta.RandomCommittee	[-I, 12, -S, 1, -W, weka.classifiers.trees.RandomTree, --, -M, 2, -K, 0, -depth, 0, -N, 0, -U]	null	[]	null	[]
raw_8noSC_con_tra_sz1_it1_over200.outTestStats	weka.classifiers.lazy.IBk	[-K, 2]	null	[]	null	[]
raw_8noSC_con_tra_sz1_it1_over300.outTestStats	weka.classifiers.lazy.IBk	[-K, 4, -X]	weka.attributeSelection.GreedyStepwise	[-C, -R]	weka.attributeSelection.CfsSubsetEval	[-M]
raw_8noSC_con_tra_sz1_it2.outTestStats	weka.classifiers.lazy.IBk	[-E, -K, 10, -X]	weka.attributeSelection.GreedyStepwise	[-C, -R]	weka.attributeSelection.CfsSubsetEval	[]
raw_8noSC_con_tra_sz1_it2_over200.outTestStats	weka.classifiers.lazy.IBk	[-E, -K, 8, -X, -F]	null	[]	null	[]
raw_8noSC_con_tra_sz1_it2_over300.outTestStats	weka.classifiers.lazy.IBk	[-K, 2]	null	[]	null	[]
raw_8noSC_con_tra_sz1_it3.outTestStats	weka.classifiers.meta.AdaBoostM1	[-P, 100, -I, 77, -S, 1, -W, weka.classifiers.trees.J48, --, -B, -A, -M, 1]	null	[]	null	[]
raw_8noSC_con_tra_sz1_it3_over200.outTestStats	weka.classifiers.lazy.LWL	[-K, 30, -A, weka.core.neighboursearch.LinearNNSearch, -W, weka.classifiers.functions.SimpleLogistic, --, -S, -W, 0, -A]	null	[]	null	[]
raw_8noSC_con_tra_sz1_it3_over300.outTestStats	weka.classifiers.lazy.LWL	[-K, 10, -A, weka.core.neighboursearch.LinearNNSearch, -W, weka.classifiers.functi	weka.attributeSelection.GreedyStepwise	[-R]	weka.attributeSelection.CfsSubsetEval	[]

		ons.MultilayerPerceptron, --, -L, 0.2462377889875508, -M, 0.34874414345313476, -B, -H, a, -R, -S, 1]			
raw_8noSC_con_tra_sz2_it1.outTestStats	weka.classifiers.meta.RandomCommittee	[-I, 3, -S, 1, -W, weka.classifiers.trees.RandomForest, --, -I, 30, -K, 11, -depth, 0]	null	[]	null	[]
raw_8noSC_con_tra_sz2_it2.outTestStats	weka.classifiers.lazy.LWL	[-K, 30, -A, weka.core.neighboursearch.LinearNNSearch, -W, weka.classifiers.trees.RandomForest, --, -I, 36, -K, 0, -depth, 0]	weka.attributeSelection.GreedyStepwise	[-B, -R]	weka.attributeSelection.CfsSubsetEval	[]
raw_8noSC_con_tra_sz2_it3.outTestStats	weka.classifiers.lazy.LWL	[-U, 3, -A, weka.core.neighboursearch.LinearNNSearch, -W, weka.classifiers.trees.RandomForest, --, -I, 32, -K, 0, -depth, 18]	weka.attributeSelection.GreedyStepwise	[-B, -R]	weka.attributeSelection.CfsSubsetEval	[-M]
raw_8noSC_con_tra_sz3_it1.outTestStats	weka.classifiers.trees.RandomForest	[-I, 159, -K, 26, -depth, 0]	null	[]	null	[]
raw_8noSC_con_tra_sz3_it2.outTestStats	weka.classifiers.lazy.KStar	[-B, 53, -M, d]	weka.attributeSelection.GreedyStepwise	[-B, -R]	weka.attributeSelection.CfsSubsetEval	[]
raw_8noSC_con_tra_sz3_it3.outTestStats	weka.classifiers.meta.RandomSubSpace	[-I, 26, -P, 0.12747956987991974, -S, 1, -W, weka.classifiers.lazy.KStar, --, -B, 3, -M, a]	weka.attributeSelection.GreedyStepwise	[-C, -B, -R]	weka.attributeSelection.CfsSubsetEval	[-M, -L]
raw_8noSC_sum_tra_sz1_it1.outTestStats	weka.classifiers.meta.AdaBoostM1	[-P, 100, -I, 83, -S, 1, -W, weka.classifiers.trees.RandomForest, --, -I, 6, -K, 0, -depth, 18]	weka.attributeSelection.GreedyStepwise	[-C, -B, -R]	weka.attributeSelection.CfsSubsetEval	[-L]
raw_8noSC_sum_tra_sz1_it1_over200.outTestStats	weka.classifiers.meta.AdaBoostM1	[-P, 100, -I, 74, -S, 1, -W, weka.classifiers.trees.J48, --, -U, -J, -M, 3]	weka.attributeSelection.GreedyStepwise	[-C, -N, 15]	weka.attributeSelection.CfsSubsetEval	[-M]
raw_8noSC_sum_tra_sz1_it1_over300.outTestStats	weka.classifiers.meta.AttributeSelectedClassifier	[-S, weka.attributeSelection.GreedyStepwise, -E, weka.attributeSelection.CfsSubsetEval, -W, weka.classifiers.trees.RandomForest, --, -I, 54, -K, 0, -depth, 0]	weka.attributeSelection.GreedyStepwise	[-N, 815]	weka.attributeSelection.CfsSubsetEval	[-M]
raw_8noSC_sum_tra_sz1_it2.outTestStats	weka.classifiers.meta.AdaBoostM1	[-P, 100, -I, 76, -Q, -S, 1, -W, weka.classifiers.rules.JRip, --, -N, 2.851960831017474, -E, -P, -O, 4]	null	[]	null	[]
raw_8noSC_sum_tra_sz1_it2_over200.outTestStats	weka.classifiers.lazy.LWL	[-K, 90, -A, weka.core.neighboursearch.LinearNNSearch, -W, weka.classifiers.functions.MultilayerPerceptron, --, -L,	null	[]	null	[]

		0.3174172575276706, -M, 0.5159658708637493, -H, i, -C, -R, -S, 1]				
raw_8noSC_sum_tra_sz1_it2_over300.outTestStats	weka.classifiers.trees.RandomForest	[-I, 90, -K, 6, -depth, 0]	weka.attributeSelection.BestFirst	[-D, 0, -N, 3]	weka.attributeSelection.CfsSubsetEval	[]
raw_8noSC_sum_tra_sz1_it3.outTestStats	weka.classifiers.lazy.LWL	[-K, 60, -A, weka.core.neighboursearch.LinearNNSearch, -W, weka.classifiers.functions.MultilayerPerceptron, --, -L, 0.582352122519684, -M, 0.5365649567733705, -H, a, -C, -S, 1]	null	[]	null	[]
raw_8noSC_sum_tra_sz1_it3_over200.outTestStats	weka.classifiers.meta.AdaBoostM1	[-P, 100, -I, 116, -S, 1, -W, weka.classifiers.trees.RandomTree, --, -M, 37, -K, 7, -depth, 8, -N, 0]	weka.attributeSelection.GreedyStepwise	[-C, -B, -R]	weka.attributeSelection.CfsSubsetEval	[-L]
raw_8noSC_sum_tra_sz1_it3_over300.outTestStats	weka.classifiers.meta.AdaBoostM1	[-P, 100, -I, 11, -S, 1, -W, weka.classifiers.trees.LMT, --, -B, -C, -M, 1, -W, 0.009659721272996746, -A]	null	[]	null	[]
raw_8noSC_sum_tra_sz2_it1.outTestStats	weka.classifiers.lazy.LWL	[-K, 90, -A, weka.core.neighboursearch.LinearNNSearch, -W, weka.classifiers.bayes.NaiveBayes, --]	null	[]	null	[]
raw_8noSC_sum_tra_sz2_it2.outTestStats	weka.classifiers.trees.RandomForest	[-I, 222, -K, 3, -depth, 0]	weka.attributeSelection.GreedyStepwise	[-C, -R]	weka.attributeSelection.CfsSubsetEval	[-M, -L]
raw_8noSC_sum_tra_sz2_it3.outTestStats	weka.classifiers.meta.RandomCommittee	[-I, 14, -S, 1, -W, weka.classifiers.trees.RandomForest, --, -I, 59, -K, 1, -depth, 0]	null	[]	null	[]
raw_8noSC_sum_tra_sz3_it1.outTestStats	weka.classifiers.meta.RandomSubSpace	[-I, 19, -P, 0.8528725896677177, -S, 1, -W, weka.classifiers.trees.RandomTree, --, -M, 1, -K, 8, -depth, 0, -N, 0]	null	[]	null	[]
raw_8noSC_sum_tra_sz3_it2.outTestStats	weka.classifiers.meta.AdaBoostM1	[-P, 100, -I, 99, -S, 1, -W, weka.classifiers.rules.JRip, --, -N, 1.227101281182847, -E, -P, -O, 4]	null	[]	null	[]
raw_8noSC_sum_tra_sz3_it3.outTestStats	weka.classifiers.meta.RandomSubSpace	[-I, 36, -P, 0.2757331204049802, -S, 1, -W, weka.classifiers.lazy.IBk, --, -E, -K, 10, -X]	null	[]	null	[]
raw_8yesSC_con_tra_sz1_it1.outTestStats	weka.classifiers.meta.Vote	[-R, MAX, -S, 1, -B, weka.classifiers.lazy.KStar -B 2 -M n]	null	[]	null	[]
raw_8yesSC_con_tra_sz1	weka.classifiers.laz	[-K, 30, -A,	null	[]	null	[]

_it1_over200.outTestStats	y.LWL	weka.core.neighboursearch.LinearNNSearch, -W, weka.classifiers.functions.Logistic, --, -R, 7.680961862819534E-6]				
raw_8yesSC_con_tra_sz1_it1_over300.outTestStats	weka.classifiers.lazy.IBk	[-E, -K, 2, -I]	null	[]	null	[]
raw_8yesSC_con_tra_sz1_it2.outTestStats	weka.classifiers.meta.Vote	[-R, MAX, -S, 1, -B, weka.classifiers.lazy.KStar -B 49 -M m, -B, weka.classifiers.trees.LMT -B -R -C -M 1 -W 0.2316048408449548, -B, weka.classifiers.rules.JRip -N 4.97832459259406 -E -O 1, -B, weka.classifiers.rules.DecisionTable -E rmse -I -S weka.attributeSelection.BestFirst -X 3]	weka.attributeSelection.GreedyStepwise	[-B, -R]	weka.attributeSelection.CfsSubsetEval	[-M, -L]
raw_8yesSC_con_tra_sz1_it2_over200.outTestStats	weka.classifiers.lazy.KStar	[-B, 55, -M, a]	null	[]	null	[]
raw_8yesSC_con_tra_sz1_it2_over300.outTestStats	weka.classifiers.lazy.LWL	[-K, 90, -A, weka.core.neighboursearch.LinearNNSearch, -W, weka.classifiers.bayes.NaiveBayes, --, -D]	null	[]	null	[]
raw_8yesSC_con_tra_sz1_it3.outTestStats	weka.classifiers.meta.RandomCommittee	[-I, 47, -S, 1, -W, weka.classifiers.trees.RandomForest, --, -I, 55, -K, 3, -depth, 0]	null	[]	null	[]
raw_8yesSC_con_tra_sz1_it3_over200.outTestStats	weka.classifiers.meta.RandomSubSpace	[-I, 48, -P, 0.7040799629891291, -S, 1, -W, weka.classifiers.bayes.BayesNet, --, -D, -Q, weka.classifiers.bayes.net.search.local.K2]	null	[]	null	[]
raw_8yesSC_con_tra_sz1_it3_over300.outTestStats	weka.classifiers.meta.AdaBoostM1	[-P, 100, -I, 23, -S, 1, -W, weka.classifiers.rules.PART, --, -M, 6, -B]	weka.attributeSelection.GreedyStepwise	[-N, 213]	weka.attributeSelection.CfsSubsetEval	[-L]
raw_8yesSC_con_tra_sz2_it1.outTestStats	weka.classifiers.lazy.KStar	[-B, 81, -M, a]	null	[]	null	[]
raw_8yesSC_con_tra_sz2_it2.outTestStats	weka.classifiers.meta.RandomSubSpace	[-I, 36, -P, 0.2757331204049802, -S, 1, -W, weka.classifiers.lazy.IBk, --, -E, -K, 10, -X]	null	[]	null	[]
raw_8yesSC_con_tra_sz2_it3.outTestStats	weka.classifiers.meta.RandomCommittee	[-I, 14, -S, 1, -W, weka.classifiers.trees.RandomForest, --, -I, 59, -K, 1, -depth, 0]	null	[]	null	[]
raw_8yesSC_con_tra_sz3_it1.outTestStats	weka.classifiers.trees.RandomForest	[-I, 159, -K, 26, -depth, 0]	null	[]	null	[]
raw_8yesSC_con_tra_sz3_it2.outTestStats	weka.classifiers.meta.RandomCommittee	[-I, 14, -S, 1, -W, weka.classifiers.trees.RandomForest, --, -I, 59, -K, 1, -depth, 0]	null	[]	null	[]

raw_8yesSC_con_tra_sz3_it3.outTestStats	weka.classifiers.meta.RandomCommittee	[-I, 38, -S, 1, -W, weka.classifiers.trees.RandomTree, --, -M, 2, -K, 3, -depth, 20, -N, 0]	weka.attributeSelection.GreedyStepwise	[-C, -B, -R]	weka.attributeSelection.CfsSubsetEval	[-M, -L]
raw_8yesSC_sum_tra_sz1_it1.outTestStats	weka.classifiers.lazy.LWL	[-U, 1, -A, weka.core.neighboursearch.LinearNNSearch, -W, weka.classifiers.functions.SMO, --, -C, 1.376271748154589, -N, 0, -M, -K, weka.classifiers.functions.supportVector.Puk -S 0.3024853148573099 -O 0.6878560663513378]	null	[]	null	[]
raw_8yesSC_sum_tra_sz1_it1_over200.outTestStats	weka.classifiers.meta.AdaBoostM1	[-P, 100, -I, 123, -Q, -S, 1, -W, weka.classifiers.rules.PART, --, -N, 3, -M, 4, -R, -B]	null	[]	null	[]
raw_8yesSC_sum_tra_sz1_it1_over300.outTestStats	weka.classifiers.meta.AdaBoostM1	[-P, 72, -I, 116, -S, 1, -W, weka.classifiers.trees.RandomTree, --, -M, 3, -K, 0, -depth, 0, -N, 0, -U]	null	[]	null	[]
raw_8yesSC_sum_tra_sz1_it2.outTestStats	weka.classifiers.meta.AdaBoostM1	[-P, 100, -I, 123, -Q, -S, 1, -W, weka.classifiers.trees.RandomTree, --, -M, 7, -K, 0, -depth, 0, -N, 4]	null	[]	null	[]
raw_8yesSC_sum_tra_sz1_it2_over200.outTestStats	weka.classifiers.meta.RandomCommittee	[-I, 53, -S, 1, -W, weka.classifiers.trees.RandomTree, --, -M, 5, -K, 6, -depth, 0, -N, 0, -U]	weka.attributeSelection.GreedyStepwise	[-C, -R]	weka.attributeSelection.CfsSubsetEval	[-M]
raw_8yesSC_sum_tra_sz1_it2_over300.outTestStats	weka.classifiers.lazy.LWL	[-K, 30, -A, weka.core.neighboursearch.LinearNNSearch, -W, weka.classifiers.functions.MultilayerPerceptron, --, -L, 0.5111151054851913, -M, 0.10388719485323533, -H, i, -C, -S, 1]	weka.attributeSelection.GreedyStepwise	[-B, -R]	weka.attributeSelection.CfsSubsetEval	[-M]
raw_8yesSC_sum_tra_sz1_it3.outTestStats	weka.classifiers.meta.AdaBoostM1	[-P, 100, -I, 64, -S, 1, -W, weka.classifiers.trees.LMT, --, -B, -R, -P, -M, 1, -W, 0]	null	[]	null	[]
raw_8yesSC_sum_tra_sz1_it3_over200.outTestStats	weka.classifiers.lazy.LWL	[-K, 120, -A, weka.core.neighboursearch.LinearNNSearch, -W, weka.classifiers.functions.MultilayerPerceptron, --, -L, 0.18091995356647095, -M,	null	[]	null	[]

		0.6002222647449894, -B, -H, o, -C, -S, 1]				
raw_8yesSC_sum_tra_sz1_it3_over300.outTestStats	weka.classifiers.meta.AdaBoostM1	[-P, 100, -I, 54, -S, 1, -W, weka.classifiers.trees.RandomForest, --, -I, 2, -K, 0, -depth, 14]	weka.attributeSelection.GreedyStepwise	[-C, -R]	weka.attributeSelection.CfsSubsetEval	[-L]
raw_8yesSC_sum_tra_sz2_it1.outTestStats	weka.classifiers.meta.AdaBoostM1	[-P, 100, -I, 53, -S, 1, -W, weka.classifiers.rules.PART, --, -M, 1]	weka.attributeSelection.GreedyStepwise	[-B, -R]	weka.attributeSelection.CfsSubsetEval	[-L]
raw_8yesSC_sum_tra_sz2_it2.outTestStats	weka.classifiers.meta.AdaBoostM1	[-P, 100, -I, 76, -Q, -S, 1, -W, weka.classifiers.rules.JRip, --, -N, 2.851960831017474, -E, -P, -O, 4]	null	[]	null	[]
raw_8yesSC_sum_tra_sz2_it3.outTestStats	weka.classifiers.lazy.LWL	[-K, 120, -A, weka.core.neighboursearch.LinearNNSearch, -W, weka.classifiers.functions.MultilayerPerceptron, --, -L, 0.3096497000508256, -M, 0.7715921726163002, -H, a, -R, -D, -S, 1]	null	[]	null	[]
raw_8yesSC_sum_tra_sz3_it1.outTestStats	weka.classifiers.meta.RandomCommittee	[-I, 14, -S, 1, -W, weka.classifiers.trees.RandomForest, --, -I, 59, -K, 1, -depth, 0]	null	[]	null	[]
raw_8yesSC_sum_tra_sz3_it2.outTestStats	weka.classifiers.meta.AdaBoostM1	[-P, 83, -I, 14, -Q, -S, 1, -W, weka.classifiers.trees.RandomForest, --, -I, 63, -K, 0, -depth, 0]	null	[]	null	[]
raw_8yesSC_sum_tra_sz3_it3.outTestStats	weka.classifiers.meta.RandomCommittee	[-I, 9, -S, 1, -W, weka.classifiers.trees.RandomForest, --, -I, 55, -K, 0, -depth, 17]	null	[]	null	[]
stand_7noSC_con_tra_sz1_it1.outTestStats	weka.classifiers.meta.RandomCommittee	[-I, 56, -S, 1, -W, weka.classifiers.trees.RandomTree, --, -M, 2, -K, 9, -depth, 0, -N, 0]	weka.attributeSelection.GreedyStepwise	[-C, -R]	weka.attributeSelection.CfsSubsetEval	[-M]
stand_7noSC_con_tra_sz1_it1_over200.outTestStats	weka.classifiers.meta.AdaBoostM1	[-P, 75, -I, 13, -Q, -S, 1, -W, weka.classifiers.lazy.KStar, --, -B, 53, -M, d]	null	[]	null	[]
stand_7noSC_con_tra_sz1_it1_over300.outTestStats	weka.classifiers.lazy.IBk	[-E, -K, 2]	weka.attributeSelection.GreedyStepwise	[-C, -R]	weka.attributeSelection.CfsSubsetEval	[]
stand_7noSC_con_tra_sz1_it2.outTestStats	weka.classifiers.meta.AdaBoostM1	[-P, 100, -I, 29, -S, 1, -W, weka.classifiers.trees.LMT, --, -B, -M, 1, -W, 0.23472238723329764, -A]	null	[]	null	[]
stand_7noSC_con_tra_sz1_it2_over200.outTestStats	weka.classifiers.lazy.LWL	[-K, 60, -A, weka.core.neighboursearch.LinearNNSearch, -W, weka.classifiers.functions.Logistic, --, -R,	null	[]	null	[]

		0.3667156826637985]				
stand_7noSC_con_tra_sz1_it2_over300.outTestStats	weka.classifiers.bayes.BayesNet	[-D, -Q, weka.classifiers.bayes.net.search.local.LAGDHillClimber]	null	[]	null	[]
stand_7noSC_con_tra_sz1_it3.outTestStats	weka.classifiers.lazy.KStar	[-B, 48, -M, d]	null	[]	null	[]
stand_7noSC_con_tra_sz1_it3_over200.outTestStats	weka.classifiers.lazy.IBk	[-E, -K, 2, -X]	null	[]	null	[]
stand_7noSC_con_tra_sz1_it3_over300.outTestStats	weka.classifiers.lazy.IBk	[-K, 2]	null	[]	null	[]
stand_7noSC_con_tra_sz2_it1.outTestStats	weka.classifiers.lazy.KStar	[-B, 48, -M, n]	null	[]	null	[]
stand_7noSC_con_tra_sz2_it2.outTestStats	weka.classifiers.meta.RandomSubSpace	[-I, 19, -P, 0.8528725896677177, -S, 1, -W, weka.classifiers.trees.RandomTree, --, -M, 1, -K, 8, -depth, 0, -N, 0]	null	[]	null	[]
stand_7noSC_con_tra_sz2_it3.outTestStats	weka.classifiers.meta.RandomCommittee	[-I, 9, -S, 1, -W, weka.classifiers.trees.RandomForest, --, -I, 49, -K, 0, -depth, 0]	weka.attributeSelection.GreedyStepwise	[-C, -R]	weka.attributeSelection.CfsSubsetEval	[-L]
stand_7noSC_con_tra_sz3_it1.outTestStats	weka.classifiers.meta.Vote	[-R, PROD, -S, 1, -B, weka.classifiers.lazy.KStar -B 43 -M m, -B, weka.classifiers.functions.SimpleLogistic -S -W 0.9238405636892598, -B, weka.classifiers.rules.DecisionTable -E rmse -S weka.attributeSelection.BestFirst -X 4, -B, weka.classifiers.rules.DecisionTable -E acc -I -S weka.attributeSelection.BestFirst -X 1]	null	[]	null	[]
stand_7noSC_con_tra_sz3_it2.outTestStats	weka.classifiers.meta.RandomSubSpace	[-I, 36, -P, 0.2757331204049802, -S, 1, -W, weka.classifiers.lazy.IBk, --, -E, -K, 10, -X]	null	[]	null	[]
stand_7noSC_con_tra_sz3_it3.outTestStats	weka.classifiers.meta.RandomSubSpace	[-I, 19, -P, 0.8528725896677177, -S, 1, -W, weka.classifiers.trees.RandomTree, --, -M, 1, -K, 8, -depth, 0, -N, 0]	null	[]	null	[]
stand_7noSC_sum_tra_sz1_it1.outTestStats	weka.classifiers.meta.AdaBoostM1	[-P, 100, -I, 49, -S, 1, -W, weka.classifiers.trees.RandomTree, --, -M, 14, -K, 4, -depth, 8, -N, 0]	null	[]	null	[]
stand_7noSC_sum_tra_sz1_it1_over200.outTestStats	weka.classifiers.meta.AdaBoostM1	[-P, 100, -I, 40, -Q, -S, 1, -W, weka.classifiers.trees.J48, --, -B, -J, -M, 1, -C,	weka.attributeSelection.GreedyStepwise	[-C, -B, -R]	weka.attributeSelection.CfsSubsetEval	[-M]

		0.37633189036368353]				
stand_7noSC_sum_tra_sz1_it1_over300.outTestStats	weka.classifiers.meta.RandomCommittee	[-I, 5, -S, 1, -W, weka.classifiers.trees.RandomForest, --, -I, 107, -K, 0, -depth, 0]	null	[]	null	[]
stand_7noSC_sum_tra_sz1_it2.outTestStats	weka.classifiers.lazy.LWL	[-U, 1, -A, weka.core.neighboursearch.LinearNNSearch, -W, weka.classifiers.trees.RandomForest, --, -I, 243, -K, 0, -depth, 20]	weka.attributeSelection.GreedyStepwise	[-B, -R]	weka.attributeSelection.CfsSubsetEval	[-M, -L]
stand_7noSC_sum_tra_sz1_it2_over200.outTestStats	weka.classifiers.lazy.LWL	[-K, 120, -A, weka.core.neighboursearch.LinearNNSearch, -W, weka.classifiers.functions.SimpleLogistic, --, -W, 0]	weka.attributeSelection.GreedyStepwise	[-R]	weka.attributeSelection.CfsSubsetEval	[]
stand_7noSC_sum_tra_sz1_it2_over300.outTestStats	weka.classifiers.lazy.LWL	[-K, 30, -A, weka.core.neighboursearch.LinearNNSearch, -W, weka.classifiers.functions.Logistic, --, -R, 0.0847257114324799]	weka.attributeSelection.GreedyStepwise	[-B, -R]	weka.attributeSelection.CfsSubsetEval	[-M]
stand_7noSC_sum_tra_sz1_it3.outTestStats	weka.classifiers.meta.AdaBoostM1	[-P, 100, -I, 39, -S, 1, -W, weka.classifiers.trees.RandomTree, --, -M, 5, -K, 25, -depth, 8, -N, 0, -U]	null	[]	null	[]
stand_7noSC_sum_tra_sz1_it3_over200.outTestStats	weka.classifiers.meta.AdaBoostM1	[-P, 91, -I, 124, -S, 1, -W, weka.classifiers.trees.RandomForest, --, -I, 2, -K, 1, -depth, 0]	null	[]	null	[]
stand_7noSC_sum_tra_sz1_it3_over300.outTestStats	weka.classifiers.meta.AdaBoostM1	[-P, 84, -I, 25, -Q, -S, 1, -W, weka.classifiers.trees.RandomForest, --, -I, 48, -K, 5, -depth, 0]	null	[]	null	[]
stand_7noSC_sum_tra_sz2_it1.outTestStats	weka.classifiers.meta.AdaBoostM1	[-P, 100, -I, 31, -Q, -S, 1, -W, weka.classifiers.trees.RandomForest, --, -I, 3, -K, 0, -depth, 17]	null	[]	null	[]
stand_7noSC_sum_tra_sz2_it2.outTestStats	weka.classifiers.meta.AdaBoostM1	[-P, 100, -I, 18, -Q, -S, 1, -W, weka.classifiers.trees.RandomForest, --, -I, 26, -K, 7, -depth, 0]	null	[]	null	[]
stand_7noSC_sum_tra_sz2_it3.outTestStats	weka.classifiers.meta.AdaBoostM1	[-P, 100, -I, 67, -S, 1, -W, weka.classifiers.trees.RandomForest, --, -I, 183, -K, 1, -depth, 19]	null	[]	null	[]
stand_7noSC_sum_tra_sz3_it1.outTestStats	weka.classifiers.meta.AdaBoostM1	[-P, 100, -I, 8, -Q, -S, 1, -W, weka.classifiers.trees.RandomForest, --, -I, 84, -K, 0, -depth, 0]	null	[]	null	[]
stand_7noSC_sum_tra_sz3_it2.outTestStats	weka.classifiers.meta.AdaBoostM1	[-P, 100, -I, 23, -Q, -S, 1, -W, weka.classifiers.trees.RandomForest, --, -I,	null	[]	null	[]

		191, -K, 0, -depth, 12]				
stand_7noSC_sum_tra_sz3_it3.outTestStats	weka.classifiers.meta.AdaBoostM1	[-P, 100, -I, 64, -S, 1, -W, weka.classifiers.trees.LMT, --, -B, -R, -P, -M, 1, -W, 0]	null	[]	null	[]
stand_7yesSC_con_tra_sz1_it1.outTestStats	weka.classifiers.meta.AdaBoostM1	[-P, 100, -I, 45, -Q, -S, 1, -W, weka.classifiers.trees.RandomForest, --, -I, 18, -K, 7, -depth, 8]	weka.attributeSelection.GreedyStepwise	[-C, -R]	weka.attributeSelection.CfsSubsetEval	[-M, -L]
stand_7yesSC_con_tra_sz1_it1_over200.outTestStats	weka.classifiers.lazy.KStar	[-B, 34, -M, n]	null	[]	null	[]
stand_7yesSC_con_tra_sz1_it1_over300.outTestStats	weka.classifiers.lazy.IBk	[-E, -K, 1, -X, -F]	null	[]	null	[]
stand_7yesSC_con_tra_sz1_it2.outTestStats	weka.classifiers.meta.Vote	[-R, MAX, -S, 1, -B, weka.classifiers.lazy.KStar -B 49 -M m, -B, weka.classifiers.trees.LMT -B -R -C -M 1 -W 0.2316048408449548, -B, weka.classifiers.rules.JRip -N 4.97832459259406 -E -O 1, -B, weka.classifiers.rules.DecisionTable -E rmse -I -S weka.attributeSelection.BestFirst -X 3]	weka.attributeSelection.GreedyStepwise	[-B, -R]	weka.attributeSelection.CfsSubsetEval	[-M, -L]
stand_7yesSC_con_tra_sz1_it2_over200.outTestStats	weka.classifiers.lazy.KStar	[-B, 38, -M, a]	null	[]	null	[]
stand_7yesSC_con_tra_sz1_it2_over300.outTestStats	weka.classifiers.meta.RandomCommittee	[-I, 17, -S, 1, -W, weka.classifiers.trees.RandomTree, --, -M, 1, -K, 0, -depth, 17, -N, 0, -U]	null	[]	null	[]
stand_7yesSC_con_tra_sz1_it3.outTestStats	weka.classifiers.lazy.KStar	[-B, 85, -M, a]	null	[]	null	[]
stand_7yesSC_con_tra_sz1_it3_over200.outTestStats	weka.classifiers.lazy.LWL	[-K, 60, -A, weka.core.neighboursearch.LinearNNSearch, -W, weka.classifiers.bayes.NaiveBayes, --, -D]	null	[]	null	[]
stand_7yesSC_con_tra_sz1_it3_over300.outTestStats	weka.classifiers.lazy.IBk	[-K, 5, -X]	null	[]	null	[]
stand_7yesSC_con_tra_sz2_it1.outTestStats	weka.classifiers.meta.AdaBoostM1	[-P, 100, -I, 104, -S, 1, -W, weka.classifiers.rules.JRip, --, -N, 4.967805931437204, -O, 3]	weka.attributeSelection.BestFirst	[-D, 0, -N, 10]	weka.attributeSelection.CfsSubsetEval	[-L]
stand_7yesSC_con_tra_sz2_it2.outTestStats	weka.classifiers.meta.Bagging	[-P, 51, -I, 125, -S, 1, -W, weka.classifiers.lazy.KStar, --, -B, 70, -E, -M, m]	weka.attributeSelection.GreedyStepwise	[-R]	weka.attributeSelection.CfsSubsetEval	[]
stand_7yesSC_con_tra_sz2_it3.outTestStats	weka.classifiers.lazy.KStar	[-B, 23, -M, d]	weka.attributeSelection.GreedySte	[-C, -B, -R]	weka.attributeSelection.CfsSub	[-L]

			pwise		setEval	
stand_7yesSC_con_tra_sz3_it1.outTestStats	weka.classifiers.meta.RandomSubSpace	[-I, 3, -P, 0.5502323068139516, -S, 1, -W, weka.classifiers.lazy.KStar, --, -B, 67, -M, d]	weka.attributeSelection.GreedyStepwise	[-B, -R]	weka.attributeSelection.CfsSubsetEval	[-L]
stand_7yesSC_con_tra_sz3_it2.outTestStats	weka.classifiers.meta.RandomSubSpace	[-I, 36, -P, 0.2757331204049802, -S, 1, -W, weka.classifiers.lazy.IBk, --, -E, -K, 10, -X]	null	[]	null	[]
stand_7yesSC_con_tra_sz3_it3.outTestStats	weka.classifiers.meta.AdaBoostM1	[-P, 100, -I, 127, -Q, -S, 1, -W, weka.classifiers.trees.REPTree, --, -M, 3, -V, 5.839517683122976E-5, -L, 17, -P]	weka.attributeSelection.GreedyStepwise	[-R]	weka.attributeSelection.CfsSubsetEval	[-L]
stand_7yesSC_sum_tra_sz1_it1.outTestStats	weka.classifiers.meta.AdaBoostM1	[-P, 100, -I, 73, -S, 1, -W, weka.classifiers.trees.RandomForest, --, -I, 170, -K, 1, -depth, 19]	null	[]	null	[]
stand_7yesSC_sum_tra_sz1_it1_over200.outTestStats	weka.classifiers.meta.AdaBoostM1	[-P, 66, -I, 33, -Q, -S, 1, -W, weka.classifiers.trees.RandomForest, --, -I, 37, -K, 1, -depth, 0]	null	[]	null	[]
stand_7yesSC_sum_tra_sz1_it1_over300.outTestStats	weka.classifiers.meta.AdaBoostM1	[-P, 82, -I, 71, -S, 1, -W, weka.classifiers.trees.REPTree, --, -M, 2, -V, 5.7467452548641326E-5, -L, -1, -P]	null	[]	null	[]
stand_7yesSC_sum_tra_sz1_it2.outTestStats	weka.classifiers.lazy.LWL	[-K, 60, -A, weka.core.neighboursearch.LinearNNSearch, -W, weka.classifiers.bayes.NaiveBayes, --]	null	[]	null	[]
stand_7yesSC_sum_tra_sz1_it2_over200.outTestStats	weka.classifiers.meta.AdaBoostM1	[-P, 100, -I, 68, -Q, -S, 1, -W, weka.classifiers.trees.REPTree, --, -M, 3, -V, 0.0031733230666502917, -L, -1, -P]	null	[]	null	[]
stand_7yesSC_sum_tra_sz1_it2_over300.outTestStats	weka.classifiers.meta.AdaBoostM1	[-P, 100, -I, 21, -S, 1, -W, weka.classifiers.trees.J48, --, -J, -A, -S, -M, 1]	weka.attributeSelection.GreedyStepwise	[-B, -R]	weka.attributeSelection.CfsSubsetEval	[-L]
stand_7yesSC_sum_tra_sz1_it3.outTestStats	weka.classifiers.meta.AdaBoostM1	[-P, 100, -I, 63, -Q, -S, 1, -W, weka.classifiers.trees.RandomForest, --, -I, 39, -K, 0, -depth, 0]	null	[]	null	[]
stand_7yesSC_sum_tra_sz1_it3_over200.outTestStats	weka.classifiers.meta.AdaBoostM1	[-P, 100, -I, 127, -S, 1, -W, weka.classifiers.trees.J48, --, -A, -S, -M, 12, -C, 0.8219794409734856]	null	[]	null	[]
stand_7yesSC_sum_tra_sz1_it3_over300.outTestStats	weka.classifiers.meta.AdaBoostM1	[-P, 100, -I, 104, -S, 1, -W, weka.classifiers.trees.REPTree, --, -M, 9, -V, 0.02770389319636118,	weka.attributeSelection.GreedyStepwise	[-C, -B, -R]	weka.attributeSelection.CfsSubsetEval	[-L]

		-L, 9, -P]				
stand_7yesSC_sum_tra_sz2_it1.outTestStats	weka.classifiers.functions.SMO	[-C, 0.8834901573983901, -N, 1, -K, weka.classifiers.functions.supportVector.Puk -S 2.70071737141395 -O 0.7960004287380918]	weka.attributeSelection.GreedyStepwise	[-B, -R]	weka.attributeSelection.CfsSubsetEval	[-M]
stand_7yesSC_sum_tra_sz2_it2.outTestStats	weka.classifiers.meta.AdaBoostM1	[-P, 100, -I, 15, -S, 1, -W, weka.classifiers.trees.RandomForest, --, -I, 12, -K, 0, -depth, 18]	null	[]	null	[]
stand_7yesSC_sum_tra_sz2_it3.outTestStats	weka.classifiers.meta.AdaBoostM1	[-P, 100, -I, 57, -Q, -S, 1, -W, weka.classifiers.trees.J48, --, -B, -M, 2, -C, 0.9253621371261777]	null	[]	null	[]
stand_7yesSC_sum_tra_sz3_it1.outTestStats	weka.classifiers.meta.RandomCommittee	[-I, 16, -S, 1, -W, weka.classifiers.trees.RandomForest, --, -I, 48, -K, 3, -depth, 0]	weka.attributeSelection.GreedyStepwise	[-B, -R]	weka.attributeSelection.CfsSubsetEval	[-L]
stand_7yesSC_sum_tra_sz3_it2.outTestStats	weka.classifiers.meta.AdaBoostM1	[-P, 100, -I, 64, -S, 1, -W, weka.classifiers.trees.RandomForest, --, -I, 4, -K, 0, -depth, 15]	null	[]	null	[]
stand_7yesSC_sum_tra_sz3_it3.outTestStats	weka.classifiers.meta.AdaBoostM1	[-P, 100, -I, 83, -Q, -S, 1, -W, weka.classifiers.trees.RandomTree, --, -M, 1, -K, 9, -depth, 17, -N, 5]	weka.attributeSelection.GreedyStepwise	[-C, -R]	weka.attributeSelection.CfsSubsetEval	[-M, -L]
stand_8noSC_con_tra_sz1_it1.outTestStats	weka.classifiers.lazy.KStar	[-B, 6, -M, m]	null	[]	null	[]
stand_8noSC_con_tra_sz1_it1_over200.outTestStats	weka.classifiers.lazy.LWL	[-K, 120, -A, weka.core.neighboursearch.LinearNNSearch, -W, weka.classifiers.functions.MultilayerPerceptron, --, -L, 0.3096497000508256, -M, 0.7715921726163002, -H, a, -R, -D, -S, 1]	null	[]	null	[]
stand_8noSC_con_tra_sz1_it1_over300.outTestStats	weka.classifiers.bayes.BayesNet	[-Q, weka.classifiers.bayes.net.search.local.LAGDHillClimber]	null	[]	null	[]
stand_8noSC_con_tra_sz1_it2.outTestStats	weka.classifiers.lazy.LWL	[-A, weka.core.neighboursearch.LinearNNSearch, -W, weka.classifiers.trees.RandomForest, --, -I, 27, -K, 0, -depth, 0]	null	[]	null	[]
stand_8noSC_con_tra_sz1_it2_over200.outTestStats	weka.classifiers.lazy.LWL	[-K, 60, -A, weka.core.neighboursearch.LinearNNSearch, -W, weka.classifiers.functions.Logistic, --, -R, 1.6731268020936618E-5]	null	[]	null	[]

stand_8noSC_con_tra_sz1_it2_over300.outTestStats	weka.classifiers.meta.RandomSubSpace	[-I, 20, -P, 0.9025796450171325, -S, 1, -W, weka.classifiers.bayes.BayesNet, --, -Q, weka.classifiers.bayes.net.search.local.K2]	weka.attributeSelection.GreedyStepwise	[-R]	weka.attributeSelection.CfsSubsetEval	[-M]
stand_8noSC_con_tra_sz1_it3.outTestStats	weka.classifiers.meta.AdaBoostM1	[-P, 100, -I, 23, -S, 1, -W, weka.classifiers.rules.PART, --, -M, 6, -B]	weka.attributeSelection.GreedyStepwise	[-N, 213]	weka.attributeSelection.CfsSubsetEval	[-L]
stand_8noSC_con_tra_sz1_it3_over200.outTestStats	weka.classifiers.meta.RandomSubSpace	[-I, 19, -P, 0.8528725896677177, -S, 1, -W, weka.classifiers.trees.RandomTree, --, -M, 1, -K, 8, -depth, 0, -N, 0]	null	[]	null	[]
stand_8noSC_con_tra_sz1_it3_over300.outTestStats	weka.classifiers.lazy.LWL	[-K, 60, -A, weka.core.neighboursearch.LinearNNSearch, -W, weka.classifiers.functions.SimpleLogistic, --, -S, -W, 0.3778879517835153, -A]	weka.attributeSelection.GreedyStepwise	[-R]	weka.attributeSelection.CfsSubsetEval	[]
stand_8noSC_con_tra_sz2_it1.outTestStats	weka.classifiers.lazy.IBk	[-K, 15, -X]	null	[]	null	[]
stand_8noSC_con_tra_sz2_it2.outTestStats	weka.classifiers.lazy.KStar	[-B, 71, -M, n]	null	[]	null	[]
stand_8noSC_con_tra_sz2_it3.outTestStats	weka.classifiers.lazy.LWL	[-A, weka.core.neighboursearch.LinearNNSearch, -W, weka.classifiers.trees.RandomForest, --, -I, 81, -K, 0, -depth, 14]	null	[]	null	[]
stand_8noSC_con_tra_sz3_it1.outTestStats	weka.classifiers.trees.RandomForest	[-I, 159, -K, 26, -depth, 0]	null	[]	null	[]
stand_8noSC_con_tra_sz3_it2.outTestStats	weka.classifiers.meta.RandomSubSpace	[-I, 36, -P, 0.2685536367337107, -S, 1, -W, weka.classifiers.trees.RandomForest, --, -I, 47, -K, 9, -depth, 17]	weka.attributeSelection.BestFirst	[-D, 0, -N, 7]	weka.attributeSelection.CfsSubsetEval	[-L]
stand_8noSC_con_tra_sz3_it3.outTestStats	weka.classifiers.meta.RandomSubSpace	[-I, 46, -P, 0.3209433034929777, -S, 1, -W, weka.classifiers.trees.RandomTree, --, -M, 1, -K, 0, -depth, 0, -N, 0, -U]	weka.attributeSelection.BestFirst	[-D, 2, -N, 7]	weka.attributeSelection.CfsSubsetEval	[]
stand_8noSC_sum_tra_sz1_it1.outTestStats	weka.classifiers.meta.AdaBoostM1	[-P, 100, -I, 36, -S, 1, -W, weka.classifiers.trees.RandomForest, --, -I, 19, -K, 1, -depth, 18]	null	[]	null	[]
stand_8noSC_sum_tra_sz1_it1_over200.outTestStats	weka.classifiers.meta.RandomCommittee	[-I, 37, -S, 1, -W, weka.classifiers.trees.RandomForest, --, -I, 68, -K, 0, -depth, 19]	weka.attributeSelection.GreedyStepwise	[-B, -R]	weka.attributeSelection.CfsSubsetEval	[-L]
stand_8noSC_sum_tra_sz1_it1_over300.outTestStats	weka.classifiers.meta.RandomCommittee	[-I, 57, -S, 1, -W, weka.classifiers.trees.RandomForest, --, -I, 5, -K, 0, -depth, 20]	null	[]	null	[]

stand_8noSC_sum_tra_sz1_it2.outTestStats	weka.classifiers.meta.AdaBoostM1	[-P, 100, -I, 83, -Q, -S, 1, -W, weka.classifiers.trees.J48, --, -B, -J, -A, -M, 4, -C, 0.35683272170421754]	weka.attributeSelection.GreedyStepwise	[-R]	weka.attributeSelection.CfsSubsetEval	[-L]
stand_8noSC_sum_tra_sz1_it2_over200.outTestStats	weka.classifiers.meta.AdaBoostM1	[-P, 100, -I, 52, -Q, -S, 1, -W, weka.classifiers.rules.JRip, --, -N, 1.5153262950720277, -O, 5]	weka.attributeSelection.GreedyStepwise	[-C, -B, -R]	weka.attributeSelection.CfsSubsetEval	[-M, -L]
stand_8noSC_sum_tra_sz1_it2_over300.outTestStats	weka.classifiers.meta.AdaBoostM1	[-P, 100, -I, 89, -Q, -S, 1, -W, weka.classifiers.trees.RandomForest, --, -I, 41, -K, 0, -depth, 7]	null	[]	null	[]
stand_8noSC_sum_tra_sz1_it3.outTestStats	weka.classifiers.meta.AdaBoostM1	[-P, 100, -I, 76, -Q, -S, 1, -W, weka.classifiers.rules.JRip, --, -N, 2.851960831017474, -E, -P, -O, 4]	null	[]	null	[]
stand_8noSC_sum_tra_sz1_it3_over200.outTestStats	weka.classifiers.lazy.LWL	[-A, weka.core.neighboursearch.LinearNNSearch, -W, weka.classifiers.functions.SMO, --, -C, 1.4072796335738316, -N, 1, -M, -K, weka.classifiers.functions.supportVector.Puk -S 9.994161456055084 -O 0.631362062367872]	null	[]	null	[]
stand_8noSC_sum_tra_sz1_it3_over300.outTestStats	weka.classifiers.meta.AdaBoostM1	[-P, 82, -I, 95, -S, 1, -W, weka.classifiers.trees.RandomForest, --, -I, 203, -K, 0, -depth, 8]	null	[]	null	[]
stand_8noSC_sum_tra_sz2_it1.outTestStats	weka.classifiers.lazy.LWL	[-K, 30, -A, weka.core.neighboursearch.LinearNNSearch, -W, weka.classifiers.functions.Logistic, --, -R, 0.4922334523233597]	null	[]	null	[]
stand_8noSC_sum_tra_sz2_it2.outTestStats	weka.classifiers.lazy.LWL	[-U, 4, -A, weka.core.neighboursearch.LinearNNSearch, -W, weka.classifiers.functions.SMO, --, -C, 1.4296431262588447, -N, 1, -K, weka.classifiers.functions.supportVector.Puk -S 3.828431509993488 -O 0.8291772469375045]	null	[]	null	[]
stand_8noSC_sum_tra_sz2_it3.outTestStats	weka.classifiers.meta.AdaBoostM1	[-P, 100, -I, 127, -Q, -S, 1, -W, weka.classifiers.trees.RandomTree, --, -M, 1, -K, 0, -depth, 0, -N,	null	[]	null	[]

		0, -U]				
stand_8noSC_sum_tra_sz3_it1.outTestStats	weka.classifiers.lazy.LWL	[-K, 120, -A, weka.core.neighboursearch.LinearNNSearch, -W, weka.classifiers.functions.SimpleLogistic, --, -W, 0]	null	[]	null	[]
stand_8noSC_sum_tra_sz3_it2.outTestStats	weka.classifiers.meta.AdaBoostM1	[-P, 99, -I, 74, -S, 1, -W, weka.classifiers.trees.J48, --, -O, -B, -S, -M, 3]	null	[]	null	[]
stand_8noSC_sum_tra_sz3_it3.outTestStats	weka.classifiers.lazy.LWL	[-K, 60, -A, weka.core.neighboursearch.LinearNNSearch, -W, weka.classifiers.functions.MultilayerPerceptron, --, -L, 0.45973882535272725, -M, 0.6807468377123407, -B, -H, i, -R, -D, -S, 1]	weka.attributeSelection.GreedyStepwise	[-R]	weka.attributeSelection.CfsSubsetEval	[-M]
stand_8yesSC_con_tra_sz1_it1.outTestStats	weka.classifiers.meta.AdaBoostM1	[-P, 100, -I, 98, -S, 1, -W, weka.classifiers.rules.DecisionTable, --, -E, acc, -S, weka.attributeSelection.BestFirst, -X, 3]	weka.attributeSelection.GreedyStepwise	[-R]	weka.attributeSelection.CfsSubsetEval	[]
stand_8yesSC_con_tra_sz1_it1_over200.outTestStats	weka.classifiers.lazy.IBk	[-E, -K, 2, -X]	null	[]	null	[]
stand_8yesSC_con_tra_sz1_it1_over300.outTestStats	weka.classifiers.lazy.IBk	[-E, -K, 34, -X, -I]	null	[]	null	[]
stand_8yesSC_con_tra_sz1_it2.outTestStats	weka.classifiers.meta.AdaBoostM1	[-P, 53, -I, 123, -Q, -S, 1, -W, weka.classifiers.trees.RandomForest, --, -I, 187, -K, 0, -depth, 0]	null	[]	null	[]
stand_8yesSC_con_tra_sz1_it2_over200.outTestStats	weka.classifiers.meta.AdaBoostM1	[-P, 100, -I, 83, -Q, -S, 1, -W, weka.classifiers.trees.J48, --, -B, -J, -A, -M, 4, -C, 0.35683272170421754]	weka.attributeSelection.GreedyStepwise	[-R]	weka.attributeSelection.CfsSubsetEval	[-L]
stand_8yesSC_con_tra_sz1_it2_over300.outTestStats	weka.classifiers.meta.AdaBoostM1	[-P, 100, -I, 60, -Q, -S, 1, -W, weka.classifiers.rules.JRip, --, -N, 3.9197608301437943, -E, -O, 3]	null	[]	null	[]
stand_8yesSC_con_tra_sz1_it3.outTestStats	weka.classifiers.meta.AdaBoostM1	[-P, 100, -I, 66, -S, 1, -W, weka.classifiers.trees.J48, --, -B, -J, -M, 3]	weka.attributeSelection.BestFirst	[-D, 1, -N, 6]	weka.attributeSelection.CfsSubsetEval	[-M]
stand_8yesSC_con_tra_sz1_it3_over200.outTestStats	weka.classifiers.meta.AdaBoostM1	[-P, 100, -I, 65, -Q, -S, 1, -W, weka.classifiers.trees.REPTree, --, -M, 20, -V, 5.420545364909479E-5, -L, -1]	null	[]	null	[]
stand_8yesSC_con_tra_sz	weka.classifiers.laz	[-K, 2]	null	[]	null	[]

1_it3_over300.outTestStats	y.IBk					
stand_8yesSC_con_tra_sz2_it1.outTestStats	weka.classifiers.meta.Vote	[-R, MIN, -S, 1, -B, weka.classifiers.lazy.KStar -B 81 -M n]	weka.attributeSelection.GreedyStepwise	[-R]	weka.attributeSelection.CfsSubsetEval	[-M]
stand_8yesSC_con_tra_sz2_it2.outTestStats	weka.classifiers.meta.Vote	[-R, PROD, -S, 1, -B, weka.classifiers.lazy.KStar -B 43 -M m, -B, weka.classifiers.functions.SimpleLogistic -S -W 0.9238405636892598, -B, weka.classifiers.rules.DecisionTable -E rmse -S weka.attributeSelection.BestFirst -X 4, -B, weka.classifiers.rules.DecisionTable -E acc -I -S weka.attributeSelection.BestFirst -X 1]	null	[]	null	[]
stand_8yesSC_con_tra_sz2_it3.outTestStats	weka.classifiers.meta.Vote	[-R, PROD, -S, 1, -B, weka.classifiers.lazy.KStar -B 43 -M m, -B, weka.classifiers.functions.SimpleLogistic -S -W 0.9238405636892598, -B, weka.classifiers.rules.DecisionTable -E rmse -S weka.attributeSelection.BestFirst -X 4, -B, weka.classifiers.rules.DecisionTable -E acc -I -S weka.attributeSelection.BestFirst -X 1]	null	[]	null	[]
stand_8yesSC_con_tra_sz3_it1.outTestStats	weka.classifiers.meta.RandomCommittee	[-I, 39, -S, 1, -W, weka.classifiers.trees.RandomTree, --, -M, 2, -K, 4, -depth, 0, -N, 0, -U]	weka.attributeSelection.GreedyStepwise	[-C, -R]	weka.attributeSelection.CfsSubsetEval	[-M]
stand_8yesSC_con_tra_sz3_it2.outTestStats	weka.classifiers.meta.AdaBoostM1	[-P, 100, -I, 64, -Q, -S, 1, -W, weka.classifiers.trees.REPTree, --, -M, 2, -V, 1.4515133949647173E-5, -L, -1, -P]	weka.attributeSelection.GreedyStepwise	[-B, -R]	weka.attributeSelection.CfsSubsetEval	[]
stand_8yesSC_con_tra_sz3_it3.outTestStats	weka.classifiers.lazy.LWL	[-U, 3, -A, weka.core.neighboursearch.LinearNNSearch, -W, weka.classifiers.rules.DecisionTable, --, -E, acc, -S, weka.attributeSelection.BestFirst, -X, 1]	weka.attributeSelection.GreedyStepwise	[-B, -R]	weka.attributeSelection.CfsSubsetEval	[-L]
stand_8yesSC_sum_tra_sz1_it1.outTestStats	weka.classifiers.meta.AdaBoostM1	[-P, 92, -I, 113, -S, 1, -W, weka.classifiers.trees.REPTree, --, -M, 2, -V, 0.002032170211824805	weka.attributeSelection.GreedyStepwise	[-C, -R]	weka.attributeSelection.CfsSubsetEval	[]

		8, -L, -1]				
stand_8yesSC_sum_tra_sz1_it1_over200.outTestStats	weka.classifiers.meta.AdaBoostM1	[-P, 100, -I, 64, -S, 1, -W, weka.classifiers.trees.LMT, --, -B, -R, -P, -M, 1, -W, 0]	null	[]	null	[]
stand_8yesSC_sum_tra_sz1_it1_over300.outTestStats	weka.classifiers.meta.AdaBoostM1	[-P, 100, -I, 114, -S, 1, -W, weka.classifiers.trees.LMT, --, -R, -P, -M, 28, -W, 0, -A]	null	[]	null	[]
stand_8yesSC_sum_tra_sz1_it2.outTestStats	weka.classifiers.functions.SMO	[-C, 1.4706914365886425, -N, 1, -K, weka.classifiers.functions.supportVector.Puk -S 1.839051789593409 -O 0.8462457871776845]	null	[]	null	[]
stand_8yesSC_sum_tra_sz1_it2_over200.outTestStats	weka.classifiers.meta.AdaBoostM1	[-P, 67, -I, 126, -Q, -S, 1, -W, weka.classifiers.trees.RandomForest, --, -I, 187, -K, 0, -depth, 0]	null	[]	null	[]
stand_8yesSC_sum_tra_sz1_it2_over300.outTestStats	weka.classifiers.meta.AdaBoostM1	[-P, 100, -I, 76, -Q, -S, 1, -W, weka.classifiers.rules.JRip, --, -N, 2.851960831017474, -E, -P, -O, 4]	null	[]	null	[]
stand_8yesSC_sum_tra_sz1_it3.outTestStats	weka.classifiers.lazy.LWL	[-K, 60, -A, weka.core.neighboursearch.LinearNNSearch, -W, weka.classifiers.functions.Logistic, --, -R, 0.3667156826637985]	null	[]	null	[]
stand_8yesSC_sum_tra_sz1_it3_over200.outTestStats	weka.classifiers.meta.AdaBoostM1	[-P, 100, -I, 69, -Q, -S, 1, -W, weka.classifiers.trees.RandomTree, --, -M, 1, -K, 18, -depth, 0, -N, 4, -U]	null	[]	null	[]
stand_8yesSC_sum_tra_sz1_it3_over300.outTestStats	weka.classifiers.meta.AdaBoostM1	[-P, 100, -I, 33, -S, 1, -W, weka.classifiers.trees.RandomForest, --, -I, 13, -K, 0, -depth, 7]	null	[]	null	[]
stand_8yesSC_sum_tra_sz2_it1.outTestStats	weka.classifiers.meta.AdaBoostM1	[-P, 59, -I, 10, -Q, -S, 1, -W, weka.classifiers.trees.RandomForest, --, -I, 118, -K, 1, -depth, 15]	weka.attributeSelection.GreedyStepwise	[-C, -R]	weka.attributeSelection.CfsSubsetEval	[]
stand_8yesSC_sum_tra_sz2_it2.outTestStats	weka.classifiers.meta.AdaBoostM1	[-P, 100, -I, 31, -Q, -S, 1, -W, weka.classifiers.trees.RandomForest, --, -I, 28, -K, 2, -depth, 0]	null	[]	null	[]
stand_8yesSC_sum_tra_sz2_it3.outTestStats	weka.classifiers.meta.AdaBoostM1	[-P, 100, -I, 105, -Q, -S, 1, -W, weka.classifiers.trees.REPTree, --, -M, 1, -V, 4.780372348752996E-5, -L, -1, -P]	null	[]	null	[]
stand_8yesSC_sum_tra_sz3_it1.outTestStats	weka.classifiers.meta.AdaBoostM1	[-P, 100, -I, 23, -Q, -S, 1, -W,	null	[]	null	[]

stand_8yesSC_sum_tra_sz3_it2.outTestStats	weka.classifiers.meta.RandomSubSpace	weka.classifiers.trees.RandomForest, --, -I, 191, -K, 0, -depth, 12] [-I, 36, -P, 0.2757331204049802, -S, 1, -W, weka.classifiers.lazy.IBk, --, -E, -K, 10, -X]	null	[]	null	[]
stand_8yesSC_sum_tra_sz3_it3.outTestStats	weka.classifiers.meta.AdaBoostM1	[-P, 100, -I, 95, -Q, -S, 1, -W, weka.classifiers.trees.RandomTree, --, -M, 8, -K, 12, -depth, 0, -N, 4, -U]	null	[]	null	[]

The RCC column describes the conditions used to build the corresponding RCCs. Below is a summary scheme of the names in the RCC column:.

{norm/stand/raw}_{7/8}{no/yes}SC_{con/sum}_sz{1/2/3}_it{1/2/3}(_over{200/300}).

The fields are separated by an underscore. The first field {stand/norm/raw} specifies if the RCC were standardize, normalized or taken without further modification; The digits of the second field {7/8} indicates the distance in Angstroms used to build the contact map; {no/yes}SC inform if side-chain atoms were used or not in the construction of the contact map. The third field {con/sum} describe if concatenation or addition of each RCC for a protein-protein pair was implemented. From the fourth to the sixth field is the information of the undersampling procedure used, the sz{1/2/3} refers to the proportion of the undersampling of the majority class (positive) with reference to the length of the minority class (negative), as follows: sz1 = undersampling 1:1, sz2 = undersampling 2:1 and sz3 = undersampling 3:1 of positives (P) vs negatives (N) respectively; the fifth field it{1/2/3} refers to the random iteration seed used for the undersampling. Last, the sixth field (_over{200/300}) may or not be present, it is the indicative of the oversampling sets, which all part from an undersampling 1:1 set. In this way, the negative class was synthetically oversampled to generate the same quantity of negative instances 1:2 (P:N) (over200), or twice the quantity of negative instances 1:3 (P:N) (over300).
